# Novel Uridine Glycoconjugates, Derivatives of 4-Aminophenyl 1-Thioglycosides, as Potential Antiviral Compounds

**DOI:** 10.3390/molecules23061435

**Published:** 2018-06-13

**Authors:** Ewelina Krol, Gabriela Pastuch-Gawolek, Binay Chaubey, Gabriela Brzuska, Karol Erfurt, Boguslaw Szewczyk

**Affiliations:** 1 Department of Recombinant Vaccines, Intercollegiate Faculty of Biotechnology, University of Gdansk and Medical University of Gdansk, Abrahama 58, 80-307 Gdansk, Poland; bchaubey@hotmail.com (B.C.); gabriela.brzuska@phdstud.ug.edu.pl (G.B.); szewczyk@biotech.ug.gda.pl (B.S.); 2 Department of Organic Chemistry, Bioorganic Chemistry and Biotechnology, Faculty of Chemistry, Silesian University of Technology, Krzywoustego 4, 44-100 Gliwice, Poland; 3 Biotechnology Center, Silesian University of Technology, Krzywoustego 8, 44-100 Gliwice, Poland; 4 Functional Genomics Lab., Centre for Advanced Study, Department of Botany, University of Calcutta, 35, Ballygunge Circular Road, 700019 Kolkata, India; 5 Department of Chemical Organic Technology and Petrochemistry, Faculty of Chemistry, Silesian University of Technology, Krzywoustego 4, 44-100 Gliwice, Poland; karol.erfurt@polsl.pl

**Keywords:** hepatitis C virus, classical swine fever virus, antivirals, aryl 1-thioglycoside derivatives, uridine glycoconjugates, analogues of glycosyltransferase substrates

## Abstract

A novel series of uridine glycoconjugates, derivatives of 4-aminophenyl 1-thioglycosides, was designed and synthesized. All compounds were evaluated in vitro for their antiviral activity against hepatitis C virus (HCV) and classical swine fever virus (CSFV), two important human and animal viral pathogens for which new or improved therapeutic options are needed. The antiviral activity of all synthesized compounds was confirmed using pseudo-plaque reduction assays in which a significant arrest of CSFV and HCV growth was observed in the presence of these compounds. Two of the synthesized compounds, **9** and **12**, displayed a significant inhibitory effect on HCV and CSFV propagation with IC_50_ values of 4.9 and 13.5 µM for HCV and 4.2 and 4 µM for CSFV, respectively, with low cytotoxicity. Using various infection and replication models, we have shown that both compounds were able to significantly reduce viral genome replication by up to 90% with IC_50_ values in the low micromolar range. A structure activity analysis of the synthesized compounds showed that the high antiviral activity was attributed to the hydrophobicity of glycoconjugates and the introduction of elements capable to coordinate metal ions into the spacer connecting the sugar and uridine moiety, which can be useful in the development of new antiviral compounds in the future.

## 1. Introduction

Many human and animal viral infections are caused by RNA viruses belonging to the *Flaviviridae* family. This group includes viruses such as hepatitis C virus (HCV), dengue virus, Zika virus, tick-borne encephalitis virus, classical swine fever virus (CSFV), and many others. 

HCV is a serious global health problem affecting ~180 million people globally, corresponding to 3% of the world’s population, and it is a major cause of chronic liver diseases such as chronic hepatitis, liver cirrhosis, and hepatocellular carcinoma [1]. Until 2011, the standard of care has been a combination of pegylated interferon (PEG-IFN)-alpha and ribavirin; however, this is effective only to some extent and is associated with numerous side-effects [2]. In 2011, two HCV NS3/4A protease inhibitors, boceprevir and telaprevir, belonging to direct-acting antivirals (DAAs), were introduced to treat HCV genotype 1 patients together with PEG-IFN-alpha and ribavirin [2,3]. HCV treatment has been further improved with the approval of three new anti-HCV drugs: sofosbuvir (NS5B polymerase inhibitor), ledipasvir, and daclatasvir (both inhibitors of NS5A) [4]. Although sofosbuvir was the first drug used in combination with ribavirin in interferon-free therapy, it is effective only for genotype 2 and 3 HCV patients [5,6]. Ledipasvir is most commonly used in combination with sofosbuvir for treatment in chronic hepatitis C genotype 1 patients [7]. Daclatasvir is used only in combination therapy for the treatment of hepatitis C genotype 1, 3, or 4 infections together with sofosbuvir, ribavirin, and interferon [8]. In 2013, another drug, simeprevir (protease inhibitor), was approved by the Food and Drug Administration in the interferon-free regimen [9]. Since then, some other drugs belonging to NS3, NS5A, and NS5B inhibitors have been identified. 

Because of the fact that the viral RNA-dependent polymerase NS5B has poor replicative fidelity, which causes the frequent emergence of drug-resistant strains, clinical applications of anti-HCV drugs are now mostly limited to combination regimens [10]. Nowadays, DAA-based combinations such as sofosbuvir and daclatasvir, sofosbuvir/ledipasvir, ombitasvir/paritaprevir/ritonavir and dasabuvir, sofosbuvir and velpatasvir, glecaprevir and pibrentasvir, and grazoprevir and elbasvir are the standard of care for HCV patients [11]. Current drug combinations are well tolerated and show high efficacy; however, because of extremely high costs, the access to therapy remains low on a global scale [12]. Thus, there is still a need for the development of new antiviral drugs with higher efficacy and improved tolerability but lower costs and wider availability in order to reach global HCV eradication.

CSFV is a highly infectious viral disease that affects domestic and wild pigs [13,14]. It is a major cause of the most devastating diseases for the pig industry from an economical and sanitary point of view, particularly in several African, Asian, South American, and East European countries, where it is still endemic [15]. There is no approved treatment for CSFV. Upon occasional outbreaks in CSFV-free regions, the euthanization of CSFV-confirmed cases and contact animals is compulsorily employed. Moreover, the pre-emptive slaughter of animals on nearby farms is also practiced, which causes tremendous economic losses in the pig industry [16]. Therefore, there is substantial scope for the development of anti-CSFV drugs, which could be a good control strategy to inhibit viral replication in infected herds and to control major economic loss in case of an outbreak.

HCV belonging to the *Hepacivirus* genus and CSFV from the *Pestivirus* genus in the *Flaviviridae* family show a high degree of homology in genomic organization, replication, and protein function. In the past, because of the lack of an efficient method for HCV propagation, CSFV was frequently used as a surrogate model to study the role of envelope glycoproteins of HCV and to discover new HCV drugs [17,18]. 

Glycosyltransferases (GTs) are involved in the biosynthesis of highly glycosylated glycoproteins found on the surface of many viruses [19,20]. GTs take part in many fundamental biological processes, and the modulation of their activities by efficient inhibitors is a potential means for the control of certain cellular functions. In recent years, intensive research on the design of new effective GT inhibitors has been conducted. The design of the structure of GT inhibitors is generally based on similarity with their natural substrates—donor type and acceptor type—or on their analogies to the components of the transition state. 

In the case of donor-type substrates such as NDP-sugar, the pyrophosphate moiety interacts with a bivalent metal cation present in an enzyme active site. Numerous analogues of the pyrophosphate linker have been proposed [21,22,23,24]. However, such compounds have an anionic character, which prevents their entry into cells through the phospholipid bilayer. The solution to this problem, particularly for in vivo biological applications, may be achieved by the preparation of GT inhibitors containing a neutral diphosphate surrogate, which would interact with bivalent metal cations [25,26]. 

**Scheme 1:**
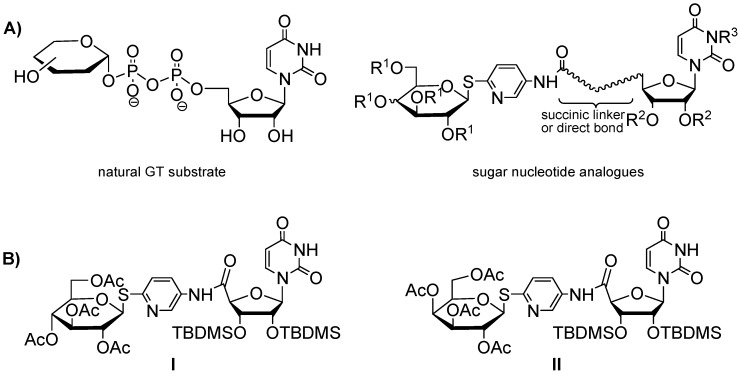
(**A**) Natural glycosyltransferase (GT) substrates and sugar nucleotide analogues. (**B**) The most active derivatives previously described.

Recently, we have reported the identification and mechanism of action of a series of glycoconjugates as anti-CSFV and -HCV compounds [27,28]. These were derivatives of (5-amino-2-pyridyl) 1-thioglycosides and selectively protected uridine, a new kind of sugar nucleotide analogue designed as a potential GT inhibitor. In order to increase their stability under cellular conditions, the anomeric oxygen atom occurring in the natural GT donor-type substrates was replaced by sulfur in these structures. Another change in the structure was the replacement of the pyrophosphate bridge with a pyridyl ring connected to a succinic spacer through an amide bond or with a pyridyl ring connected to the uridine by an amide bond, omitting a succinic spacer. The choice of such a linker was based on the ability to coordinate divalent metal ions (Scheme 1) [26,29,30]. 

We have shown that two of these previously described compounds, designated as **I** and **II**, exerted the most significant inhibitory effects on in vitro CSFV and HCV infections in the series, showing half-maximum inhibitory concentration (IC_50_) (defined as the concentration of a compound that causes a 50% reduction in foci after an immunohistochemical method) values in the low micromolar range (Scheme 1B) [28]. The obtained results showed that the level of synthesis of structural proteins in CSFV- and HCV-infected cells was downregulated upon treatment with these compounds. Although these compounds were synthesized as GT inhibitors, which was experimentally confirmed with the isolated β-1,4-Galactosyltransferase (β1,4-GalT) enzyme for fully deprotected derivatives of compound **I**, they were found to significantly reduce the viral genome replication process by up to 90%.

**Scheme 2:**
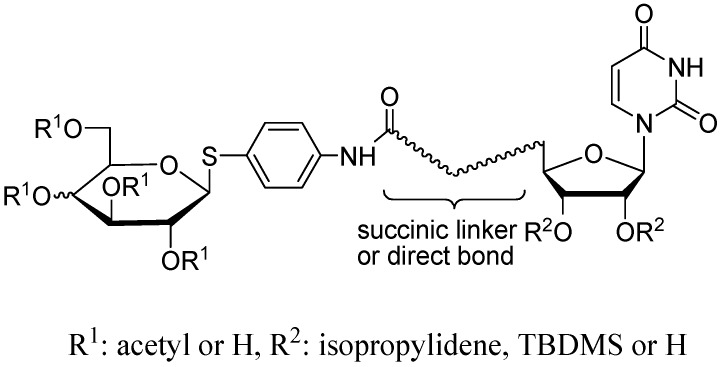
General structure of tested glycoconjugates used in this study.

As a part of our program to study the new anti-CSFV and -HCV compounds, here we describe the design, synthesis, and biological evaluation of other novel thioglycosyl analogues of GT substrates as potential antiviral compounds against these two major economically significant pathogens. To investigate the influence of the presence of the aromatic nitrogen in the spacer on the glycoconjugates’ biological activity, a series of new glycoconjugates (**7**–**15**) were synthesized on the basis of the same structural fragments as in the case of the earlier-described glycoconjugates but replacing the pyridine ring by a benzene ring (Scheme 2). Two promising compounds with novel properties were selected for further development as lead hits, and attempts were made to elucidate their modes of action.

## 2. Results and Discussion 

### 2.1. Chemistry 

Our earlier studies on the antiviral activity of glycoconjugates, in which (5-amino-2-pyridyl) 1-thioglycosides were connected via an amide bond with uridine derivatives with or without a succinic linker, showed that some of them were able to inhibit CSFV and HCV replication in a cell-culture system [28]. Their biological activity depended on the structure of the linker connecting the sugar parts and uridine moiety as well as on the type of protective groups in both parts of the glycoconjugates. In order to check whether the biological activity was influenced by the presence of a free electron pair on the aromatic nitrogen in the linker, we synthesized glycoconjugates, derivatives of 4-aminophenyl 1-thioglycosides, in which the aromatic nitrogen atom was replaced by a carbon atom (Scheme 2). 

The effect of the presence and the type of the protecting groups in both parts of a glycoconjugate, that is, in the sugar ring and in the uridine moiety, was examined. The ribose in the uridine moiety was protected using the isopropylidene group or more hydrophobic *tert*-buthyldimethylsilyl (TBDMS) groups. An acetyl moiety was selected for protection of hydroxyl groups in the sugar ring. Ester-type protecting groups for the sugar part were chosen because of the likelihood of their hydrolysis by enzymes present within the cells. These groups increased the hydrophobicity of the glycoconjugates and allowed them to enter cells. The protecting groups in the uridine part were chosen not only to allow for the regioselective synthesis of glycoconjugates, but also to improve the hydrophobicity of the products and their stability within the cell. It was observed for the previously described glycoconjugates, derivatives of (5-amino-2-pyridyl) 1-thioglycosides (**I** and **II**), that higher antiviral activity was demonstrated for derivatives containing silyl protecting groups in the uridine moiety [28]. To check whether the same influence of protecting groups may be observed for glycoconjugate derivatives of 4-aminophenyl 1-thioglycosides, we synthesized derivatives containing all the above-mentioned types of protection.

The substrates used in condensation reactions were uridine derivatives containing carboxyl groups **1**–**4** and 4-aminophenyl β-d-1-thioglycoside **5** or **6** derivatives of d-glucose and d-galactose, respectively. 

**Scheme 3:**
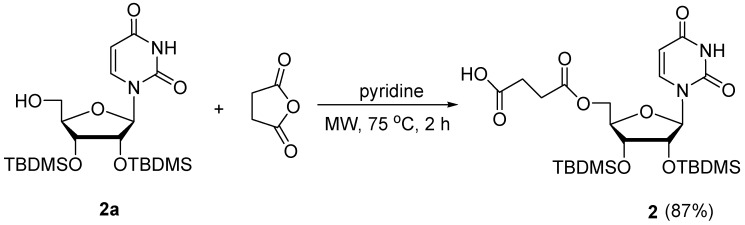
Synthesis of uridine derivative **2**.

The syntheses of uridine derivative **1** [27], 2′,3′-*O*-isopropylideneuridine-5′-carboxylic acid **3 [31]**, and 2′,3′-di-*O*-*tert*-butyldimethylsilyluridine-5′-carboxylic acid **4** [28] were described earlier. Succinic acid mono-2′,3′-di-*O*-*tert*-butyldimethylsilyl-uridin-5′-yl ester **2** was prepared from 2′,3′-di-*O*-*tert*-butyldimethylsilyluridine **2a** [32] by acylation with succinic anhydride carried out in pyridine under microwave irradiation (Scheme 3). The use of microwave irradiation eliminated the need for *N*,*N*-dimethylaminopyridine (DMAP) addition as was required for compound **1** synthesis. This simplified the final purification of uridine derivative **2** and improved its yield. A second group of structural components of glycoconjugates included 4-aminophenyl β-d-1-thioglycosides **5**, **6**. A simple and efficient synthesis of per-*O*-acetylated 4-aminophenyl β-d-1-thioglycosides **5** and **6** by reduction of a nitro group in an aglycon of corresponding 4-nitrophenyl β-d-1-thioglycosides was described recently [33]. 

**Scheme 4:**
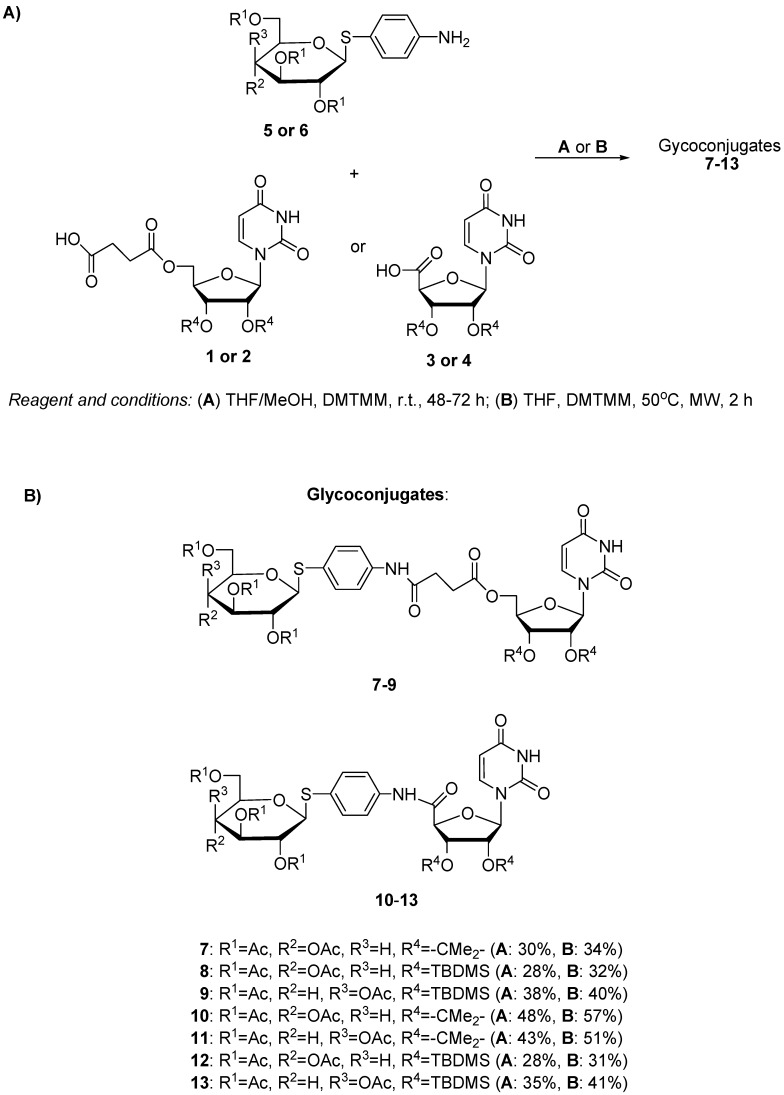
(**A**) Synthesis of glycoconjugates **7**–**13**. (**B**) Structures of compounds **7**–**13**.

Having the structural elements of both glycoconjugates, one of which has a carboxyl group in its structure (compounds **1**–**4**) and the second which has an amino group in aglycon (compounds **5**–**6**), it was possible to join them by amide bond formation. For this purpose, the activation of carboxylic acid was required [34]. The most efficient formation of an amide bond in glycoconjugate derivatives of aryl 1-thioglycosides was observed in the presence of 2-chloro-4,6-disubstituted-1,3,5-triazines and *N*-methylmorpholine, which generates in situ 4-(4,6-dimethoxy-1,3,5-triazin-2-yl)-4-methylmorpholinium chloride (DMTMM) as a condensing agent [27,28]. The long reaction time (up to 2 days) can be shortened by applying microwave irradiation. To prove this thesis, for condensation of the amine group in the sugar derivatives **5**,**6** with the carboxylic group in the uridine derivatives **1**–**4** (Scheme 4), DMTMM was used at room temperature (Table 1, Procedure **A**) or in combination with microwave irradiation (standard program, 50 °C) (Table 1, Procedure **B**). In both condensation procedures, glycoconjugates **7**–**13** were obtained. The application of microwave irradiation allowed for the shortening of the reaction time and increased yields of the products, as shown in Table 1.

**Scheme 5:**
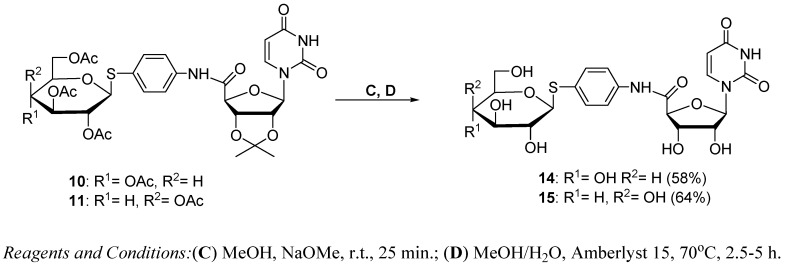
Synthesis of glycoconjugates **14** and **15**.

Compounds **10** and **11** were selected as substrates to provide fully deprotected glycoconjugates (Scheme 5). The complete removal of protecting groups from glycoconjugates **10** and **11** was conducted in two steps. The first step was the methanolysis of acetyl groups in the sugar part by the action of sodium methoxide (NaOMe) in methanol. In the second step, the isopropylidene group was removed. In this step, it was necessary to acidify the reaction mixture to pH 2. The best results were obtained when amberlyst-15 in aqueous methanol solution was employed in the two-step, one-pot deprotection of derivatives **10** and **11**, leading to glycoconjugates **14** (58% yield) and **15** (64% yield), respectively.

All the synthesized compounds were purified by column chromatography, and their structures were determined by NMR and mass spectra. The compounds were examined for their antiviral activities against CSFV and HCV. 

### 2.2. Biological Evaluation

#### 2.2.1. Antiviral Activity against Classical Swine Fever Virus

In the in vitro antiviral screening of the compounds, a cytotoxicity analysis and antiviral assay using CSFV were performed according to the procedures previously reported [35,36]. All measurements were performed with three replicates, and the results are presented in Table 2. In the first set of experiments, the cytotoxicity of the compounds was measured using a MTS-based cell proliferation assay (CellTiter 96 AQueous non-radioactive cell-proliferation assay) in swine kidney (SK6) non-infected cells, and thereafter all the compounds were tested in antiviral screening below their half-maximum cytotoxic concentration (CC_50_) (compound concentration that reduced cell viability by 50%) values. The toxicity analysis demonstrated that the tested compounds produced a dose-dependent toxic effect. Out of the nine compounds tested, compounds **8**, **12**, and **13** were relatively toxic; however the remaining six compounds were well tolerated by the cells (Table 2).

**Figure 1:**
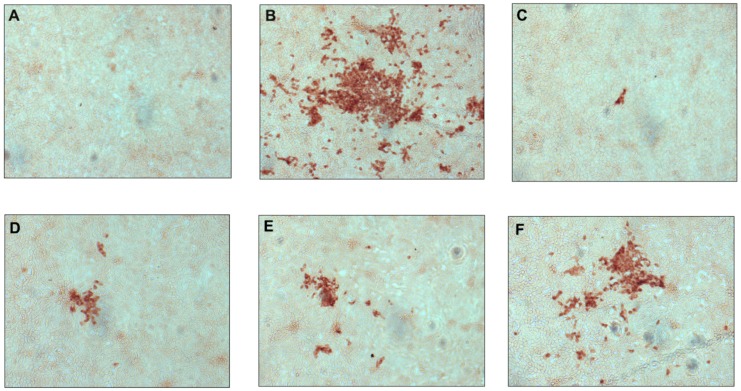
Effect of compound **9** on pseudo-plaque formation in classical swine fever virus (CSFV)-infected swine kidney (SK6) cells. SK6 cells were mock infected (**A**) or infected with CSFV at a MOI of 0.001 (**B**–**F**). At 2 h post infection, cells were treated with various concentrations of compound **9** ((**C**) 16 µM, (**D**) 12 µM, (**E**) 8 µM, and (**F**) 4 µM)) or left untreated (positive control (**B**)). Two days post infection, cells were fixed, and virus pseudo-plaques were detected by immunostaining with rabbit polyclonal anti-E^rns^ serum.

Because of the fact that CSFV does not cause a cytopathic effect, it is not possible to directly observe the foci of viral growth [37]. Therefore, all synthesized compounds were evaluated for anti-CSFV activity by a pseudo-plaque reduction assay, in which the virus propagation measurement was based on visualization of the foci (pseudo-plaques). SK6 cells were infected with a low multiplicity of infection (MOI) of the virus to visualize single pseudo-plaques after an immunoperoxidase monolayer assay (IPMA) for detection of the areas of maximum concentration of viral glycoproteins. The dose-dependent inhibition of CSFV propagation after treatment with active compound **9** as an example is shown in Figure 1. This was exhibited by the reduction in the average size and number of pseudo-plaques compared to the control. 

First, in structure–activity studies, we evaluated the influence of the types of protecting groups in the uridine moiety on the anti-CSFV activity. The results, showing the concentrations required to inhibit CSFV replication by 50% (IC_50_), are summarized in Table 2. Interestingly, among the tested compounds, derivatives **8**, **9**, and **12** appeared to be the most active, while the other compounds were less potent, which suggests that silyl protected derivatives exhibit stronger activity than those containing a uridine part with an isopropylidene protecting group. These compounds reduced CSFV infection with IC_50_ values of 4.5, 4.2, and 4 µM, respectively, and showed safety, with calculated half-maximum concentration (CC_50_) values of 42, 124, and 56 µM. Thus, the selectivity indexes (SIs), defined as the CC_50_/IC_50_ ratio, were 9.3, 29.5 and 14.0, respectively. 

Moreover, further modifications were introduced involving the removal of all protecting groups to determine how their absence affected the antiviral activity. Although completely deprotected compounds **14** and **15** exhibited low cytotoxicity (CC_50_ of 256 and 278 µM), they turned out to be inactive against the virus (IC_50_ of 241 and 257 µM).

Our results showed that the presence and type of protecting groups are very important for anti-CSFV activity. When the isopropylidene protecting group in compounds **7**, **10**, and **11** was replaced by TBDMS groups in compounds **8**, **12**, and **13**, the anti-CSFV activity was significantly increased. We concluded that the presence of hydrophobic protecting groups of the uridine part of glycoconjugates is crucial for anti-CSFV activity. 

We further investigated the effect of a succinic linker on the biological activities of the synthesized compounds (compounds **7**–**9** versus compounds **10**–**13** for which a succinic linker was omitted). In this series, compound **12** showed good activity, with an IC_50_ value of 4 µM, but relatively high cytotoxicity (CC_50_ of 56 µM). In this case, the SI was 14.0. Only slightly worse activity was shown for compound **9**, with an IC_50_ value of 4.2 µM, but in this case the cytotoxicity (CC_50_ of 124 µM) was lower than in the case of compound **12**. It could therefore be concluded that glycoconjugate **9** containing a succinic linker exhibited better antiviral properties than derivative **12** without such a linker, indicating that the introduction of the succinic linker might be beneficial for anti-CSFV activity.

Both types of linker connecting the sugar part with uridine affected the antiviral activity of the tested glycoconjugates similarly. However, derivative **9** containing a succinic acid fragment in the linker structure was significantly less toxic. Despite the fact that both glycoconjugates derived from d-glucose and d-galactose were tested, the rules regarding the influence of the attached sugar moiety on the antiviral activity could not be established. The most important factor was most likely the type of protective groups of 2′-OH and 3′-OH in uridine. The TBDMS groups that increased the hydrophobicity of the glycoconjugates significantly improved their antiviral activity.

#### 2.2.2. Antiviral Activity against Hepatitis C Virus

**Figure 2:**
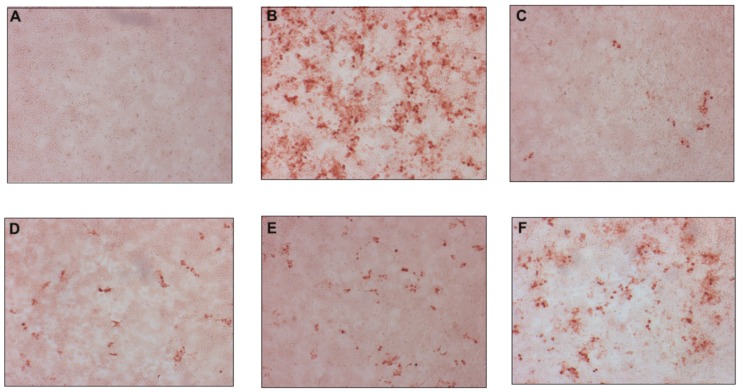
Effect of compound **9** on pseudo-plaque formation in hepatitis C virus (HCV)-infected Huh-7.5 cells. Huh-7.5 cells were mock infected (**A**) or infected with HCV at a MOI of 0.1 (**B**–**F**). At 3 h p.i., the virus was removed, and the cells were treated with various concentrations of compound **9** ((**C**) 10 µM, (**D**) 8 µM, (**E**) 6 µM, and (**F**) 4 µM) or left untreated (positive control (**B**)). Three days post infection, cells were fixed, and HCV pseudo-plaques were detected using anti-HCV core antibody.

To further assess the effect of all the synthesized compounds on HCV infection, a cell-culture-infectious HCV (HCVcc) pseudo-plaque reduction assay, which allowed for the complete replication of HCV, including the in vitro production and secretion of HCVcc (Jc1/JFH genotype 2a) in the human hepatoma Huh-7.5 cell-culture system, was used [38,39]. HCV-infected cells were treated with different, non-toxic concentrations of the compounds and sofosbuvir, an inhibitor of NS5B RNA-dependent RNA polymerase, as a positive control, and an IPMA with an anti-HCV core antibody was performed [40,41]. In parallel, the cell viability of non-infected cells under the same concentrations was determined by a MTT assay in Huh 7.5 cells to calculate CC_50_ values for all the compounds. The results of the IPMA are shown in Figure 2, where a dose-dependent inhibition of viral HCV replication after treatment with compound **9** can be observed. 

The screening results showed that compounds **9** and **12** were the most promising antiviral agents. These derivatives significantly reduced Jc1/JFH 2a HCV propagation, with IC_50_ values of 4.9 and 13.5 µM, and showed safety, as the CC_50_ values were 257 and 270 µM, which led to SIs of 52.4 and 20.0, respectively. The high antiviral activity of both compounds against HCV provided confirmation of the results obtained for CSFV. In the case of compound **8**, active against CSFV, although the activity could be considered satisfactory, it exhibited a rather poor SI of 4 because of the high cytotoxicity for Huh-7.5 cells. Thus, only compounds **9** and **12** were selected for further evaluation. 

#### 2.2.3. Time-of-Drug Addition Studies

**Figure 3:**
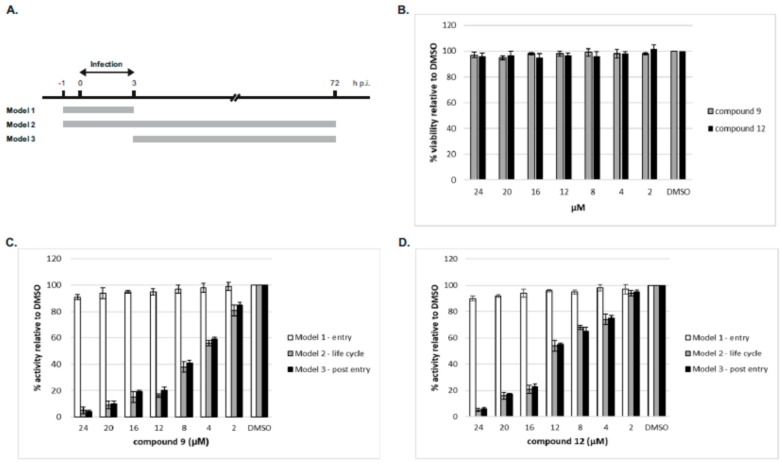
Antiviral activity of compounds **9** and **12** on hepatitis C virus (HCV) infection. (**A**) Schematic representation of infection models. (**B**) The viability analysis of Huh7-J20 cells treated for 72 h with various concentrations of compounds **9** and **12** with no viral infection. The values of compound-treated cells are expressed as percentage relative to DMSO-treated cells expressed as 100% (control). (**C**,**D**) Huh7-J20 cells were pre-treated for 1 h and infected with cell-culture-infectious HCV (HCVcc) in the presence of various doses of **9** (**C**), **12** (**D**), or DMSO as a control for 3 h. Then, the inoculum was removed, and fresh medium without compound was added for 72 h (Model 1, white bars). Huh7-J20 cells were pre-treated for 1 h; infected with HCVcc for 3 h in the presence of various doses of **9** (**C**), **12** (**D**), or DMSO; and then incubated for 72 h with fresh medium including inhibitor or DMSO (Model 2, grey bars). Huh7-J20 cells were infected for 3 h with HCVcc and then treated with various doses of **9** (**C**), **12** (**D**), or DMSO for 72 h (Model 3, black bars). The inhibitory effects were determined by a secreted alkaline phosphatase (SEAP) assay performed on infected cell medium. Errors bars represent the S.D. of the means for three replicates.

Encouraged by the promising results for the antiviral activity of compounds **9** and **12** against HCV, we decided to examine their mechanisms of action more thoroughly. We have previously shown that the mechanism of action of compounds **I** and **II** belonging to pirydyl thioglycosyl analogues of GT donor-type substrates is related to the inhibition of viral replication [28]. To check whether the changes in structures influenced the antiviral activity mechanism, we tested the most active of the new synthesized compounds (**9** and **12**) by three different protocols of infection according to Magri et al. (2016) using human hepatoma cell line Huh7-J20 [42] (Figure 3A). This cell line stably expresses enhanced green fluorescent protein (eGFP) fused in-frame to the secreted alkaline phosphatase (SEAP) via a recognition sequence of the viral NS3/4A serine protease [43]. The level of SEAP activity in the culture medium directly correlates with the level of intracellular viral RNA replication because of the fact that the SEAP reporter is released from the fusion protein after the cleavage by NS3/4A protease produced during viral infection. To test whether the synthesized compounds affected HCV entry, we used model 1 of infection, in which Huh7-J20 cells were incubated for 1 h with different concentrations of tested compounds or DMSO as a control and were infected with HCVcc genotype 2a JFH-1 strain in the presence of these compounds for a further 3 h. After the cells were washed, fresh medium without inhibitors was added for 72 h. In Model 2, which was used to test the effect of the compounds on the full viral life cycle, Huh7-J20 cells were pre-treated for 1 h and infected with the virus for 3 h together with various concentrations of compounds or DMSO; then the inoculum was replaced with fresh medium containing potential drugs for a further 72 h. To investigate the possible effect of the synthesized inhibitors on post-viral entry processes such as RNA replication and/or virus assembly, the cells were infected for 3 h with JFH-1 HCVcc, and then the incubation with fresh medium containing various concentrations of compounds or DMSO was extended for 72 h (Model 3). All of these compounds were used with non-toxic concentrations as determined by the MTT assay (Figure 3B). 

The obtained results indicated that none of the tested compounds targeted virus entry (Model 1). We showed that both compounds affected virus genome replication because they showed strong antiviral activity in both the full life cycle (Model 2) and post-viral entry model (Model 3), as observed by the reduction in the SEAP levels compared to the control (Figure 3C,D). Moreover, in our additional experiments, we confirmed that, as in case of tested compounds **9** and **12**, the positive control drug sofosbuvir, an inhibitor of the HCV NS5B RNA-dependent polymerase, exerts its antiviral activity effect on post-viral entry or the full life cycle model, with no effect on virus entry (Supplementary Figure S31).

#### 2.2.4. The Inhibitory Effect of Compounds **9** and **12** on HCV Replication

To further characterize the anti-HCV activity of compounds **9** and **12**, the most representative compounds in this study, and confirm that they target viral replication, a stable replicon cell line, Huh7-J17, was used [44]. This particular puromycin-resistant cell line, expressing a monocistronic replicon encoding non-structural proteins, a structural core protein, and firefly luciferase as a reporter gene, was chosen to check the ability of the compounds to inhibit HCV replication, as the level of the reporter protein after inhibitory treatment directly correlates with virus RNA replication. Non-toxic amounts of the compounds as tested by the MTT assay were added to the cells, incubation was carried out for 72 h, and the inhibition of viral replication after the inhibitory treatment was determined by measuring the luciferase activity. Cells treated with sofosbuvir were used as the positive control of the experiments. 

**Figure 4:**
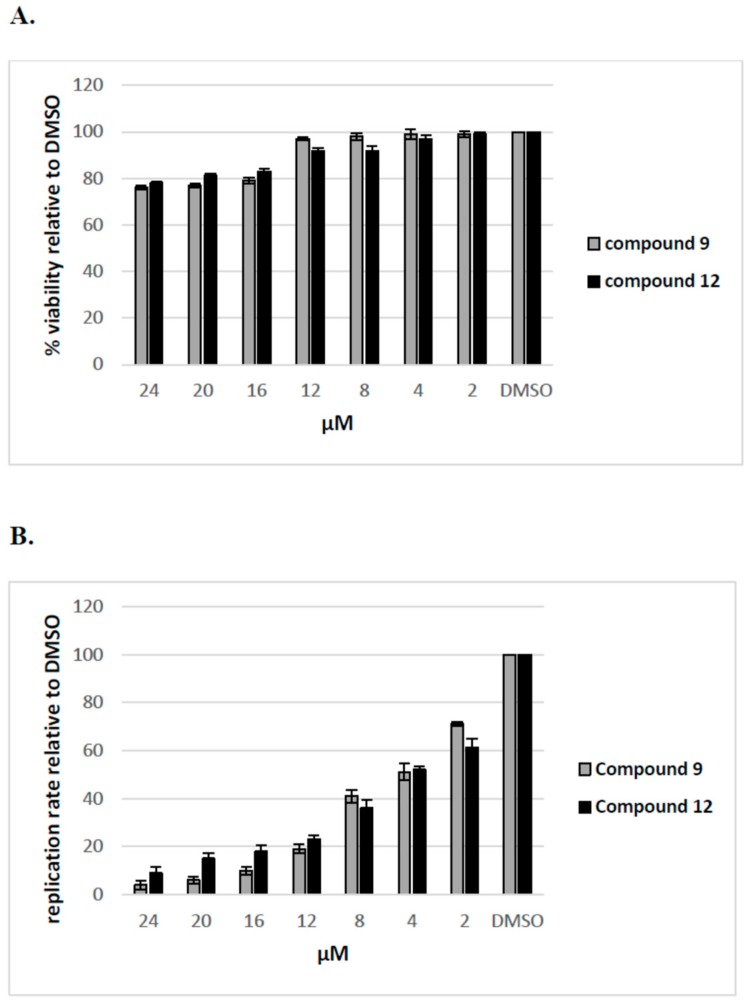
Antiviral effect of compounds **9** and **12** on viral replication. Huh7-J17 cells were plated in the presence of different doses of **9** and **12**, and 72 h later, cell viability was measured by MTT assay (**A**) or cells were lysed and antiviral effect was measured comparing luciferase activity (**B**). The values of compound-treated cells are expressed as percentage relative to DMSO-treated cells defined as 100% (control). Errors bars represent the S.D. of the means for 3 replicates.

Our results showed that the tested compounds significantly inhibited HCV replication, which confirmed the previous observation in the HCVcc system (Figure 4). Both drugs blocked viral RNA replication in a dose-dependent manner, with a nearly 90% reduction after treatment with the highest doses of the compounds. Calculated IC_50_ values for compounds **9** and **12** were 4.18 and 4.24 µM, respectively; CC_50_ values were 133 and 130 µM, respectively. The IC_50_ and CC_50_ values for the positive control—sofosbuvir—were 0.028 and 23.5 µM, respectively. 

**Figure 5:**
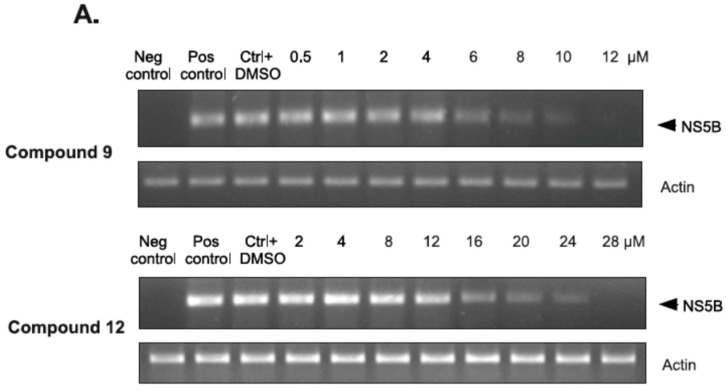

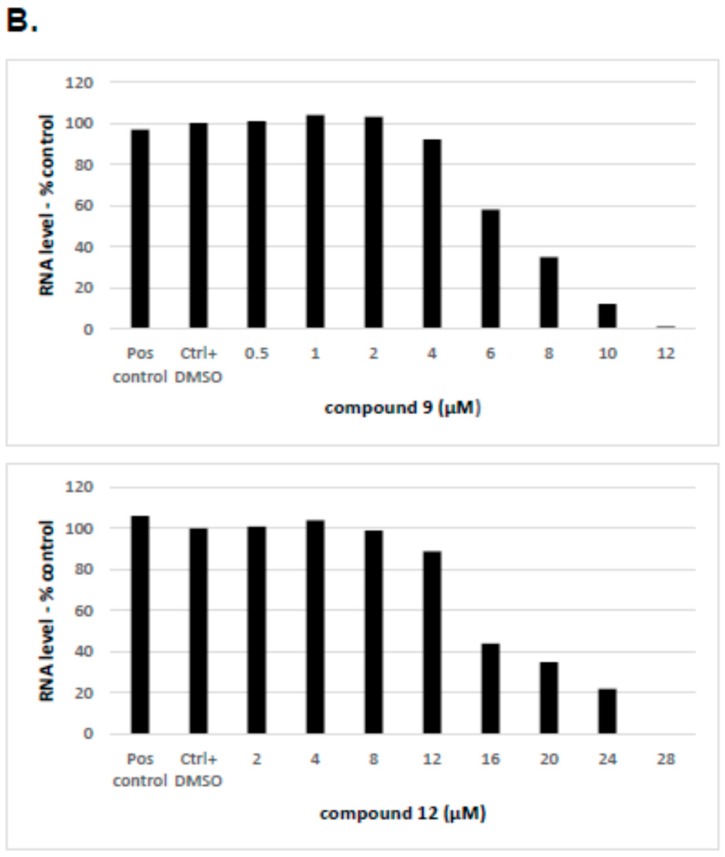
Effect of compounds **9** and **12** on viral RNA synthesis. (**A**) Overnight-grown Huh7.5 cells were infected with hepatitis C virus (HCV) for 4 h and washed with phosphate-buffered saline (PBS), and fresh media with different concentrations of compounds were added. Total RNA was isolated after 48 h and subjected to RT-PCR for HCV NS5B gene to detect the inhibition of viral replication. Actin was amplified simultaneously and was loaded as internal control. (**B**) The bar graphs present the densitometric analysis of NS5B level detected in infected Huh-7 cells treated with different doses of compounds, expressed as percentage of NS5B detected in the control.

To further confirm the influence of the synthesized compounds on viral RNA synthesis, RT-PCR for the NS5B gene was performed on the viral RNA isolated from the Huh 7.5 HCV-infected cells treated with different concentrations of the compounds. Overall, as shown in Figure 5, a significant dose-dependent inhibition of the amount of viral RNA was observed for cells treated with compounds **9** and **12** in comparison to untreated infected cells, which additionally indicated that the synthesized compounds target virus replication. 

#### 2.2.5. Inhibitory Activity against β-1,4GalT

The described glycoconjugates were designed as analogues of GT substrates; therefore, the synthesized derivatives **7**–**15** were subjected to evaluation of their inhibitory activity towards commercially available β-1,4GalT according to the previously described method [45]. As expected, the protected glycoconjugates **7**–**13** did not show the ability to inhibit β-1,4GalT activity. Only fully deprotected derivatives **14** and **15** demonstrated the ability of β-1,4GalT inhibition. Derivative **14** (being the deprotected counterpart of the active antiviral glycoconjugate **12**) was found to be an effective inhibitor of β-1,4GalT, and for this compound, an IC_50_ of 0.33 mM was determined. Glycoconjugate **15** (the deprotected equivalent of compound **11**, which showed negligible antiviral activity at high cytotoxicity) reduced the enzyme activity by only 10% at the maximum test concentration of 0.8 mM. 

As the result of these experiments, a correlation between the demonstrated antiviral activity of the protected glycoconjugates and the ability to inhibit β-1,4GalT through their deprotected counterparts was observed. When the protected glycoconjugate **12** exhibited antiviral activity, its fully deprotected derivative **14** was able to inhibit β-1,4GalT activity. In turn, when the protected glycoconjugate **11** showed no significant antiviral activity, its fully deprotected derivative **15** did not show inhibitory activity against the tested enzyme. The presence of protecting groups in derivatives **11** and **12** likely increased their lipophilicity as a result of them more easily penetrating the cells. Intracellular hydrolytic enzymes are responsible for the removal of protecting groups and, consequently, the formation of a glycoconjugate capable of inhibiting β-1,4GalT. 

## 3. Materials and Methods 

### 3.1. General Experimental Procedures 

All chemicals used in the experiments were of analytical grade and were purchased from Acros Organics, Sigma-Aldrich, or Merck. Column chromatography was performed on Silica Gel 60 (70–230 mesh, Fluka, St. Louis, MI, USA). NMR solvents were purchased from ACROS Organics.

The reactions were monitored by thin layer chromatography (TLC) on precoated plates of silica gel 60 F_254_ (Merck Millipore, Burlington, MA, USA). The TLC plates were inspected under UV light (λ = 254 nm) or by charring the plates after spraying with a 10% ethanolic solution of sulfuric acid. Crude products were purified using column chromatography performed on silica gel using toluene/EtOAc or CHCl_3_/MeOH as solvent systems. Organic solvents were evaporated on a rotary evaporator under reduced pressure at 50 °C. The purity of the tested compounds **7**–**15** was determined using HPLC-MS/MS. All tested compounds were at least 95% pure.

Microwave reactions were carried out in a Discover BenchMate (CEM Corporation, Matthews, NC, USA) microwave equipped with 10 mL vessels using a standard program at 50 °C (max. pressure of 1.5 bar; average power of 20 W). The structures of the products were determined by NMR and mass spectra. NMR spectra were recorded for solutions in CDCl_3_, in DMSO-*d*_6_, or in D_2_O with TMS or DSS as internal standards using Varian spectrometers at frequencies of 300 or 600 MHz and an Agilent spectrometer at a frequency of 400 MHz. Chemical shifts (δ) are expressed in ppm, and coupling constants (*J*) are expressed in hertz. The following abbreviations were used to explain the observed multiplicities: s: singlet; d: doublet; dd: doublet of doublets; ddd: doublet of doublet of doublets; t: triplet; dd ~ t: doublet of doublets resembling a triplet (with similar values of coupling constants); m: multiple; b: broad. High-resolution mass spectra (HRMS) were measured in the positive mode with a Mariner (Perspective Biosystem) detector using the electrospray ionization (ESI) technique. ESI low-resolution mass spectrometry was performed on a 4000 QTrap (Applied Biosystem/MDS Sciex, Foster City, CA, USA) mass spectrometer. Optical rotations were measured with a JASCO P-2000 polarimeter using a sodium lamp (589.3 nm) at room temperature. Melting-point measurements were performed on a Stanford Research Systems OptiMelt (MPA 100). Succinic acid mono-2′,3′-*O*-isopropylidene-uridin-5′yl ester **1** [27], 2′,3′-di-*O*-*tert*-butyldimethylsilyluridine **2a** [32], 2′,3′-*O*-isopropylideneuridine-5′-carboxylic acid **3** [31], 2′,3′-*O*-di-*O*-*tert*-butyldimethylsilyluridine-5′-carboxylic acid **4** [28], 4-aminophenyl 2,3,4,6-tetra-*O*-acetyl-1-thio-β-d-glucopyranoside **5**, 4-aminophenyl 2,3,4,6-tetra-*O*-acetyl-1-thio-β-d-galactopyranoside **6** [33], and DMTMM [46] were prepared according to the respective published procedures. 

#### 3.1.1. Synthesis of Succinic Acid Mono-2′,3′-di-*O*-*tert*-butyldimethylsilyl-uridin-5′-yl Ester (**2**)

To a solution of 2′,3′-di-*O*-*tert*-butyldimethylsilyluridine **2a** (0.550 g, 1.16 mmol) in dry pyridine (2 mL), the succinic anhydride (0.128 g, 1.28 mmol) was added. The resulting mixture was microwaved in a reactor set for 2 h at 75 °C. The reaction progress was monitored on TLC in a CHCl_3_/MeOH (10:1) solvent system. Then the reaction mixture was concentrated with toluene (3 × 10 mL) in order to remove the whole amount of pyridine. The residue was purified on a column packed with silica gel using a toluene/AcOEt solvent system (gradient of 2:1 to 1:2). Product **2** was a white solid (0.578 g, 87%): m.p. of 163–165 °C; ${\left[\alpha \right]}_{D}^{25}$ 19.5 (c 1.3, MeOH). ^1^H-NMR (400 MHz, DMSO-*d*_6_): δ 0.01, 0.03, 0.07 (3s, 12H, CH_3_Si), 0.82, 0.87 (2s, 18H, (CH_3_)_3_CSi), 2.42–2.63 (m, 4H, CH_2_), 4.05 (m, 1H, H-4′_ur_), 4.10–4.17 (m, 2H, H-5′a_ur_, H-5′b_ur_), 4.26–4.33 (m, 2H, H-2′_ur_, H-3′_ur_), 5.69 (dd, 1H, *J* = 1.9 Hz, *J* = 8.0 Hz, H-5_ur_), 5.75 (d, 1H, *J* = 5.5 Hz, H-1′_ur_), 7.66 (d, 1H, *J* = 8.0 Hz, H-6_ur_), 8.29 (s, 1H, NH), 11.36 (s, 1H, NH), 12.24 (bs, 1H, COOH). ^13^C-NMR (100 MHz, DMSO-*d*_6_): δ −5.13, −5.01, −4.77, −4.63 (CH_3_Si), 17.58, 17.68 ((CH_3_)_3_CSi), 25.58, 25.66 ((CH_3_)_3_CSi), 28.58, 28.63 (CH_2_CO), 63.19 (C-5′_ur_), 71.40, 73.72 (C-3′_ur_, C-2′_ur_), 81.74 (C-4′_ur_), 87.75 (C-1′_ur_), 102.11 (C-5_ur_), 140.26 (C-6_ur_), 150.62 (C-2_ur_), 162.94 (C-4_ur_), 171.88 (CO), 173.26 (COOH). HRMS (ESI) (*m/z*) [M + Na]^+^ calcd for C_25_H_44_N_2_O_9_Si_2_Na, 595.2483; found, 595.2491.

#### 3.1.2. Synthesis of Glycoconjugates **7**–**13**

**Procedure A.** The appropriate amine, **5** or **6**, (0.25 mmol) and uridine derivative (**1**–**4**) (0.25 mmol) were dissolved in dry THF (5 mL) with the addition of MeOH (0.3 mL). To the obtained solution, DMTMM (0.07 g, 0.25 mmol) and *N*-methylmorpholine (0.016 mL, 0.12 mmol) were added. The mixture was stirred at room temperature for 48 to 72 h (appropriate reaction times are given in Table 1). The reaction progress was monitored on TLC in two alternative eluents—CHCl_3_/MeOH (10:1) or toluene/AcOEt (1:1). After completion, the reaction mixtures were concentrated, dissolved in CH_2_Cl_2_ (30 mL), washed twice with brine (5 mL), and dried with anhydrous MgSO_4_; the adsorbent was filtered off, and the filtrate was concentrated to give crude products **7**–**13**, which were purified directly by column chromatography with an appropriate solvent system as indicated.

**Procedure B.** The appropriate amine, **5** or **6**, (0.25 mmol) and uridine derivative (**1**–**4**) (0.25 mmol) were dissolved in dry THF (5 mL). DMTMM (0.07 g, 0.25 mmol) and *N*-methylmorpholine (0.016 mL, 0.12 mmol) were added to this mixture. The resulting mixture was microwaved in a reactor set for 2 h at 50 °C. The reaction progress was monitored on TLC in the eluents mentioned above. Then the solvent was evaporated, and further work-up was the same as in case of **Procedure A**. 

##### Glycoconjugate **7**

Starting from amine **5** (0.113 g) and uridine derivative **1** (0.096 g), glycoconjugate **7** was obtained by **Procedure A** and **Procedure B** as a white solid after column chromatography (toluene/AcOEt; gradient of 10:1 to 1:2). The yield was as follows: **Procedure A** (0.062 g, 30%) and **Procedure B** (0.070 g, 34%): m.p. of 129–132 °C; ${\left[\alpha \right]}_{D}^{25}$ −15.5 (c 1.0, CHCl_3_). ^1^H-NMR (400 MHz, CDCl_3_): δ 1.31, 1.55 (2s, 6H, (CH_3_)_2_C), 1.99, 2.02, 2.05, 2.09 (4s, 12H, CH_3_CO), 2.62–2.79 (m, 4H, 2 × CH_2_), 3.71 (ddd, 1H, *J* = 2.6 Hz, *J* = 4.7 Hz, *J* = 9.8 Hz, H-5_glu_), 4.16 (dd, 1H, *J* = 2.6 Hz, *J* = 12.2 Hz, H-6a_glu_), 4.26 (dd, 1H, *J* = 4.7 Hz, *J* = 12.2 Hz, H-6b_glu_), 4.28–4.55 (m, 3H, H-4′_ur_, H-5′a_ur_, H-5′b_ur_), 4.63 (d, 1H, *J* = 10.0 Hz, H-1_glu_), 4.85 (dd, 1H, *J* = 3.4 Hz, *J* = 6.3 Hz, H-3′_ur_), 4.93 (dd ~ t, 1H, *J* = 9.5 Hz, *J* = 9.8 Hz, H-4_glu_), 4.98–5.07 (m, 2H, H-2′_ur_, H-2_glu_), 5.22 (dd ~ t, 1H, *J* = 9.3 Hz, *J* = 9.5 Hz, H-3_glu_), 5.57 (d, 1H, *J* = 1.7 Hz, H-1′_ur_), 5.72 (d, 1H, *J* = 8.1 Hz, H-5_ur_), 7.28 1 (d, 1H, *J* = 8.1 Hz, H-6_ur_), 7.40–7.54 (m, 4H, H*-*Ph), 7.91 (s, 1H, NH), 9.05 (s, 1H, NH). ^13^C-NMR (100 MHz, CDCl_3_): δ 20.58, 20.60, 20.79 (CH_3_CO), 25.24, 27.15 ((CH_3_)_2_C), 29.40 (CH_2_CONH), 32.01 (CH_2_COO), 62.13 (C-6_glu_), 64.05 (C-5′_ur_), 68.22 (C-2_glu_), 69.99 (C-4_glu_), 74.02 (C-3_glu_), 75.78 (C-5_glu_), 80.74 (C-3′_ur_), 84.38 (C-2′_ur_), 85.22 (C-4′_ur_), 85.80 (C-1_glu_), 95.23 (C-1′_ur_), 102.49 (C-5_ur_), 114.65 ((CH_3_)_2_C), 119.94, 125.30, 128.23, 129.03, 134.91, 138.59 (C-Ph), 143.59 (C-6_ur_), 149.80 (C-2_ur_), 163.22 (C-4_ur_), 169.30, 169.43, 169.80, 170.19, 170.71, 172.58 (CO). HRMS (ESI) (*m*/*z*): [M + Na]^+^ calcd for C_36_H_43_N_3_O_17_SNa, 844.2205; found, 844.2219.

##### Glycoconjugate **8**

Starting from amine **5** (0.113 g) and uridine derivative **2** (0.143 g), glycoconjugate **8** was obtained by **Procedure A** and **Procedure B** as a white solid after column chromatography (toluene/AcOEt; gradient of 20:1 to 2:1). The yield was as follows: **Procedure A** (0.071 g, 28%) and **Procedure B** (0.081 g, 32%): m.p. of 116–119 °C; ${\left[\alpha \right]}_{D}^{25}$ 27.5 (c 0.9, CHCl_3_). ^1^H-NMR (400 MHz, CDCl_3_): δ 0.05, 0.08, 0.10, 0.13 (4s, 12H, CH_3_Si), 0.89, 0.91 (2s, 18H, (CH_3_)_3_CSi), 1.98, 2.01, 2.08, 2.09 (4s, 12H, CH_3_CO), 2.62–2.84 (m, 4H, 2 × CH_2_), 3.72 (ddd, 1H, *J* = 2.5 Hz, *J* = 4.6 Hz, *J* = 9.8 Hz, H-5_glu_), 4.00–4.08 (m, 2H, H-3′_ur_, H-6b_glu_), 4.16 (dd, 1H, *J* = 2.5 Hz, *J* = 12.2 Hz, H-6a_glu_), 4.17–4.32 (m, 3H, H-2′_ur_, H-4_ur_, H-5′b_ur_), 4.49 (dd, 1H, *J* = 3.9 Hz, *J* = 13.9 Hz, H-5′a_ur_), 4.64 (d, 1H, *J* = 10.0 Hz, H-1_glu_), 4.93 (dd ~ t, 1H, *J* = 9.3 Hz, *J* = 9.8 Hz, H-4_glu_), 5.02 (dd ~ t, 1H, *J* = 9.6 Hz, *J* = 10.0 Hz, H-2_glu_), 5.22 (dd ~ t, 1H, *J* = 9.4 Hz, *J* = 9.6 Hz, H-3_glu_), 5.64 (d, 1H, *J* = 3.0 Hz, H-1′_ur_), 5.77 (d, 1H, *J* = 8.1 Hz, H-5_ur_), 7.42–7.50 (m, 4H, H_Ph_), 7.63 (s, 1H, NH), 7.65 (d, 1H, *J* = 8.1 Hz, H-6_ur_), 8.48 (s, 1H, NH). ^13^C-NMR (100 MHz, CDCl_3_): δ −5.02, −4.81, −4.50, −4.26 (CH_3_Si), 18.01, 18.04 ((CH_3_)_3_C), 20.58, 20.60, 20.68 (CH_3_CO), 25.78, 25.79 ((CH_3_)_3_CSi), 29.19 (CH_2_CONH), 31.85 (CH_2_COO), 62.11 (C-3′_ur_), 63.04 (C-5′_ur_), 68.20 (C-2_glu_), 69.98 (C-4_glu_), 70.93 (C-6_glu_), 73.99 (C-3_glu_), 75.17 (C-2′_ur_), 75.80 (C-5_glu_), 81.09 (C-4′_ur_), 85.91 (C-1_glu_), 91.12 (C-1′_ur_), 102.10 (C-5_ur_), 119.93, 126.14, 134.80, 138.33 (C_Ph_), 140.24 (C-6_ur_), 149.87 (C-2_ur_), 162.89 (C-4_ur_), 169.27, 169.38, 170.18, 170.61, 172.42, (CO). HRMS (ESI) (*m*/*z*): [M + Na]^+^ calcd for C_45_H_67_N_3_O_17_SSi_2_Na, 1132.3627; found, 1132.3622.

##### Glycoconjugate **9**

Starting from amine **6** (0.113 g) and uridine derivative **2** (0.143 g), glycoconjugate **9** was obtained by **Procedure A** and **Procedure B** as a white solid after column chromatography (toluene/AcOEt; gradient of 20:1 to 1:1). The yield was as follows: **Procedure A** (0.096 g, 38%) and **Procedure B** (0.101 g, 40%): m.p. of 106–109 °C; ${\left[\alpha \right]}_{D}^{25}$ 9.2 (c 1.0, CHCl_3_). ^1^H-NMR (300 MHz, CDCl_3_): δ 0.05, 0.08, 0.10, 0.14 (4s, 12H, CH_3_Si), 0.89, 0.91 (2s, 18H, (CH_3_)_3_CSi), 1.98, 2.04, 2.1, 2.12 (4s, 12H, CH_3_CO), 2.62–2.84 (m, 4H, 2 × CH_2_), 3.94 (m, 1H, H-5_gal_), 4.04 (dd, 1H, *J* = 4.4 Hz, *J* = 5.9 Hz, H-3′_ur_), 4.11 (dd, 1H, *J* = 6.3 Hz, *J* = 11.2 Hz, H-6a_gal_), 4.18 (dd, 1H, *J* = 6.9 Hz, *J* = 11.2 Hz, H-6b_gal_), 4.22–4.31 (m, 3H, H-2′_ur_, H-3′_ur_, H-5′b_ur_), 4.46 (dd, 1H, *J* = 3.9 Hz, *J* = 13.6 Hz, H-5′a_ur_), 4.66 (d, 1H, *J* = 9.8 Hz, H-1_gal_), 5.06 (dd, 1H, *J* = 3.4 Hz, *J* = 10.0 Hz, H-3_gal_), 5.21 (dd ~ t, 1H, *J* = 9.8 Hz, *J* = 10.0 Hz, H-2_gal_), 5.41 (d, 1H, *J* = 3.4 Hz, H-4_gal_), 5.63 (d, 1H, *J* = 2.9 Hz, H-1′_ur_), 5.78 (dd, 1H, *J* = 1.7 Hz, *J* = 8.2 Hz, H-5_ur_), 7.42–7.53 (m, 4H, H_Ph_), 7.67 (d, 1H, *J* = 8.2 Hz, H-6_ur_), 7.83 (s, 1H, NH), 9.26 (s, 1H, NH). ^13^C-NMR (75 MHz, CDCl_3_): δ −5.03, −4.82, −4.50, −4.26 (CH_3_Si), 18.01, 18.04 ((CH_3_)_3_C), 20.59, 20.67, 20.70, 20.87 (CH_3_CO), 25.78, 25.79 ((CH_3_)_3_CSi), 29.21 (CH_2_CONH), 31.88 (CH_2_COO), 62.55 (C-3′_ur_), 63.00 (C-5′_ur_), 67.25, 67.35 (C-2_gal_, C-4_gal_), 70.90 (C-6_gal_), 71.99, 74.39 (C-3_gal_, C-5_gal_), 75.16 (C-2′_ur_), 81.07 (C-4′_ur_), 86.99 (C-1_gal_), 91.21 (C-1′_ur_), 102.05 (C-5_ur_), 119.97, 127.24, 134.12, 138.12 (C_Ph_), 140.34 (C-6_ur_), 149.89 (C-2_ur_), 163.11 (C-4_ur_), 169.45, 170.05, 170.21, 170.40, 172.44 (CO). HRMS (ESI) (*m*/*z*): [M + Na]^+^ calcd for C_45_H_67_N_3_O_17_SSi_2_Na, 1132.3627; found, 1132.3629.

##### Glycoconjugate **10**

Starting from amine **5** (0.113 g) and uridine derivative **3** (0.075 g), glycoconjugate **10** was obtained by **Procedure A** and **Procedure B** as a white solid after column chromatography (toluene/AcOEt with gradient of 10:1 to 1:1; then CHCl_3_/MeOH with gradient of 100:1 to 10:1). The yield was as follows: **Procedure A** (0.088 g, 48%) and **Procedure B** (0.105 g, 57%): m.p. of 149–153 °C; ${\left[\alpha \right]}_{D}^{25}$ −44.7 (c 1.0, CHCl_3_). ^1^H-NMR (400 MHz, CDCl_3_): δ 1.37, 1.59 (2s, 6H, (CH_3_)_2_C), 1.98, 2.01, 2.08, 2.09 (4s, 12H, CH_3_CO), 3.68 (ddd, 1H, *J* = 2.7 Hz, *J* = 4.6 Hz, *J* = 9.9 Hz, H-5_glu_), 4.13–4.24 (m, 2H, H-6a_glu_, H-6b_glu_), 4.62 (d, 1H, *J* = 10.1 Hz, H-1_glu_), 4.71 (d, 1H, *J* = 2.5 Hz, H-4′_ur_), 4.91 (dd ~ t, 1H, *J* = 9.4 Hz, *J* = 9.9 Hz, H-4_glu_), 5.04 (dd ~ t, 1H, *J* = 9.3 Hz, *J* = 10.1 Hz, H-2_glu_), 5.21(dd, 1H, *J* = 9.3 Hz, *J* = 9.4 Hz, H-3_glu_), 5.24 (dd, 1H, *J* = 2.5 Hz, *J* = 6.4 Hz, H-3′_ur_), 5.29 (dd, 1H, *J* = 2.2 Hz, *J* = 6.4 Hz, H-2′_ur_), 5.47 (d, 1H, *J* = 2.2 Hz, H-1′_ur_), 5.79 (d, 1H, *J* = 8.0 Hz, H-5_ur_), 7.25 (d, 1H, *J* = 8.0 Hz, H-6_ur_), 7.42–7.52 (m, 4H, H_Ph_), 8.46 (s, 1H, NH), 8.68 (s, 1H, NH). ^13^C-NMR (100 MHz, CDCl_3_): δ 20.59, 20.79 (CH_3_CO), 24.96, 26.92 ((CH_3_)_2_C), 62.17 (C-6_glu_), 68.29, 69.85 (C-2_glu_, C-4_glu_), 74.02 (C-3_glu_), 75.81 (C-5_glu_), 82.60 (C-2′_ur_), 83.67 (C-3′_ur_), 85.61 (C-1_glu_), 87.48 (C-4′_ur_), 99.35 (C-1′_ur_), 103.32 (C-5_ur_), 114.54 ((CH_3_)_2_C), 119.91, 126.06, 134.98, 137.88 123.85 (C_Ph_), 143.79 (C-6_ur_), 150.26 (C-2_ur_), 162.23 (C-4_ur_), 167.18, 169.30, 169.39, 170.19, 170.74 (CO). HRMS (ESI) (*m*/*z*): [M + Na]^+^ calcd for C_32_H_37_N_3_O_15_SNa, 758.1838; found, 759.1844.

##### Glycoconjugate **11**

Starting from amine **6** (0.113 g) and uridine derivative **1** (0.075 g), glycoconjugate **11** was obtained by **Procedure A** and **Procedure B** as a white solid after column chromatography (toluene/AcOEt with gradient of 10:1 to 1:1; then CHCl_3_/MeOH with gradient of 100:1 to 20:1). The yield was as follows: **Procedure A** (0.079 g, 43%) and **Procedure B** (0.094 g, 51%): m.p. of 158–160 °C; ${\left[\alpha \right]}_{D}^{25}$ −36.7 (c 1.0, CHCl_3_). ^1^H-NMR (400 MHz, CDCl_3_): δ 1.37, 1.59 (2s, 6H, (CH_3_)_2_C), 1.97, 2.05, 2.10, 2.11 (4s, 12H, CH_3_CO), 3.91 (m, 1H, H-5_gal_), 4.10 (dd, 1H, *J* = 6.3 Hz, *J* = 11.3 Hz, H-6a_gal_), 4.16 (dd, 1H, *J* = 7.0 Hz, *J* = 11.3 Hz, H-6b_gal_), 4.62 (d, 1H, *J* = 10.2 Hz, H-1_gal_), 4.70 (d, 1H, *J* = 2.2 Hz, H-4′_ur_), 5.04 (dd, 1H, *J* = 3.2 Hz, *J* = 9.8 Hz, H-3_gal_), 5.19 (dd ~ t, 1H, *J* = 9.8 Hz, *J* = 10.2 Hz, H-2_gal_), 5.23 (dd, 1H, *J* = 2.2 Hz, *J* = 6.6 Hz, H-3′_ur_), 5.27 (dd, 1H, *J* = 2.3 Hz, *J* = 6.6 Hz, H-2′_ur_), 5.40 (dd, 1H, *J* = 0.8 Hz, *J* = 3.2 Hz, H-4_gal_), 5.46 (d, 1H, *J* = 2.3 Hz, H-1′_ur_), 5.79 (d, 1H, *J* = 7.8 Hz, H-5_ur_), 7.24 (d, 1H, *J* = 7.8 Hz, H-6_ur_), 7.44–7.54 (m, 4H, H_Ph_), 8.43 (s, 1H, NH), 8.53 (s, 1H, NH). ^13^C-NMR (100 MHz, CDCl_3_): δ 20.58, 20.66, 20.73, 20.87 (CH_3_CO), 24.97, 26.94 ((CH_3_)_2_C), 61.52 (C-6_gal_), 67.20, 67.26 (C-4_gal_, C-2_gal_), 72.02 (C-3_gal_), 74.34 (C-5_gal_), 82.58 (C-2′_ur_), 83.62 (C-1_gal_), 86.67 (C-3′ur), 87.46 (C-4′_ur_), 99.43 (C-1′_ur_), 103.27 (C-5_ur_), 114.60 ((CH_3_)_2_C), 119.93, 127.20, 128.22, 129.03, 134.38, 137.60 (C_Ph_), 143.82 (C-6_ur_), 150.19 (C-2_ur_), 162.11 (C-4_ur_), 167.10, 169.43, 170.06, 170.25, 170.56 (CO). HRMS (ESI) (*m*/*z*): [M + Na]^+^ calcd for C_32_H_37_N_3_O_15_SNa, 758.1838; found, 759.1847.

##### Glycoconjugate **12**

Starting from amine **5** (0.113 g) and uridine derivative **1** (0.122 g), glycoconjugate **12** was obtained by **Procedure A** and **Procedure B** as a thick syrup after column chromatography (toluene/AcOEt; gradient of 10:1 to 1:1). The yield was as follows: **Procedure A** (0.072 g, 31%) and **Procedure B** (0.081 g, 35%): ${\left[\alpha \right]}_{D}^{25}$ −54.3 (c 1.0, CHCl_3_). ^1^H-NMR (400 MHz, CDCl_3_): δ −0.05, 0.04, 0.15, 0.24 (4s, 12H, CH_3_Si), 0.84, 0.96 (2s, 18H, (CH_3_)_3_CSi), 1.98, 2.01, 2.09, 2.10 (4s, 12H, CH_3_CO), 3.67 (ddd, 1H, *J* = 2.8 Hz, *J* = 4.6 Hz, *J* = 10.0 Hz, H-5_glu_), 4.17 (dd, 1H, *J* = 2.2 Hz, *J* = 12.4 Hz, H-6a_glu_), 4.25 (dd, 1H, *J* = 4.6 Hz, *J* = 12.4 Hz, H-6b_glu_), 4.33 (d, 1H, *J* = 4.5 Hz, H-3′_ur_), 4.52 (s, 1H, H-4′_ur_), 4.62 (d, 1H, *J* = 10.0 Hz, H-1_glu_), 4.88 (dd, 1H, *J* = 4.5 Hz, *J* = 8.2 Hz, H-2′_ur_), 4.92 (dd ~ t, 1H, *J* = 9.4 Hz, *J* = 10.0 Hz, H-4_glu_), 5.02 (dd ~ t, 1H, *J* = 9.4 Hz, *J* = 10.0 Hz, H-2_glu_), 5.21 (dd, 1H, *J* = 9.4 Hz, *J* = 9.4 Hz, H-3_glu_), 5.26 (d, 1H, *J* = 8.2 Hz, H-1′_ur_), 5.81 (d, 1H, *J* = 8.0 Hz, H-5_ur_), 7.24 (d, 1H, *J* = 8.0 Hz, H-6_ur_), 7.45–7.50 (m, 2H, H_Ph_), 7.68–7.75 (m, 2H, H_Ph_), 8.27 (s, 1H, NH), 9.79 (s, 1H, NH). ^13^C-NMR (100 MHz, CDCl_3_): δ −5.24, −4.74, −4.61, −4.43 (CH_3_Si), 17.85, 18.03 ((CH_3_)_3_C), 20.58, 20.60, 20.79 (CH_3_CO), 25.70, 25.81 ((CH_3_)_3_CSi), 62.05 (C-6_glu_), 68.18 (C-4_glu_), 69.55, 69.93 (C-2_glu_, C-2′_ur_), 74.03 (C-3_glu_), 75.01 (C-3′_ur_), 75.83 (C-5_glu_), 85.80, 86.34 (C-1_glu_, C-4′_ur_), 97.03 (C-1′_ur_), 103.01 (C-5_ur_), 120.06, 125.92, 134.95, 138.40 (C_Ph_), 145.43 (C-6_ur_), 150.53 (C-2_ur_), 161.84 (C-4_ur_), 167.36, 169.23, 169.34, 170.18, 170.63 (CO). HRMS (ESI) (*m*/*z*): [M + Na]^+^ calcd for C_41_H_61_N_3_O_15_SSi_2_Na, 946.3260; found, 946.3297.

##### Glycoconjugate **13**

Starting from amine **6** (0.113 g) and uridine derivative **1** (0.122 g), glycoconjugate **13** was obtained by **Procedure A** and **Procedure B** as a white solid after column chromatography (toluene/AcOEt; gradient of 20:1 to 4:1). The yield was as follows: **Procedure A** (0.081 g, 35%) and **Procedure B** (0.095 g, 41%): m.p. of 115–118 °C; ${\left[\alpha \right]}_{D}^{25}$ −40.0 (c 0.9, CHCl_3_). ^1^H-NMR (300 MHz, CDCl_3_): δ −0.06, 0.04, 0.15,0.24 (4s, 12H, CH_3_Si), 0.84, 0.96 (2s, 18H, (CH_3_)_3_CSi), 1.96, 2.06, 2.11, 2.12 (4s, 12H, CH_3_CO), 3.69 (m, 1H, H-5_gal_), 4.12 (dd, 1H, *J* = 6.3 Hz, *J* = 11.4 Hz, H-6a_gal_), 4.18 (dd, 1H, *J* = 6.8 Hz, *J* = 11.4 Hz, H-6b_gal_), 4.33 (d, 1H, *J* = 4.4 Hz, H-3′_ur_), 4.52 (s, 1H, H-4′_ur_), 4.64 (d, 1H, *J* = 10.0 Hz, H-1_gal_), 4.89 (dd, 1H, *J* = 4.4 Hz, *J* = 8.1 Hz, H-2′_ur_), 5.05 (dd, 1H, *J* = 3.2 Hz, *J* = 10.0 Hz, H-3_gal_), 5.19 (dd ~ t, 1H, *J* = 10.0 Hz, *J* = 10.0 Hz, H-2_gal_), 5.27 (d, 1H, *J* = 8.3 Hz, H-1′_ur_), 5.41 (d, 1H, *J* = 3.2 Hz, H-4_gal_), 5.84 (dd, 1H, *J* = 1.7 Hz, *J* = 8.1 Hz, H-5_ur_), 7.28 (d, 1H, *J* = 8.1 Hz, H-6_ur_), 7.48–7.52 (m, 2H, H_Ph_), 7.71–7.74 (m, 2H, H_Ph_), 9.09 (s, 1H, NH), 9.84 (s, 1H, NH). ^13^C-NMR (75MHz, CDCl_3_): δ −5.26, −4.74, −4.60, −4.44 (CH_3_Si), 17.86, 18.03 ((CH_3_)_3_C), 20.59, 20.66, 20.71, 20.87 (CH_3_CO), 25.69, 25.81 ((CH_3_)_3_CSi), 61.49 (C-6_gal_), 67.20, 69.53 (C-4_gal_, C-2_gal_), 72.05 (C-2′_ur_), 74.26, 74.41 (C-3_gal_, C-5_gal_), 75.01 (C-3′_ur_), 86.33, 86.89 (C-1_gal_, C-4′_ur_), 97.05 (C-1′_ur_), 103.01 (C-5_ur_), 120.036, 126.95, 134.32, 136.18, 138.17 (C_Ph_), 145.45 (C-6_ur_), 150.53 (C-2_ur_), 161.81 (C-4_ur_), 167.32, 169.37, 170.05, 170.19 (CO). HRMS (ESI) (*m*/*z*): [M + Na]^+^ calcd for C_41_H_61_N_3_O_15_SSi_2_Na, 946.3260; found, 946.3265.

#### 3.1.3. Synthesis of Glycoconjugates **14**,**15**

General two-step procedure**:** Glycoconjugates **10** or **11** (100 mg, 0.140 mmol) were dissolved in MeOH (5.0 mL); then a 1 M solution of NaOMe (100 µL, 0.100 mmol) was added. The reaction solution was mixed for 25 min, and then H_2_O (5.0 mL) and amberlyst 15 were added until reaching pH 2. The reaction was continued for 2.5–5 h at 70 °C. The reaction progress was monitored on TLC in a CHCl_3_/MeOH (1:1) solvent system. After completion, the reaction mixture was filtered, neutralized with aqueous ammonia solution, concentrated in vacuo with silica gel, and purified by column chromatography with an appropriate solvent system as indicated to give product **14** or **15**. 

##### Glycoconjugate **14**

Starting from glycoconjugate **10**, the reaction in the presence of amberlyst 15 was completed after 2.5 h. Glycoconjugate **14** was obtained after column chromatography (CHCl_3_/MeOH with gradient of 7:1 to 1:1 and then MeOH alone) as a white solid (0.043 g, 58%): ${\left[\alpha \right]}_{D}^{25}$ −23.8 (c 0.8, H_2_O). ^1^H-NMR (400 MHz, D_2_O): δ 3.35 (dd ~ t, 1H, *J* = 9.4 Hz, *J* = 10.2 Hz, H-2_glu_), 3.42 (dd ~ t, 1H, *J* = 9.0 Hz, *J* = 9.4 Hz, H-3_glu_), 3.48 (m, 1H, H-5_glu_), 3.53 (dd ~ t, 1H, *J* = 9.0 Hz, *J* = 9.2 Hz, H-4_glu_), 3.72 (dd, 1H, *J* = 5.5 Hz, *J* = 12.5 Hz, H-6a_glu_), 3.90 (d, 1H, *J* = 12.5 Hz, H-6b_glu_), 4.47 (dd ~ t, 1H, *J* = 3.9 Hz, *J* = 4.7 Hz, H-3′_ur_), 4.53 (dd ~ t, 1H, *J* = 4.7 Hz, *J* = 5.1 Hz, H-2′_ur_), 4.61 (d, 1H, *J* = 3.9 Hz, H-4′_ur_), 4.72 (d, 1H, *J* = 10.2 Hz, H-1_glu_), 5.82–5.87 (m, 2H, H-1′_ur_, H-5_ur_), 7.43–7.56 (m, 4H, H_Ph_), 8.02 (d, 1H, *J* = 7.8 Hz, H-6_ur_). ^13^C-NMR (100 MHz, D_2_O): δ 63.55 (C-6_glu_), 72.09, 74.43, 75.11, 75.47 (C-2_glu_, C-3_glu_, C-4_glu_, C-5_glu_), 79.94, 82.60, 85.98, 90.09 (C-2′_ur_, C-3′_ur_, C-4′_ur_, C-1_glu_), 94.50 (C-1′_ur_), 105.06 (C-5_ur_), 124.42, 131.29, 135.39, 138.95 (C_Ph_), 145.99 (C-6_ur_), 154.24 (C-2_ur_), 162.73 (C-4_ur_), 172.32 (CO). HRMS (ESI) (*m/z*): [M + Na]^+^ calcd for C_21_H_25_N_3_O_11_SNa, 550.1107; found, 550.1107.

##### Glycoconjugate **15**

Starting from glycoconjugate **11**, the reaction in the presence of amberlyst **15** was completed after 5 h. Glycoconjugate **15** was obtained after column chromatography (CHCl_3_/MeOH with gradient of 7:1 to 1:1 and then MeOH alone) as a white solid (0.047 g, 64%): ${\left[\alpha \right]}_{D}^{25}$ −33.8 (c 0.5, H_2_O). ^1^H-NMR (400 MHz, D_2_O): δ 3.62 (dd ~ t, 1H, *J* = 9.6 Hz, *J* = 9.8 Hz, H-2_gal_), 3.69 (dd, 1H, *J* = 3.3 Hz, *J* = 9.6 Hz, H-3_gal_), 3.70–3.83 (m, 3H, H-5_gal_, H-6a_gal_, H-6b_gal_), 3.99 (d, 1H, *J* = 3.3 Hz, H-4_gal_), 4.44 23 (dd ~ t, 1H, *J* = 4.3 Hz, *J* = 4.7 Hz, H-3′_ur_), 4.54 (dd ~ t, 1H, *J* = 4.7 Hz, *J* = 5.5 Hz, H-2′_ur_), 4.58 (d, 1H, *J* = 4.3 Hz, H-4′_ur_), 4.72 (d, 1H, *J* = 9.8 Hz, H-1_gal_), 5.78 (d, 1H, *J* = 5.5 Hz, H-1′_ur_), 5.79 (d, 1H, *J* = 7.8 Hz, H-5_ur_), 7.41–7.53 (m, 4H, H_Ph_), 7.94 (d, 1H, *J* = 7.8 Hz, H-6_ur_). ^13^C-NMR (100 MHz, CDCl_3_): δ 63.71 (C-6_gal_), 71.42, 71.94, 74.88, 75.51 (C-4_gal_, C-3_gal_, C-2_gal_, C-5_gal_), 76.69, 81.67, 85.99 (C-2′_ur_, C-3′_ur_, C-4′_ur_), 90.95 (C-1_gal_), 94.77 (C-1′_ur_), 105.07 (C-5_ur_), 124.31, 131.94, 134.78, 138.76 (C_Ph_), 145.87 (C-6_ur_), 155.71 (C-2_ur_), 170.87 (C-4_ur_), 172.21 (CO). HRMS (ESI) (*m*/*z*): [M + Na]^+^ calcd for C_21_H_25_N_3_O_11_SNa, 550.1107; found, 550.1107.

### 3.2. Antiviral Activity

#### 3.2.1. Antiviral Compounds

The synthesized compounds were dissolved in DMSO and stored at −20 °C until future use. Sofosbuvir was purchased from Selleckchem (Munich, Germany).

#### 3.2.2. Cells and Viruses

SK6 cells were grown in Eagle’s Minimum Essential Medium (E-MEM) (Sigma-Aldrich, St. Louis, MI, USA), containing 8% fetal bovine serum (FBS) (Sigma-Aldrich), 100 U/mL penicillin, and 100 µg/mL streptomycin (Invitrogen, Carlsbad, CA, USA) at 37 °C under 5% CO_2_. Human hepatoma cells Huh-7.5 were cultured in Dulbecco’s Modified Eagle Medium (DMEM) (Sigma-Aldrich) containing 10% FBS, 0.5 mM GlutaMax (Invitrogen), 100 U/mL penicillin, and 100 μg/mL streptomycin at 37 °C under 5% humidified CO_2_. Huh7-J20, stably transformed with a SEAP reporter system [43], and the replicon Huh7-J17, which stably express viral RNA [44] (kindly provided by Dr. Arvind Patel (MRC, University of Glasgow Centre for Virus Research, University of Glasgow, Glasgow, UK)), were cultured in the media as Huh-7.5 cells in the presence of puromycin (2 µg/mL) and nonessential amino acids (0.5 mL/50 mL).

CSFV Cellpest strain [47] was obtained from the National Veterinary Institute in Pulawy, Poland. CSFV was grown in monolayers of SK6 cells in 75 cm^2^ tissue culture flasks for 96 h. Viral titers were determined, and stocks were stored at −70 °C before use. 

HCVcc was generated as described previously [38,39]. Briefly, a pFK-Jc1 plasmid containing a full-length chimeric clone of HCV genotype 2a, kindly provided by Dr. Ralf Bartenschlager (University of Heidelberg, Heidelberg, Germany), was linearized by Mlu I, and a pUC-JFH-1/AM7+1 plasmid, kindly provided by Dr. A. Patel, was linearized by Xba I. The plasmids were purified using the Clean-up kit (Qiagen, Hombrechtikon, Switzerland) and were used as the template for transcription with the TranscriptAid T7 High Yield Transcription Kit (Thermo Fischer Scientific, Waltham, MA, USA). In vitro transcribed genomic Jc1/JFH RNA purified using the RNeasy Mini Kit (Qiagen) was used for electroporation of overnight-grown Huh-7.5 cells (10 × 10^6^). HCVcc was obtained by harvesting the culture supernatants 72 h post electroporation, filtering through a 0.45 μm filer, and aliquoting for storage at −80 °C for further use. The Tissue Culture Infectious Dose 50 (TCID_50_) was determined by the Hierholzer & Killington method [48] using the plaque reduction assay described below. 

#### 3.2.3. Cell Viability Assays

SK6 cell viability was determined by the CellTiter 96 AQueous non-radioactive cell-proliferation assay (MTS) (Promega, Madison, WI, USA) described previously [35]. The cytotoxicity of the compounds on Huh-7.5, Huh7-J20, and Huh7-J17 cells was assessed with the MTT method using the standard protocol [49]. The CC_50_ value was determined as the compound concentration required to reduce the cell viability by 50% using CalcuSyn software (Biosoft) from a dose–response curve. 

#### 3.2.4. CSFV Pseudo-Plaque Reduction Assay

Antiviral activity was evaluated by a pseudo-plaque reduction assay by the method described previously [35]. Briefly, confluent monolayers of SK6 cells in 12-well plates were inoculated with CSFV for 1 h at 37 °C. After removal of the virus, the cells were washed with serum-free medium and fresh medium containing inhibitors at different concentrations. Two days post infection, the cells were washed with phosphate-buffered saline (PBS), fixed with 40% acetone in 0.5 × PBS, and dried, and the virus pseudo-plaques were detected by an IPMA with rabbit polyclonal serum anti-E^rns^ diluted to 1:800 in PBS containing 1% Tween 20 and 5% FBS, followed by anti-rabbit horseradish peroxidase (HRP)-conjugated secondary antibody (Santa-Cruz Biotechnology, Dallas, TX, USA) (diluted to 1:1000 in PBS containing 1% Tween 20 and 5% FBS). CSFV pseudo-plaques were visualized using H_2_O_2_/3-amino-9-ethylcarbazole (AEC) and counted. The IC_50_ value was determined as the concentration at which the number of pseudo-plaques (foci) were reduced by 50% compared to untreated infected control cells using GraphPad Prism software. 

#### 3.2.5. The HCVcc Pseudo-Plaque Reduction Assay 

Overnight, Huh-7.5 cells (1.5 × 10^4^ cells/well) seeded in a 96-well plate were inoculated for 3 h with HCVcc containing a supernatant at a MOI of 0.1. Next, the virus was removed, and the cells were overlaid with fresh medium with different concentrations of compounds or sofosbuvir. Three days post infection, the cells were washed with PBS, fixed with methanol for 30 min, and permeabilized in 0.5% Triton X100 in PBS for 5 min followed by another wash with PBS, and immunostaining (IPMA) to detect pseudo-plaques was performed. An anti-core antibody (Hep C cAg (C7-50); Santa Cruz Biotechnology, Dallas, TX, USA; 1:300 dilution) was used as the primary antibody, and anti-mouse HRP labeled antibody (1:1000 dilution) was used as the secondary antibody. HCV-positive pseudo-plaques were detected using the Vector Nova Red kit (Vector Laboratories Ltd., Peterborough, UK), and IC_50_ was calculated as the concentration at which the number of pseudo-plaques (foci) was reduced by 50% compared to infected control cells using the GraphPad Prism software. 

#### 3.2.6. SEAP Reporter Assay

Overnight, Huh7-J20 reporter cells grown in a 96-well tissue culture plate were used to check the antiviral activity of the compounds using three different models of infection according to Magri et al. (2016) [42]. Huh7-J20 cells were pre-treated with different concentrations of the compounds for 1 h and were then infected in the presence of the compounds for 3 h. Next, the virus was removed, and fresh medium without compounds was added for 72 h (Model 1). In Model 2, all steps were the same as in Model 1; however after viral infection, fresh medium together with different concentrations of the compounds was added for 72 h. In Model 3, the various concentrations of the compounds were added only 3 h post infection for 72 h. The antiviral activity was determined by measuring the SEAP activity using the Phospha-Light kit (Applied Biosystems, Foster City, CA, USA) according to the manufacturer’s instructions with some modifications. In brief, 50 µL of culture supernatant was mixed together with 50 µL of assay buffer and incubated for 7 min at room temperature. Next, 50 µL of freshly prepared chemiluminescent substrate was added and incubated in the dark for 45 min, and the SEAP level was measured using a luminometer. 

#### 3.2.7. Antiviral Screening Using Replicon Huh7-J17 Cell Line

Huh7-J17, which stably expresses viral RNA, was plated in 96-well plates in the presence of different concentrations of the compounds, DMSO, or sobosbuvir for 72 h. The inhibition of viral RNA replication was measured by the luciferase activity in lysed cells using the Bright-Glo Luciferase Assay system (Promega, Madison, WI, USA) according to the manufacturer’s protocol. The IC_50_ values were determined using the GraphPad Prism software. 

#### 3.2.8. RNA Inhibition (RT-PCR)

Huh-7.5 cells in 12-well plates (0.1 × 10^6^ cells per well) were infected with HCV at a MOI of 1 and were grown for 48 h in the presence or absence of various concentrations of the compounds or DMSO. Total RNA was purified using Tri Reagent (MRC, Cincinnati, OH, USA) following the manufacturer’s instructions and was dissolved in DNAse/RNAse-free water. Total RNA (1 μg) was used for cDNA synthesis with NS5B gene specific reverse primer with MuLV-RT (Thermo Fisher Scientific, Waltham, MA, USA). PCR with HCV NS5B gene specific primers was performed using cDNA and RUN-Taq polymerase (A&A Biotechnology, Gdynia, Poland). The samples were initially denaturated at 94 °C for 2 min, followed by 30 cycles at 94, 62, and 72 °C for 30 s each, and a final extension step of 2 min at 72 °C. For amplification of a 213 base pairs sequence of the HCV NS5B gene, the forward primer 5′ACA TCA AGT CCG TGT GGA AGG-3′ and reverse primer 5′GCT CCC ATT ACC GCC TGA GGA AGC3′ were used. RT-PCR for actin as an internal control using the forward primer 5′-GCG GGA AAT CGT GCG TGA CAT T-3′ and reverse primer 5′-GAT GGA GTT GAA GGT AGT TTC GTG-3′ was also performed. The PCR products were resolved on 2% agarose gel, and images were analyzed using BioRad Quantity One software. 

#### 3.2.9. Bovine Milk β-1,4GalT I Assay

β-1,4GalT activity was assayed using UDP-Gal as the glycosyl donor and esculine as the glycosyl acceptor. Assays were performed in a total volume of 200 μL. The reaction mixtures contained reagents in the following final concentrations: 50 mM Hepes buffer (pH 5.4), 10 mM MnCl_2_, 2.0 mg/mL Bovine Serum Albumine (BSA), 200 μM esculine, 40 μM UDP-Gal, 10 µL MeOH, and potential inhibitors **7**–**15** at a 0.8 mM concentration. The enzymatic reactions were initiated by the addition of 0.8 mU β4GalT and incubated at 30 °C for 60 min. Inactivation was quickly done by placing the reaction solutions for 3 min in a thermoblock set to 90 °C. The solutions were diluted with water (300 μL), centrifuged for 20 min, and filtered through an M.E. Cellulose filter (0.2 µm × 13 mm), and the filtrate was injected into the RP-HPLC system. The percentage of inhibition was evaluated from the fluorescence intensity of the peaks attributed to the product. The assays were carried out in the linear range of dependence of the product’s peak area from its quantity. For the enzyme-inhibiting compound **14**, the IC_50_ value was designated using the above procedure. The enzymatic reaction mixtures contained the inhibitor in the final concentration range of 0.1–0.8 mM. 

## 4. Conclusions

Previously, we have reported on the identification of glycoconjugate derivatives of (5-amino-2-pyridyl) 1-thioglycosides and selectively protected uridine, a new kind of sugar nucleotide analogue for anti-CSFV and -HCV compounds [22]. We have shown that two compounds from this series (**I** and **II**) exhibited significant antiviral activity against CSFV and HCV and proved that the mechanism of action of these compounds is related to the inhibition of viral replication. 

In this study, a novel type of aryl 1-thioglycosyl analogue of GT substrates, in which the aromatic nitrogen atom was replaced by a carbon atom, were designed and synthesized, and their antiviral activities against CSFV and HCV were examined. In these compounds, the amino group in a glycon of 4-aminophenyl 1-thioglycoside **5** or **6** formed an amide bond with a succinic spacer attached to the selectively protected uridine in the 5′-OH position (compounds **1** or **2**) or directly with a 5′-carboxyl group in the oxidized uridine derivative **3** or **4**. Out of the nine compounds tested, two of them (**9** and **12**) were found to significantly inhibit CSFV propagation in the cell-culture system, with SIs of 29.5 and 14 (Table 2). These compounds also showed high antiviral activity against HCV, with IC_50_ values of 4.9 and 13.5 µM and SIs of 52.4 and 20.0, respectively. It should be noted that although both compounds were less potent than sofosbuvir, they were certainly less toxic. Their CC_50_ values were at least 8 times higher than for sofosbuvir. In the HCV model, we showed that neither compound affected viral entry, but they efficiently targeted the replication process. Additionally, using the Huh7-J17 HCV replicon cell line, we confirmed the influence of compounds **9** and **12** on viral genome replication, as was shown for the previously described compounds **I** and **II**.

Comparing the results obtained during the anti-CSFV activity test for the previously described compounds **I** and **II** (IC_50_ of 3 and 6 µM and CC_50_ of 86 and 151 µM, respectively) with those obtained for glycoconjugates **12** and **13**, structural analogues of **I** and **II** (IC_50_ of 4 and 25 µM and CC_50_ of 56 and 49 µM, respectively), it can be concluded that substitution of the aromatic nitrogen atom in the linker by a carbon atom adversely affects the biological activity and toxicity of the latter compounds. In general, these compounds were slightly less active and more toxic than the previously synthesized glycoconjugates **I** and **II**. The same relationship was observed for the anti-HCV activity of both types of glycoconjugates (IC_50_ values for **I** and **II** were 7 µM for both compounds and SIs were 19.3 and 24.7, respectively, whilst IC_50_ values for **12** and **13** were 13.5 and 7.4 µM and SIs were 20.0 and 1.9, respectively). The introduction of the succinic linker in compounds **7**–**9** increased their antiviral activity in relation to the earlier-discussed glycoconjugates **12** and **13**; thus it can be hypothesized that in the absence of an aromatic nitrogen in the linker, the succinic linker is a moiety responsible for higher antiviral activity. This was particularly noticeable for derivatives **8** and **9**, in which hydroxyl groups in the uridine fragment were protected with TBDMS groups (IC_50_ of 4.5 and 4.2 µM, respectively, for CSFV-infected cells). It should be noted that in the case of the anti-HCV activity studies, compound **9** containing the succinic fragment in the linker was the most active of all the so far tested glycoconjugates (IC_50_ of 4.9 µM) and at the same time had the highest SI of 52.4. 

On the basis of the described observations, we suggest that the key factors responsible for antiviral activity are related to the introduction of an element capable of coordinating metal ions into the spacer connecting the sugar part and uridine moiety (a nitrogen atom in an aromatic ring or a succinic linker) as well as the presence of protective groups that increase glycoconjugate hydrophobicity. 

Further optimization studies aiming at improving the antiviral activity using new compounds in which the aromatic ring in the glycoconjugate linker is replaced by a heteroaromatic system containing more than one nitrogen atom (e.g., triazole system) are currently in progress.

## Figures and Tables

**Table 1:** Yields of glycoconjugates **7**–**13**.

Entry	Substrate 1 Amine	Substrate 2 Uridine Deriv.	Product	Procedure	Reaction Time (h)	Yield (%)
1	**5**	**1**	**7**	**A**	48	30
2	**5**	**1**	**7**	**B**	2	34
3	**5**	**2**	**8**	**A**	48	28
4	**5**	**2**	**8**	**B**	2	32
5	**6**	**2**	**9**	**A**	48	38
6	**6**	**2**	**9**	**B**	2	40
7	**5**	**3**	**10**	**A**	72	48
8	**5**	**3**	**10**	**B**	2	57
9	**6**	**3**	**11**	**A**	72	43
10	**6**	**3**	**11**	**B**	2	51
11	**5**	**4**	**12**	**A**	72	28
12	**5**	**4**	**12**	**B**	2	31
13	**6**	**4**	**13**	**A**	72	35
14	**6**	**4**	**13**	**B**	2	41

**Table 2:** Inhibitory effects of all synthesized compounds on classical swine fever virus (CSFV) and hepatitis C virus (HCV) replication in swine kidney (SK6) and Huh-7.5 cells.

Compound	CSFV (SK6 Cells)			HCV (Huh-7.5 Cells)		
	CC_50_ (μM) ^a^	IC_50_ (μM) ^b^	SI ^c^	CC_50_ (μM) ^a^	IC_50_ (μM) ^b^	SI ^c^
**7**	103 ± 5.9	97 ± 2	1.1	399 ± 11.4	281 ± 4.7	1.4
**8**	42 ± 1.9	4.5 ± 0.3	9.3	16 ± 1.1	4 ± 0.2	4.0
**9**	124 ± 6.8	4.2 ± 0.5	29.5	257 ± 5.7	4.9 ± 0.3	52.4
**10**	326 ± 11.2	149 ± 6.1	2.2	258 ± 6.2	154 ± 2.8	1.7
**11**	340 ± 9.1	217 ± 3.9	1.6	272 ± 4.5	167 ± 3.1	1.6
**12**	56 ± 4.3	4 ± 0.1	14.0	270 ± 2.9	13.5 ± 0.7	20.0
**13**	49 ± 3.5	25 ± 0.9	2.0	14 ± 0.9	7.4 ± 0.2	1.9
**14**	265 ± 8.6	241 ± 9.2	1.1	>475 ± 12.5	444 ± 12.4	>1.1
**15**	278 ± 4.3	257 ± 6.9	1.1	460 ± 9.3	454 ± 9.8	1.0
**I**	86 ± 2.4	3 ± 0.1	28.7	135 ± 2.4	7 ± 0.7	19.3
**II**	151 ± 3.1	6 ± 0.4	25.2	173 ± 3.2	7 ± 0.4	24.7
**SOFOSBUVIR**	ND	ND	ND	31 ± 1.2	0.26 ± 0.02	119.2

^a^ Concentration required to reduce cell viability by 50%. ^b^ Concentration required to reduce virus plaque formation by 50%. Expressed as the mean ± S.D. of three independent experiments. ^c^ In vitro selectivity index (CC_50_/IC_50_). ND: not determined.

## Supplementary Materials

Supplementary materials are available online: Figures S1–S30: ^1^H and ^13^C-NMR spectra of compounds **1**–**15**; Figure S31: Antiviral activity of sofosbuvir on HCV infection.
