# Supplementary material for: Novel Uridine Glycoconjugates, Derivatives of 4-Aminophenyl 1-Thioglycosides, as Potential Antiviral Compounds
Source: Molecules. 2018 Jun 13;23(6):1435. doi: 10.3390/molecules23061435 (PMC6100568; doi:10.3390/molecules23061435)
Supplement: Supplementary file 1 [file molecules-23-01435-s001.pdf]

Supplementary information

Novel uridine glycoconjugates, derivatives of 4-aminophenyl 1-thioglycosides, as potential antiviral compounds

Ewelina Krol[1*], Gabriela Pastuch-Gawolek[2,3*], Binay Chaubey[1,4], Gabriela Brzuska[1], Karol Erfurt[5], Boguslaw Szewczyk[1]

[1]Department of Recombinant Vaccines, Intercollegiate Faculty of Biotechnology, University of Gdansk and Medical University of Gdansk, Abrahama 58, 80-307 Gdansk, Poland

[2]Silesian University of Technology, Faculty of Chemistry, Chair of Organic Chemistry, Bioorganic Chemistry and Biotechnology, Krzywoustego 4, 44-100 Gliwice, Poland

[3]Biotechnology Center, Silesian University of Technology, Krzywoustego 8, 44-100 Gliwice, Poland

[4]Functional Genomics Lab., Centre for Advanced Study, Department of Botany, University of Calcutta, 35, Ballygunge Circular Road, 700019 Kolkata, India

[5]Silesian University of Technology, Faculty of Chemistry, Department of Chemical Organic Technology and Petrochemistry, Krzywoustego 4, 44-100 Gliwice, Poland

* ewelina@biotech.ug.gda.pl; gabriela.pastuch@polsl.pl

1. Spectra

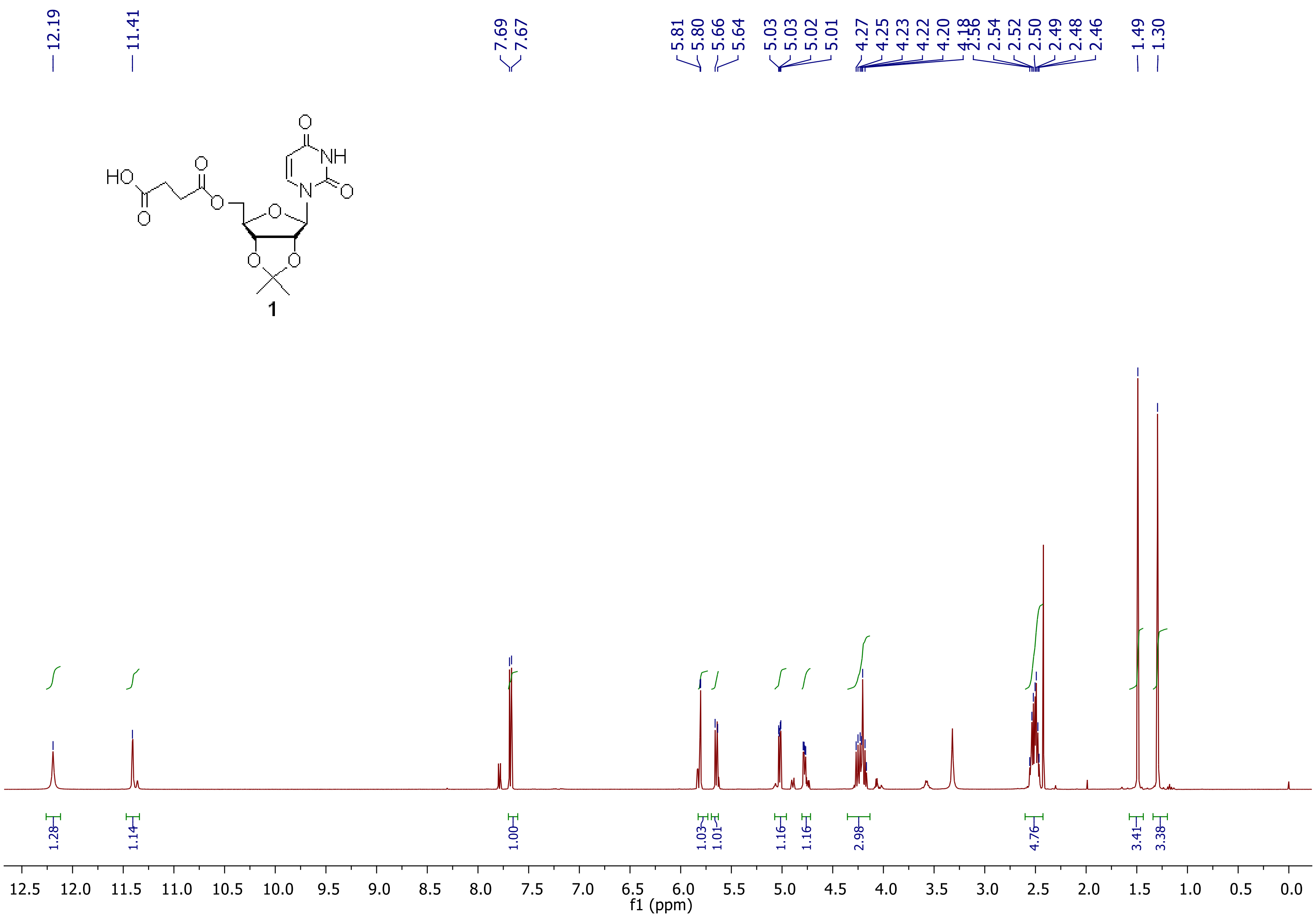

Fig. S1: ^{1}H NMR spectrum of succinic acid mono-2’,3’-*O*-isopropylidene-uridin-5’yl ester **1**.

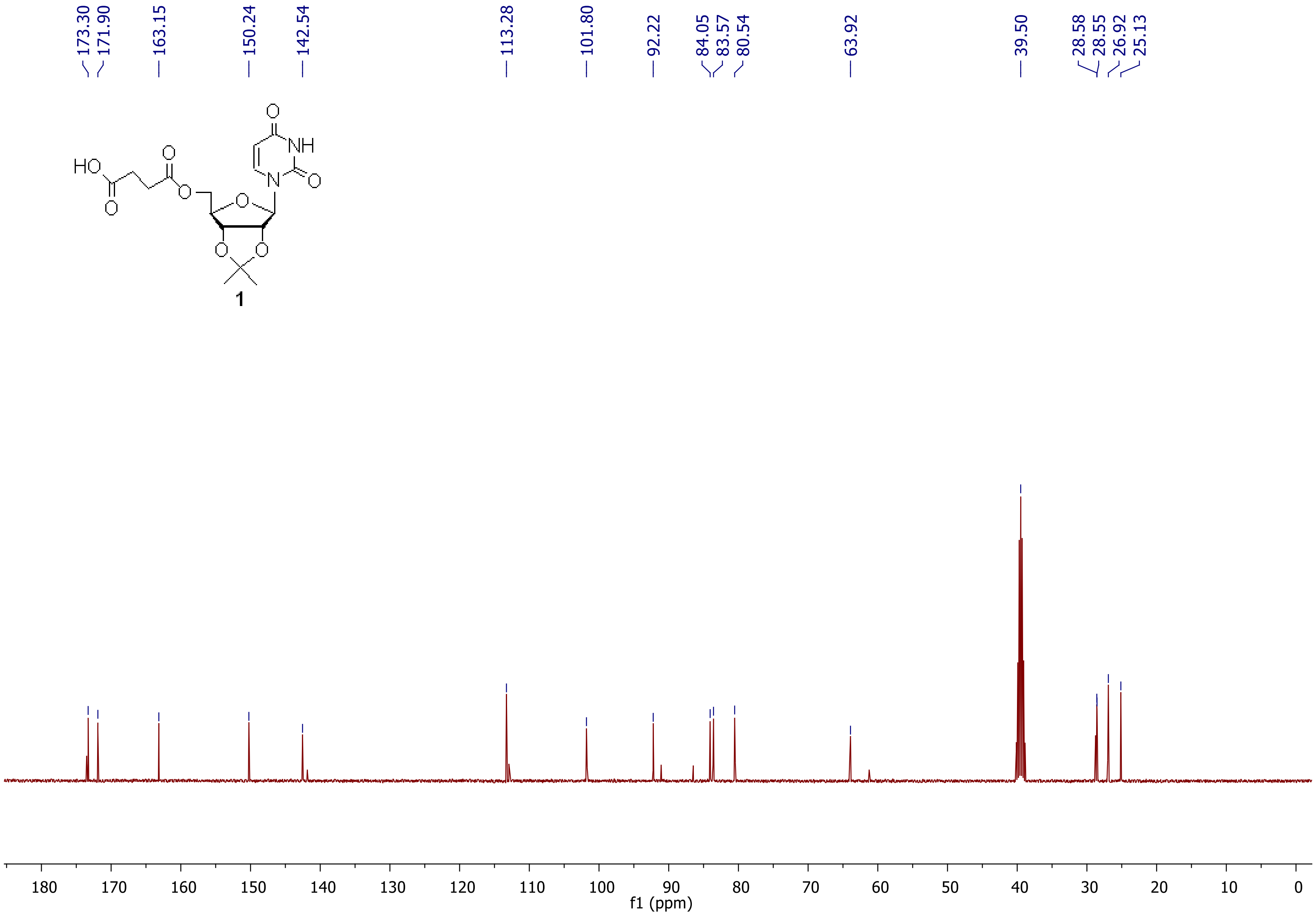

Fig. S2: ^{13}C NMR spectrum of succinic acid mono-2’,3’-*O*-isopropylidene-uridin-5’yl ester **1**.

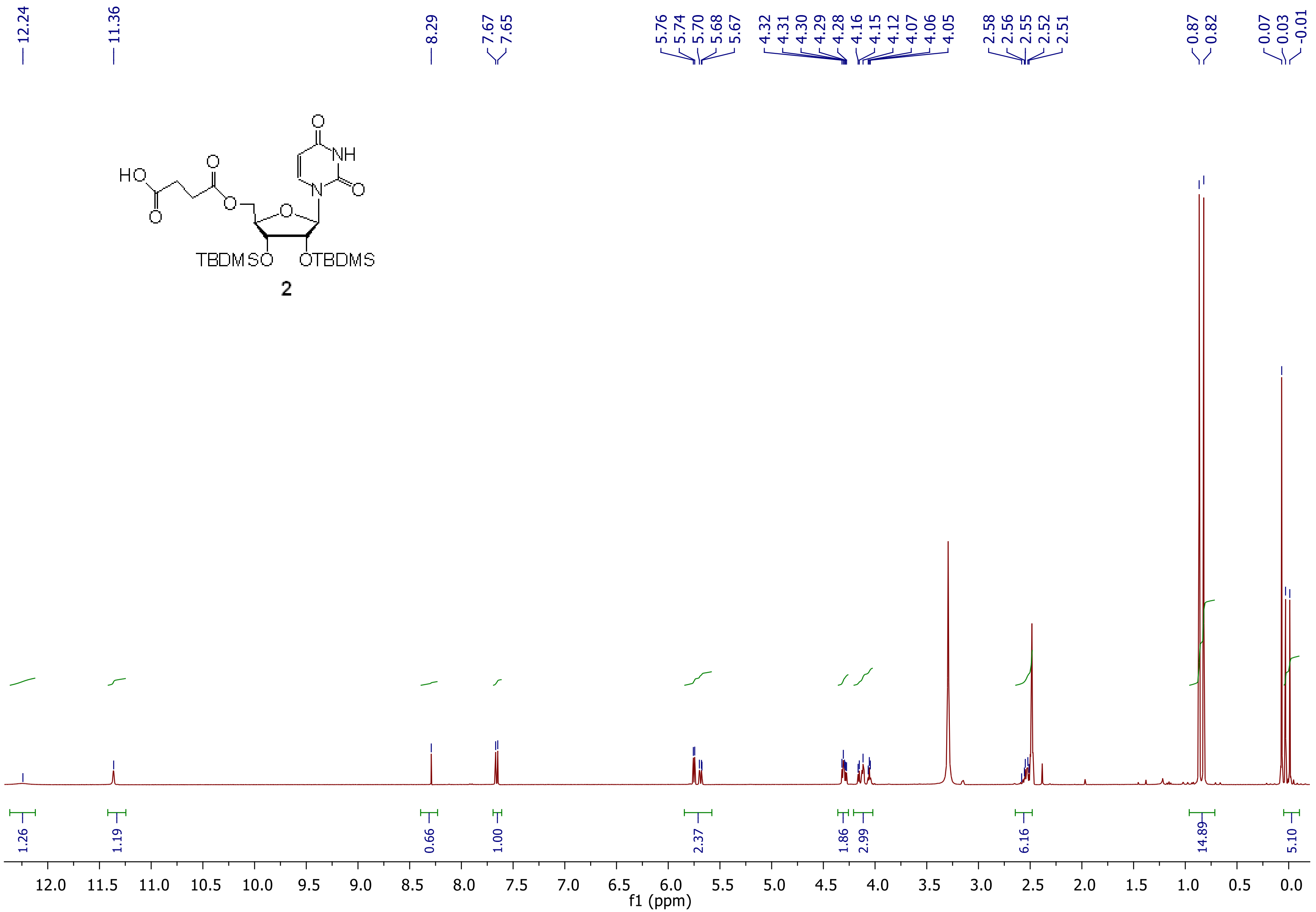

Fig. S3: ^{1}H NMR spectrum of succinic acid mono-2’,3’-di-*O*-*tert*-butyldimethylsilyl-uridin-5’-yl ester **2**.

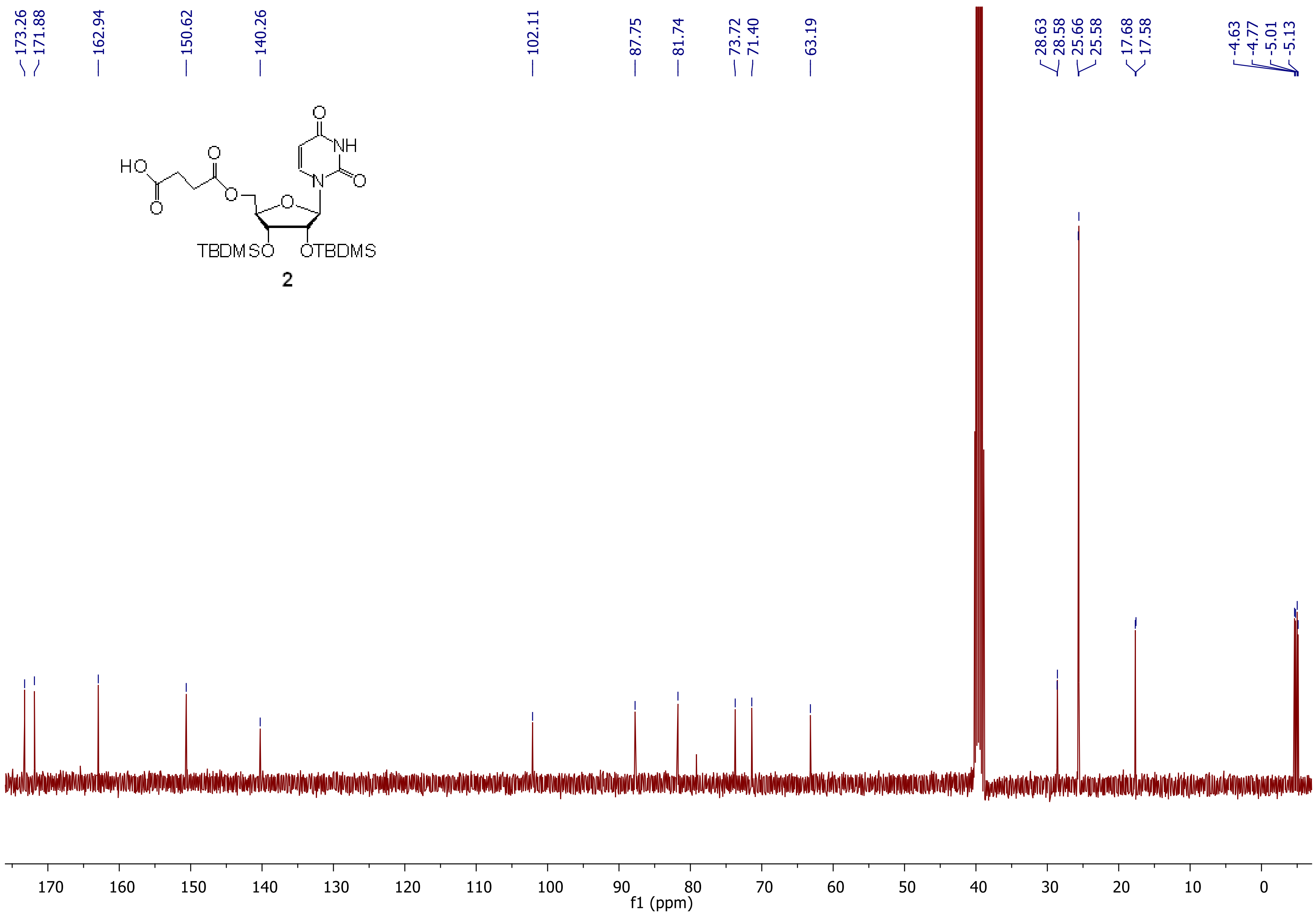

Fig. S4: ^{13}C NMR spectrum of succinic acid mono-2',3'-di-*O*-*tert*-butyldimethylsilyl-uridin-5'-yl ester **2**.

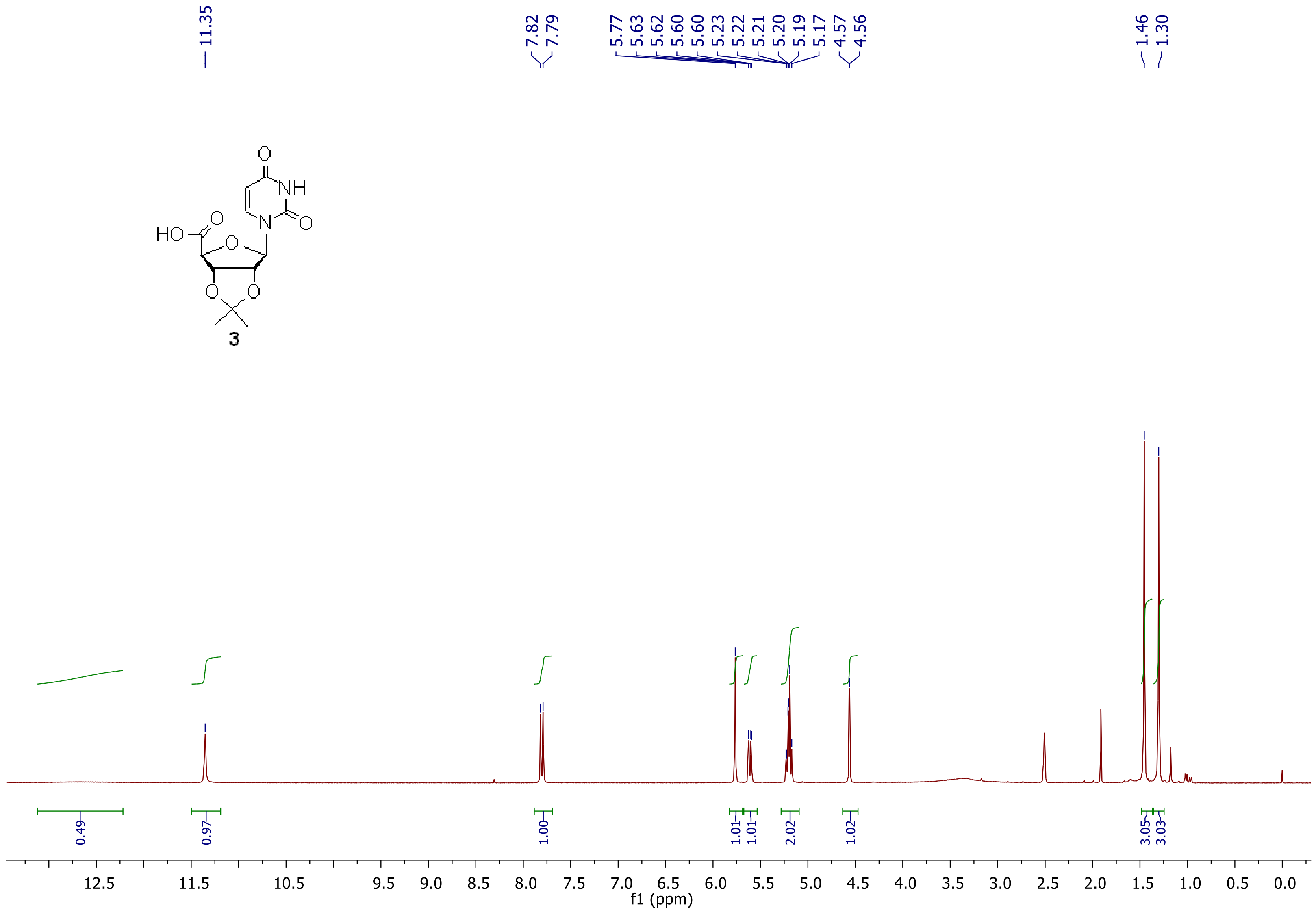

Fig. S5: ^{1}H NMR spectrum of 2',3'-*O*-isopropylideneuridine-5'-carboxylic acid **3**.

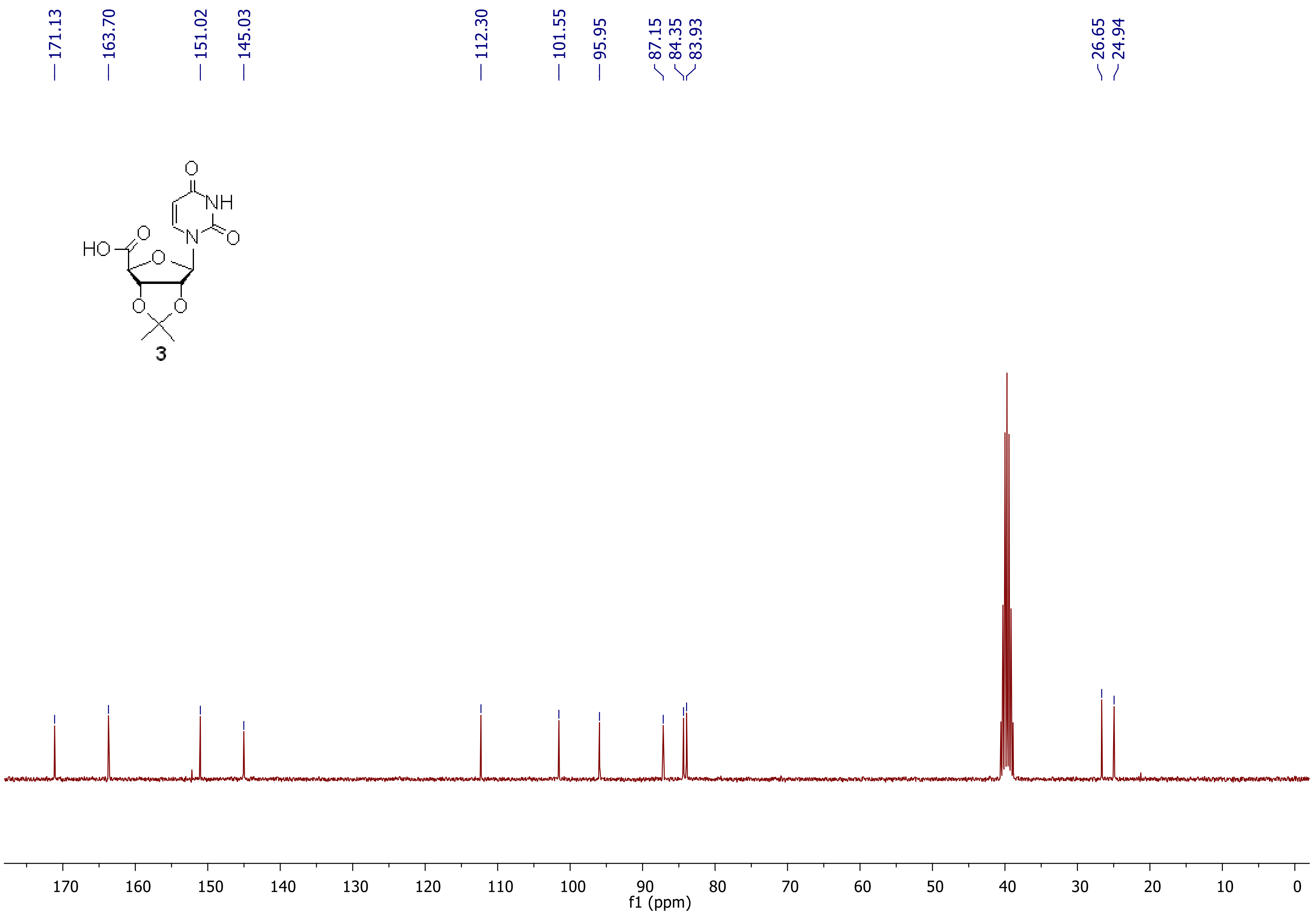

Fig. S6: ^{13}C NMR spectrum of 2’,3’-*O*-isopropylideneuridine-5’-carboxylic acid **3**.

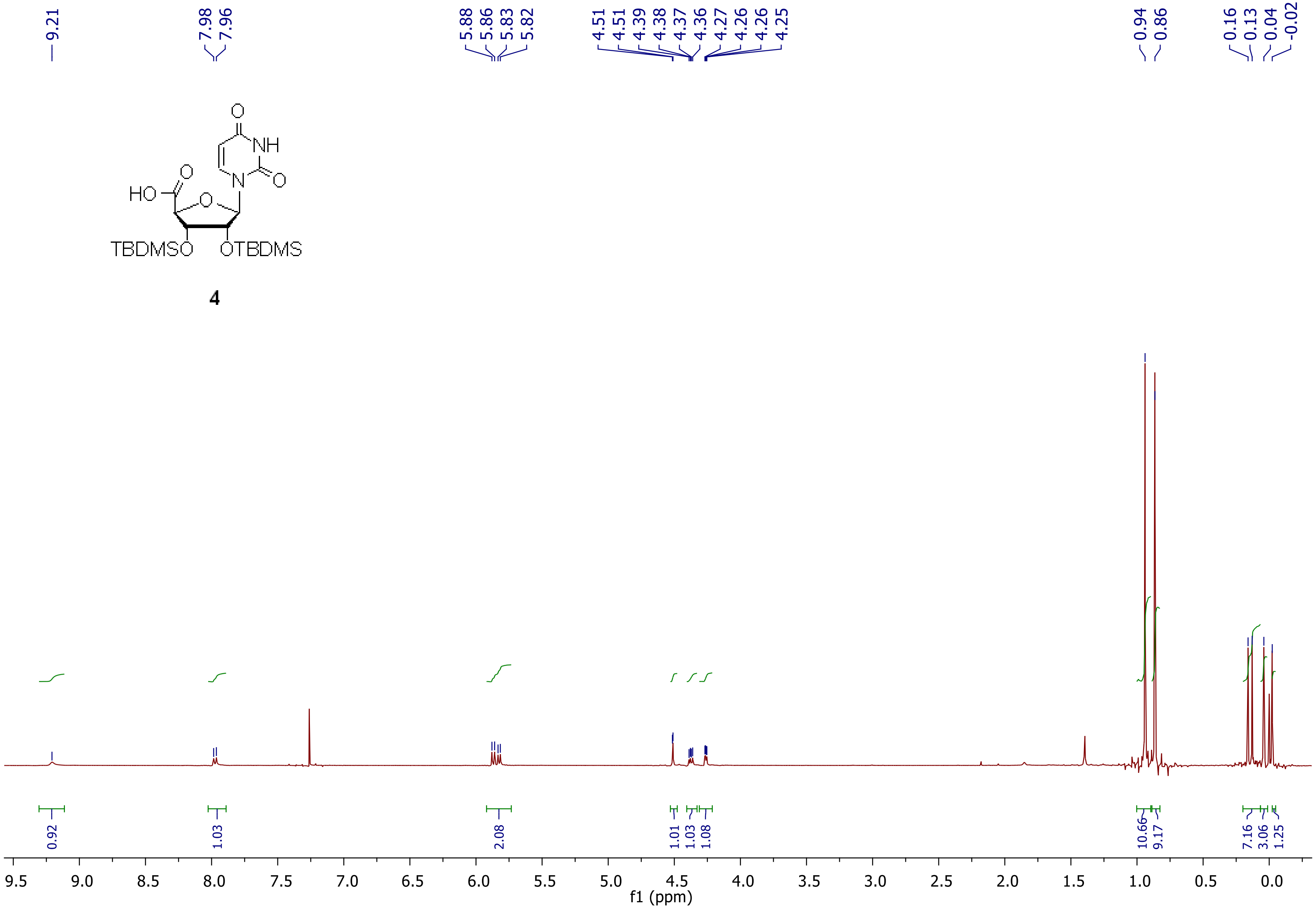

Fig. S7: ^{1}H NMR spectrum of 2’,3’-di-*O*-*tert*-butyldimethylsilyluridine-5’-carboxylic acid **4**.

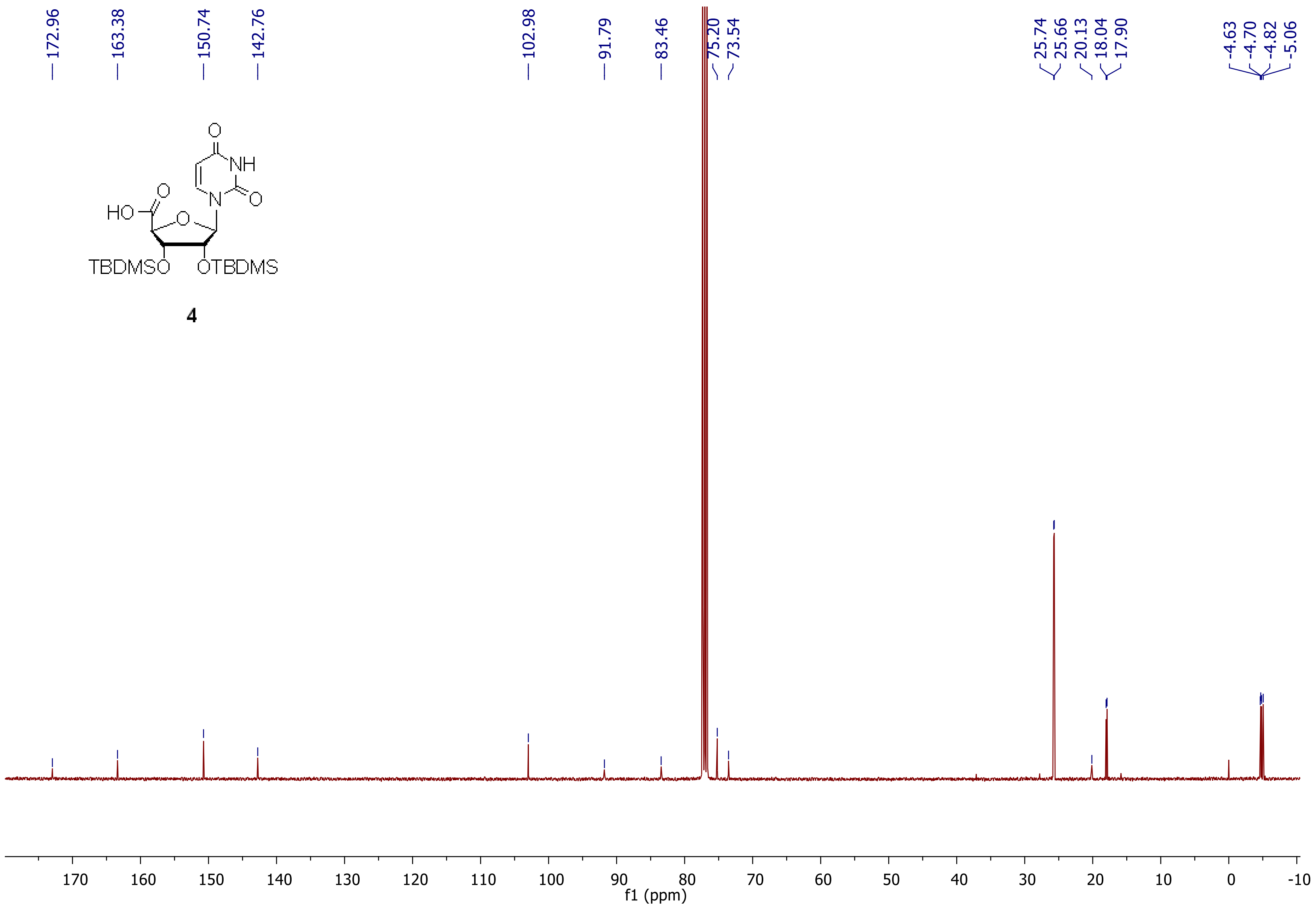

Fig. S8: ^{13}C NMR spectrum of 2’,3’-di-*O*-*tert*-butyldimethylsilyluridine-5’-carboxylic acid **4**.

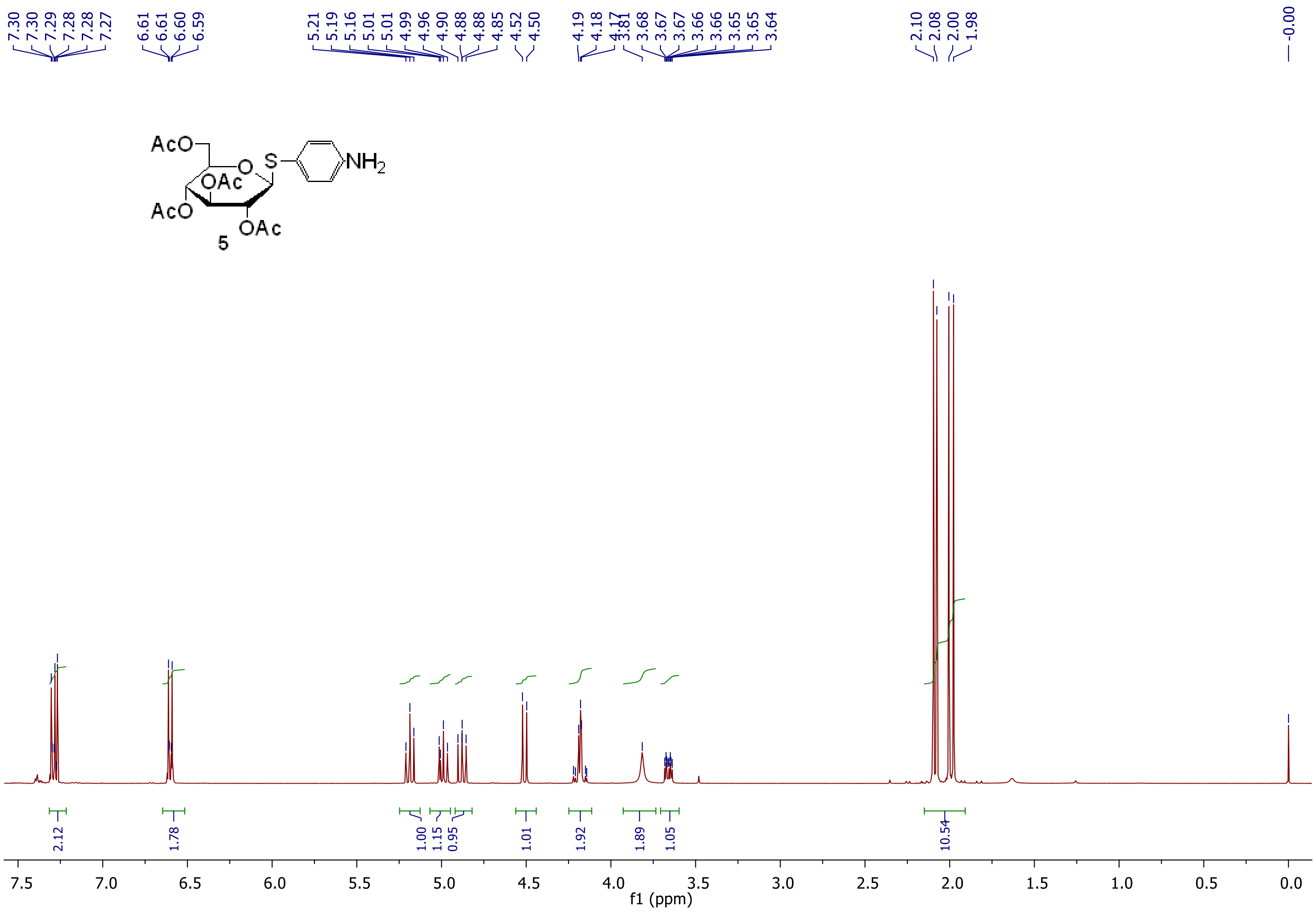

Fig. S9: ^{1}H NMR spectrum of 4-aminophenyl 2,3,4,6-tetra-O-acetyl-1-thio-β-D-glucopyranoside **5**.

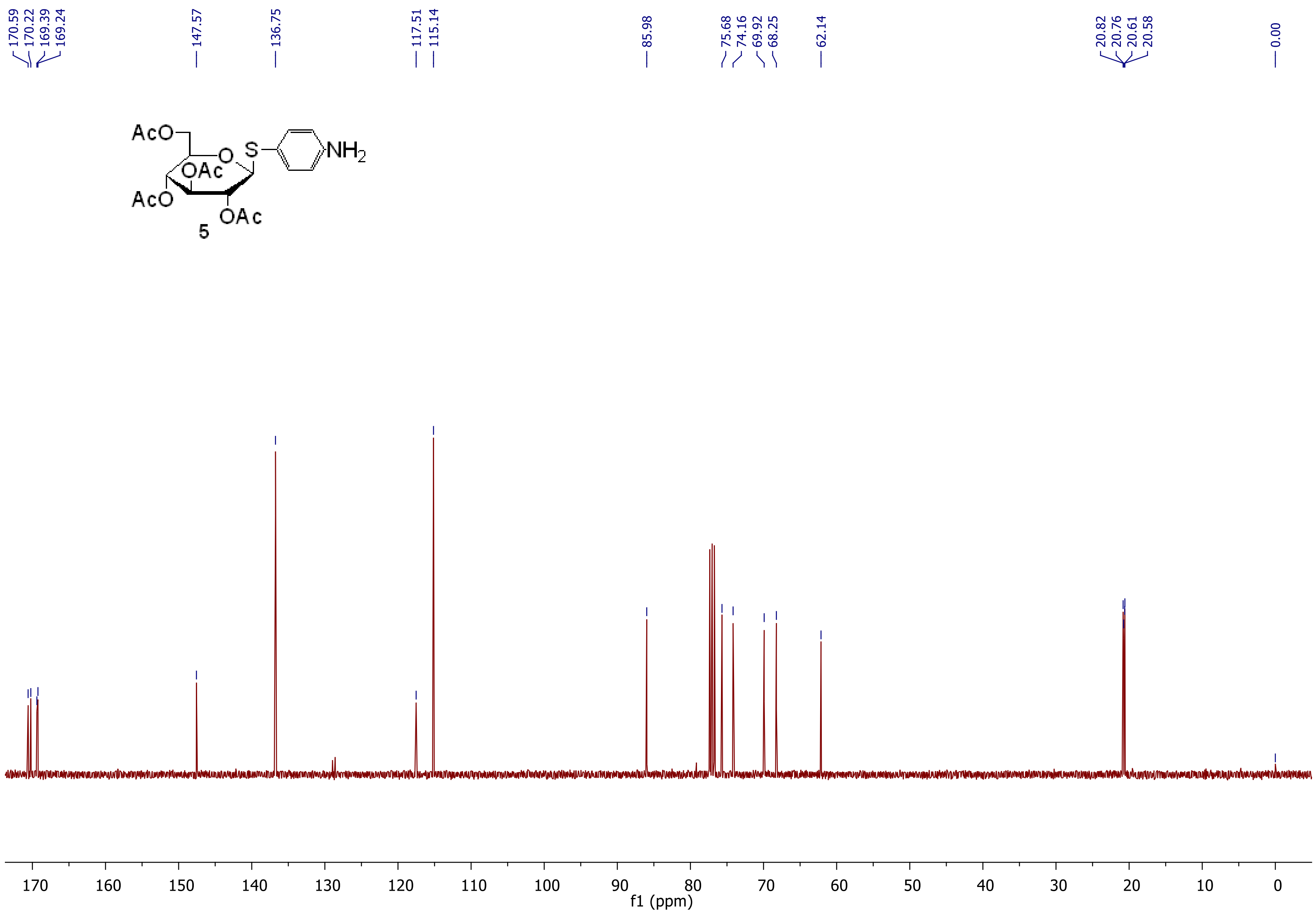

Fig. S10: ^{13}C NMR spectrum of 4-aminophenyl 2,3,4,6-tetra-O-acetyl-1-thio-β-D-glucopyranoside **5**.

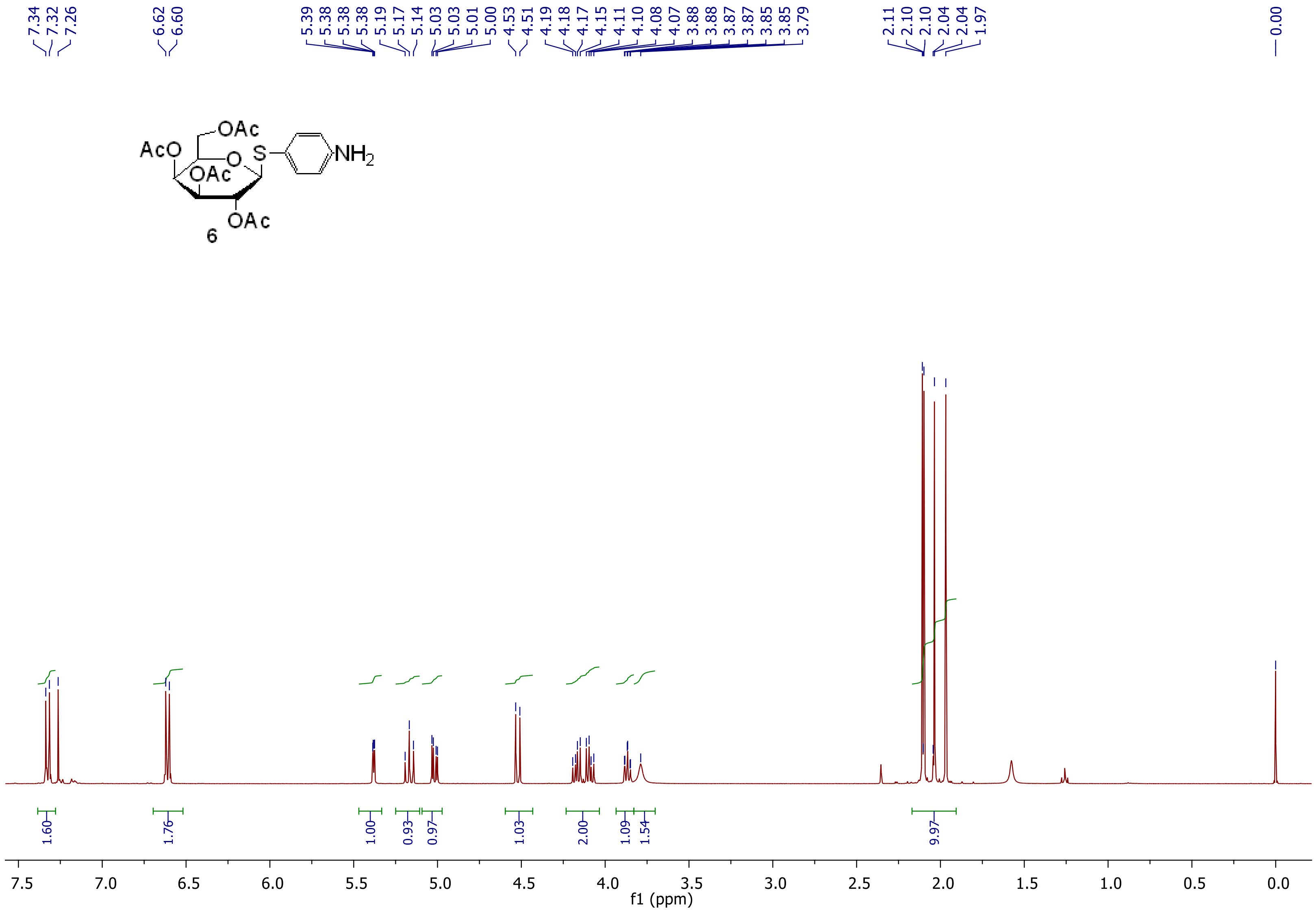

Fig. S11: ^{1}H NMR spectrum of 4-aminophenyl 2,3,4,6-tetra-O-acetyl-1-thio-β-D-galactopyranoside **6**.

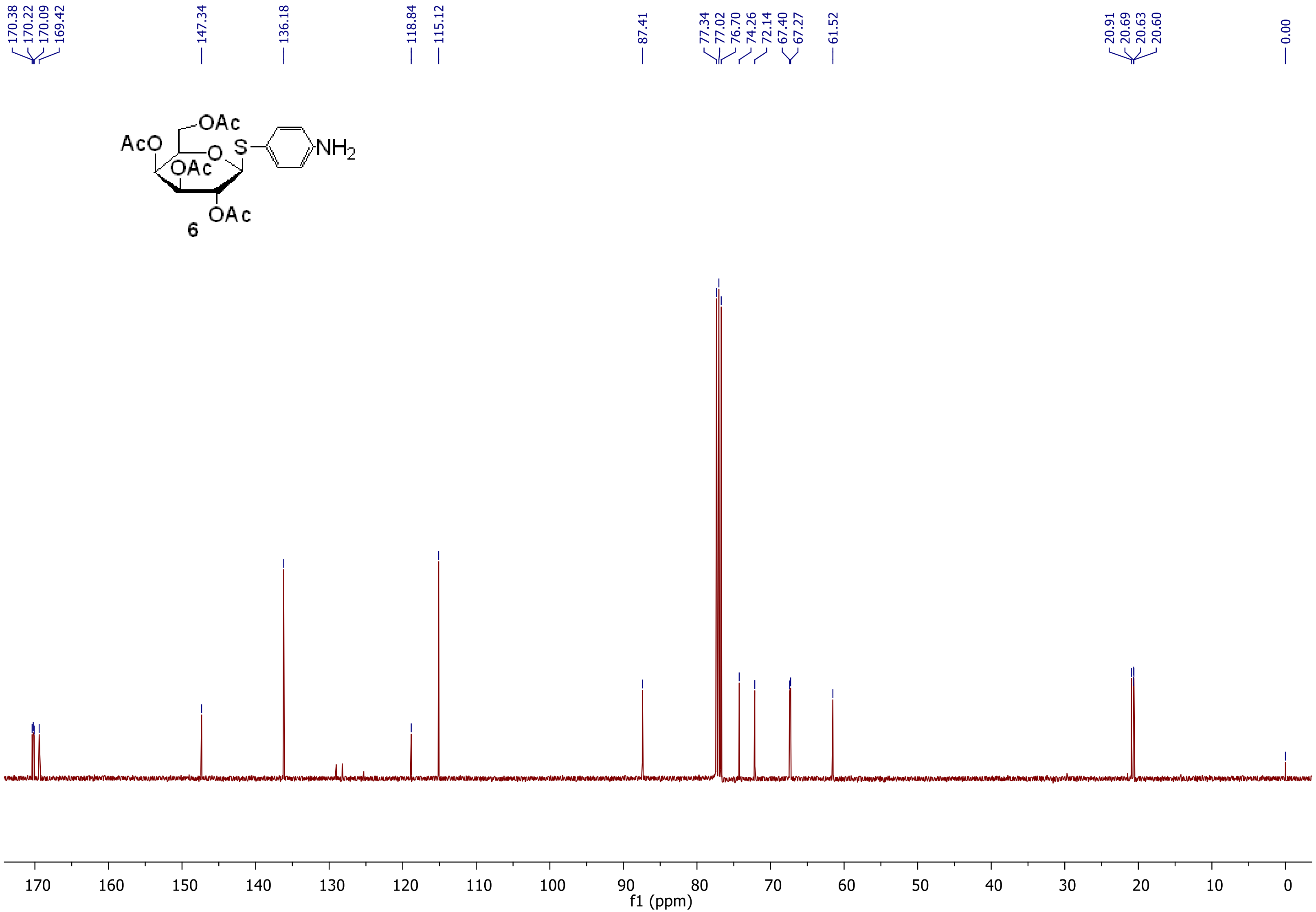

Fig. S12: ^{13}C NMR spectrum of 4-aminophenyl 2,3,4,6-tetra-O-acetyl-1-thio-β-D-galactopyranoside **6**.

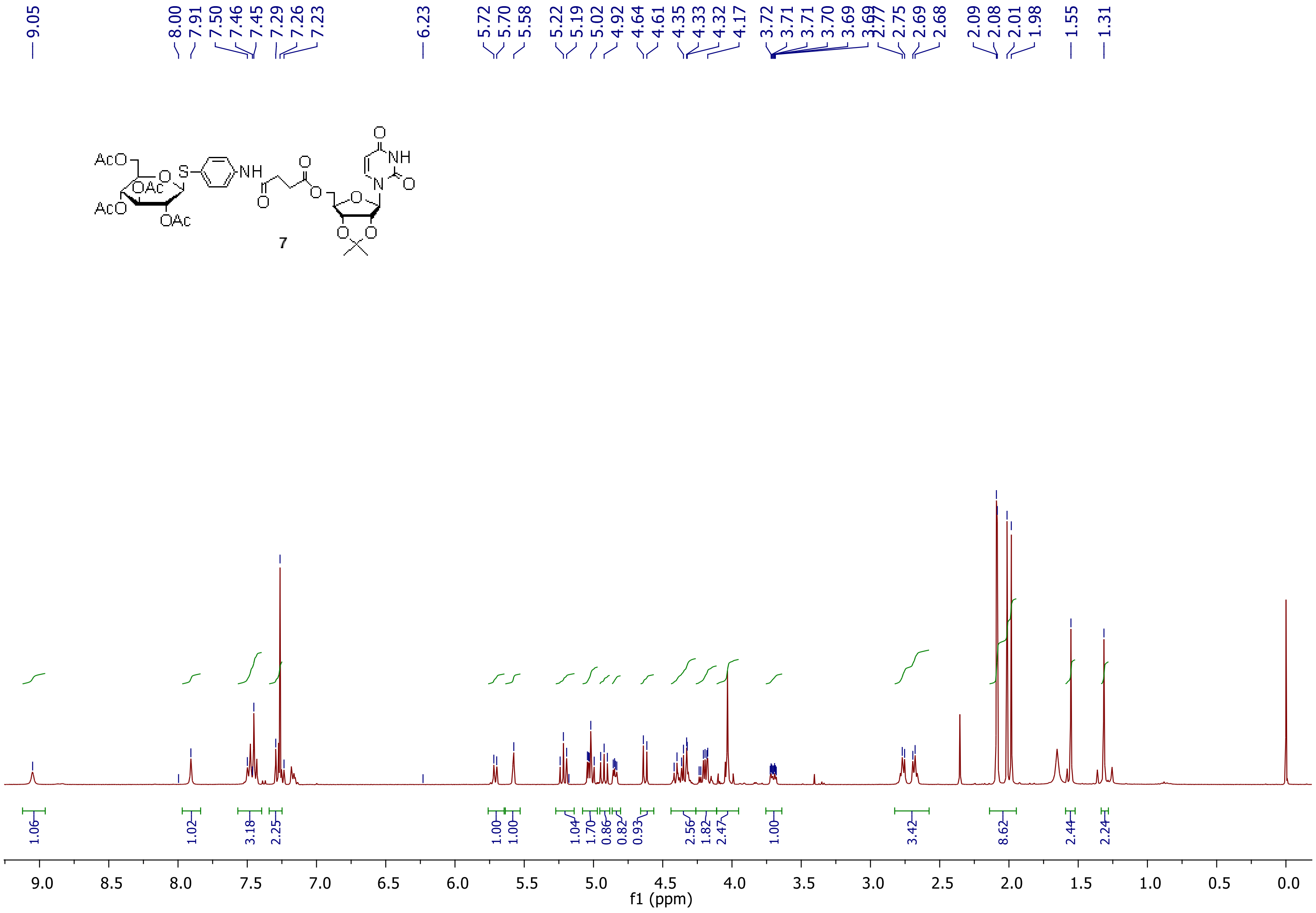

Fig. S13: ^{1}H NMR spectrum of glycoconjugate **7**.

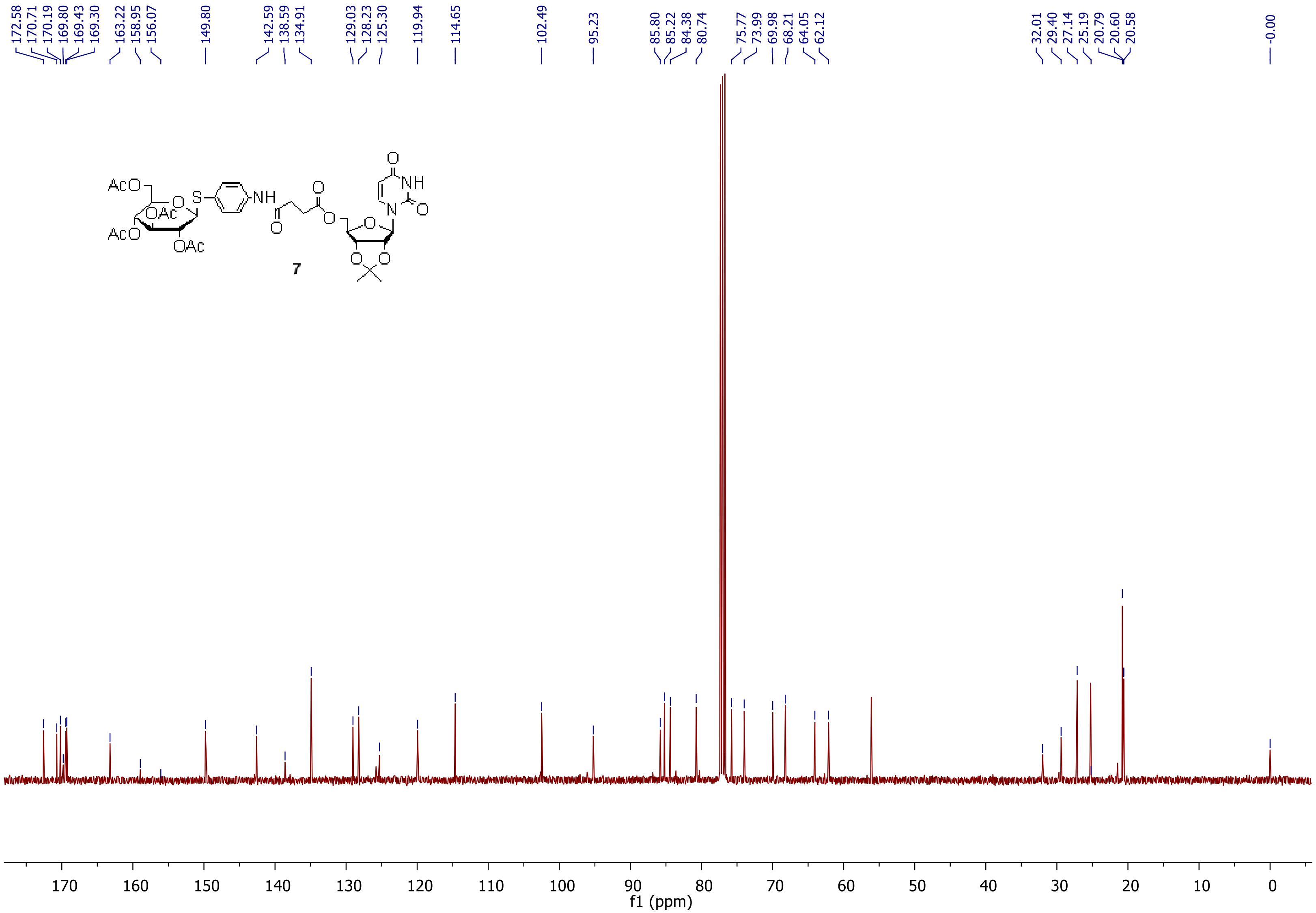

Fig. S14: ^{13}C NMR spectrum of glycoconjugate **7**.

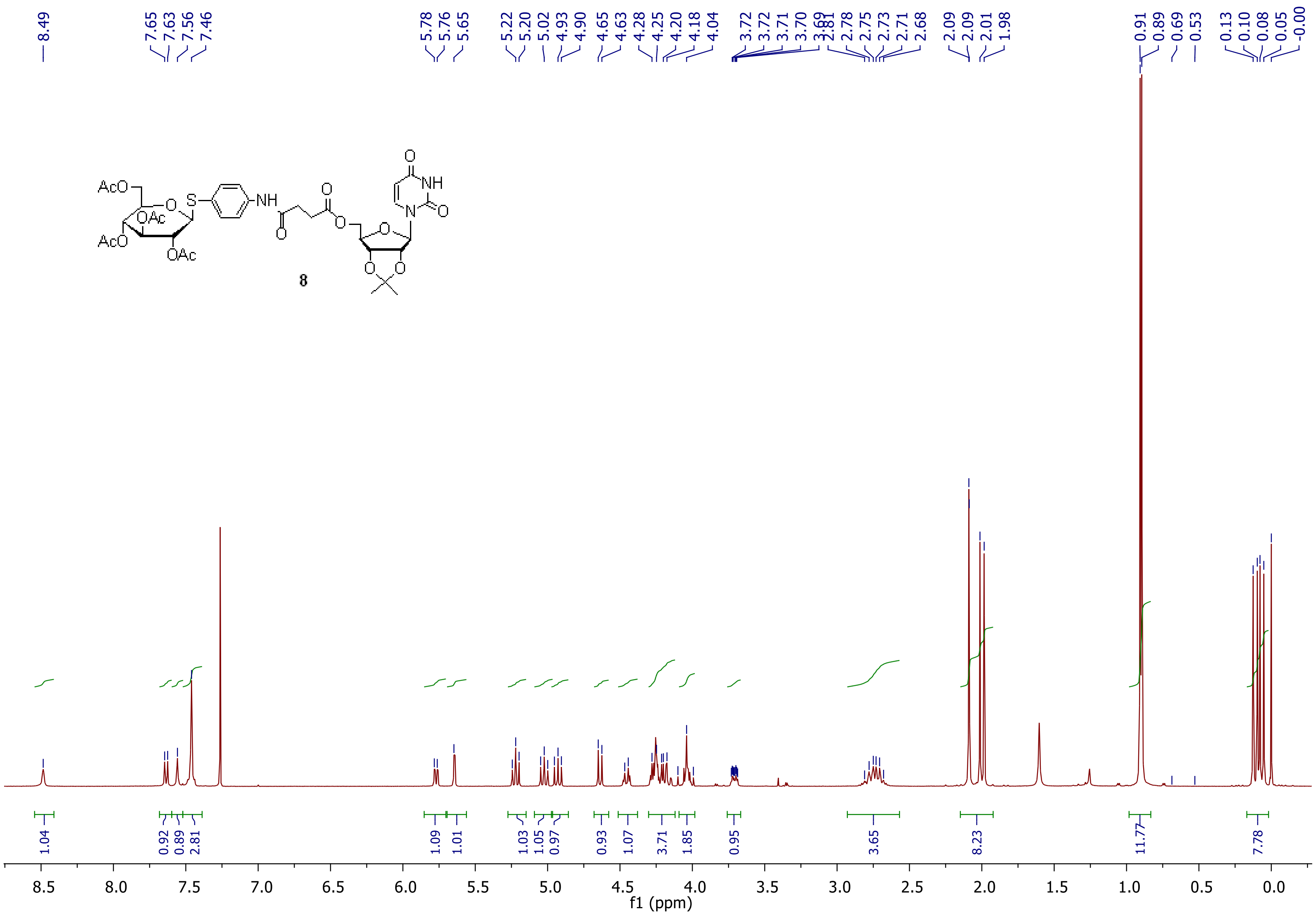

Fig. S15: ^{1}H NMR spectrum of glycoconjugate **8**.

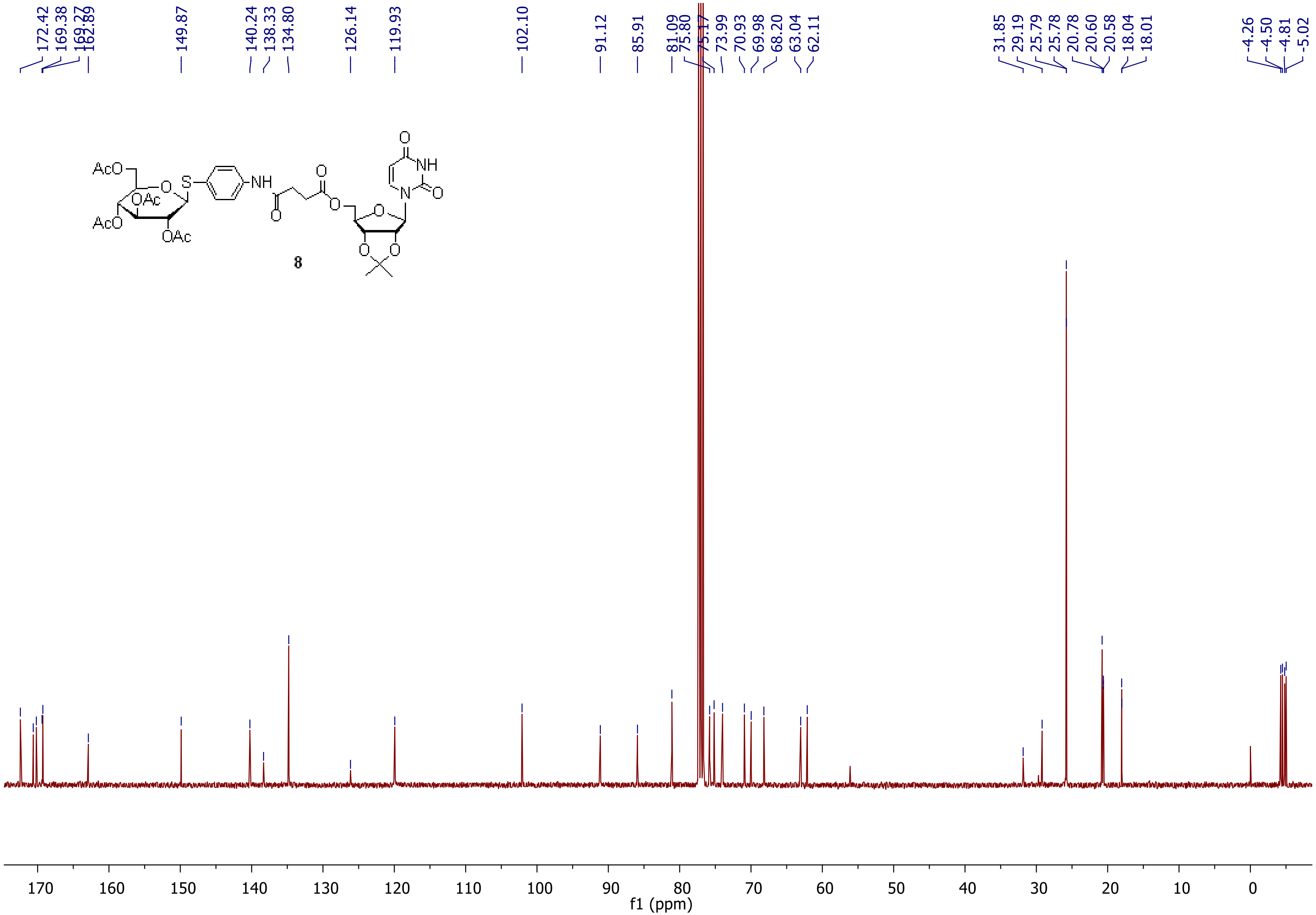

Fig. S16: ^{13}C NMR spectrum of glycoconjugate **8**.

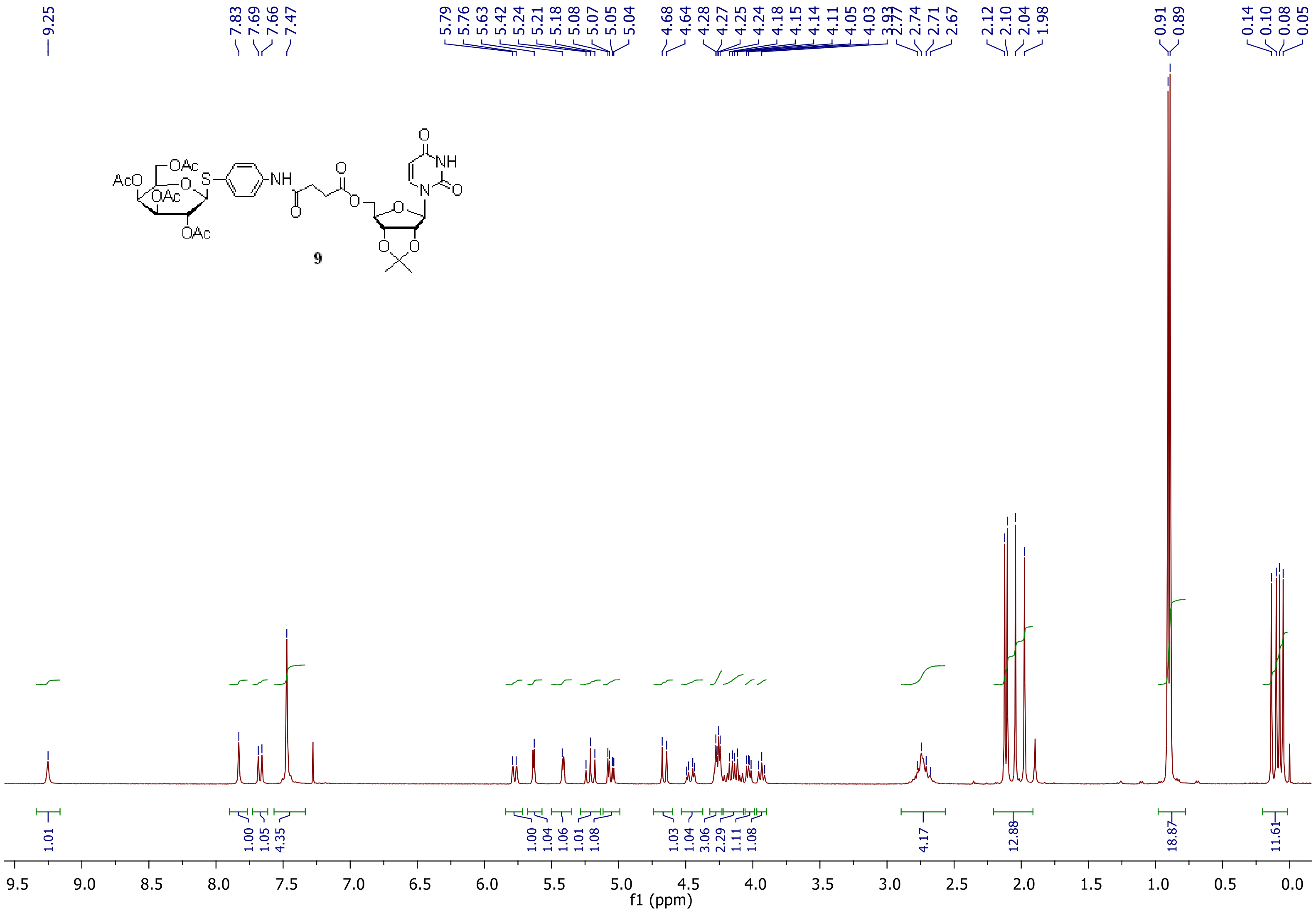

Fig. S17: ^{1}H NMR spectrum of glycoconjugate **9**.

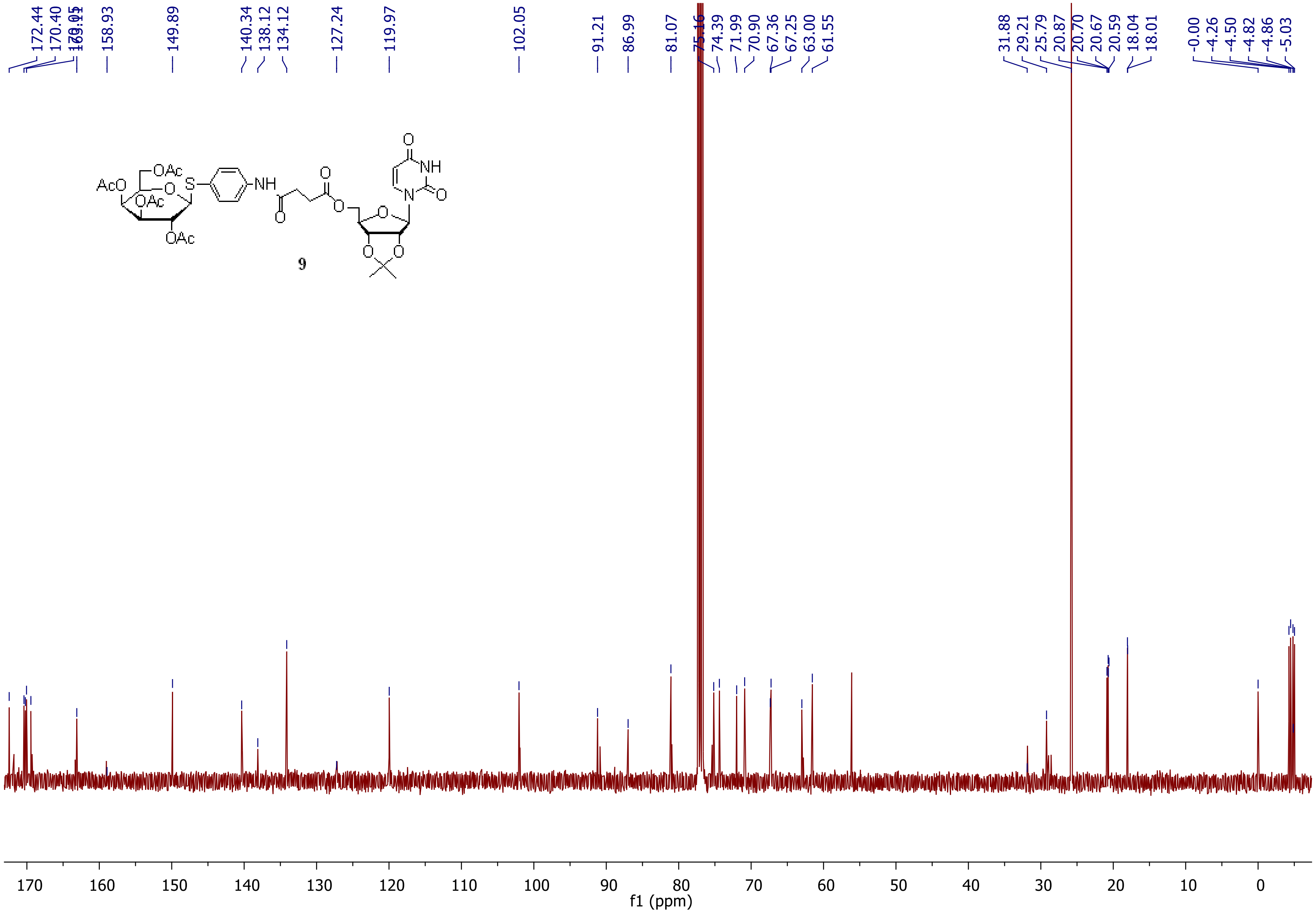

Fig. S18: ^{13}C NMR spectrum of glycoconjugate **9**.

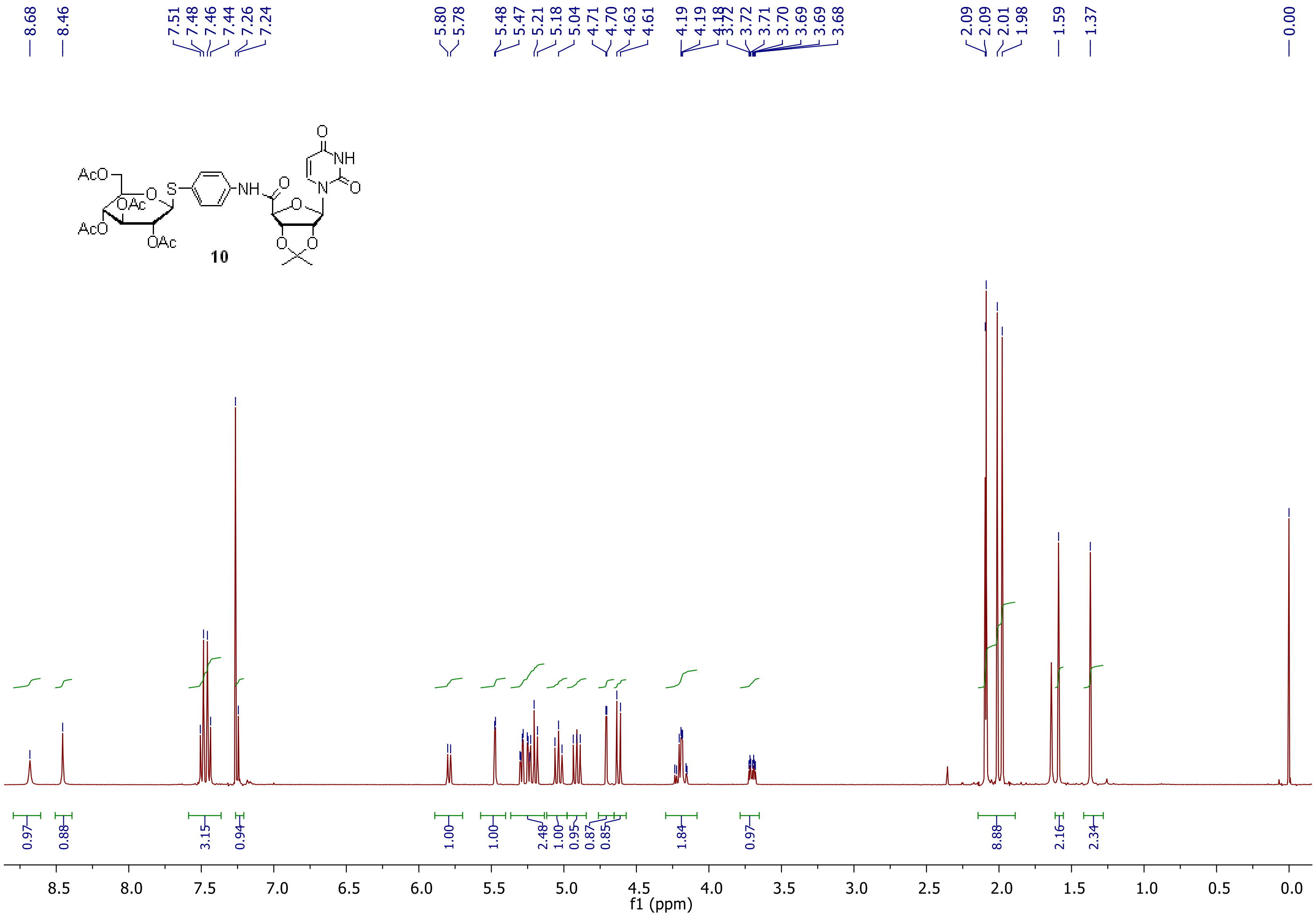

Fig. S19: ^{1}H NMR spectrum of glycoconjugate **10**.

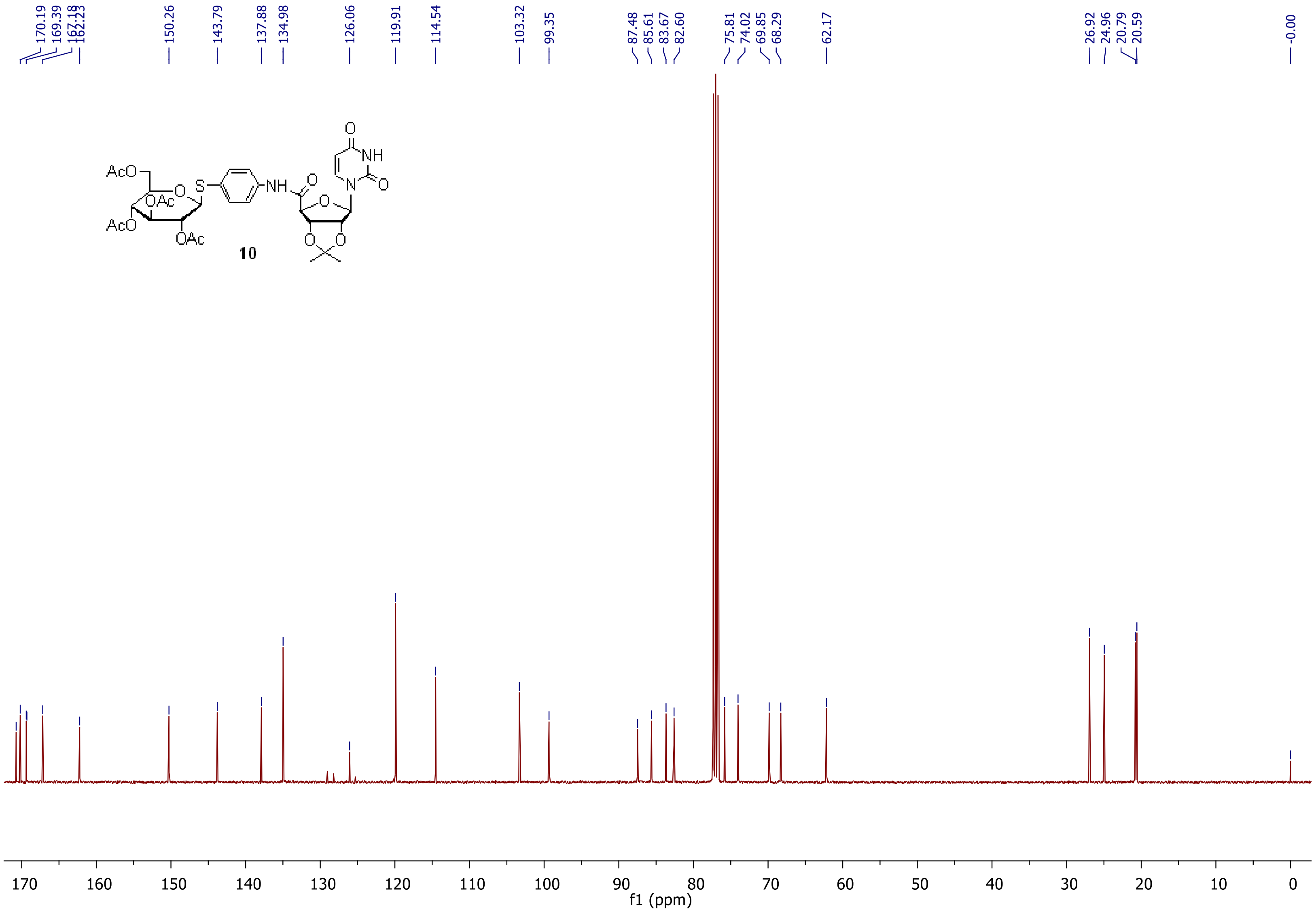

Fig. S20: ^{13}C NMR spectrum of glycoconjugate **10**.

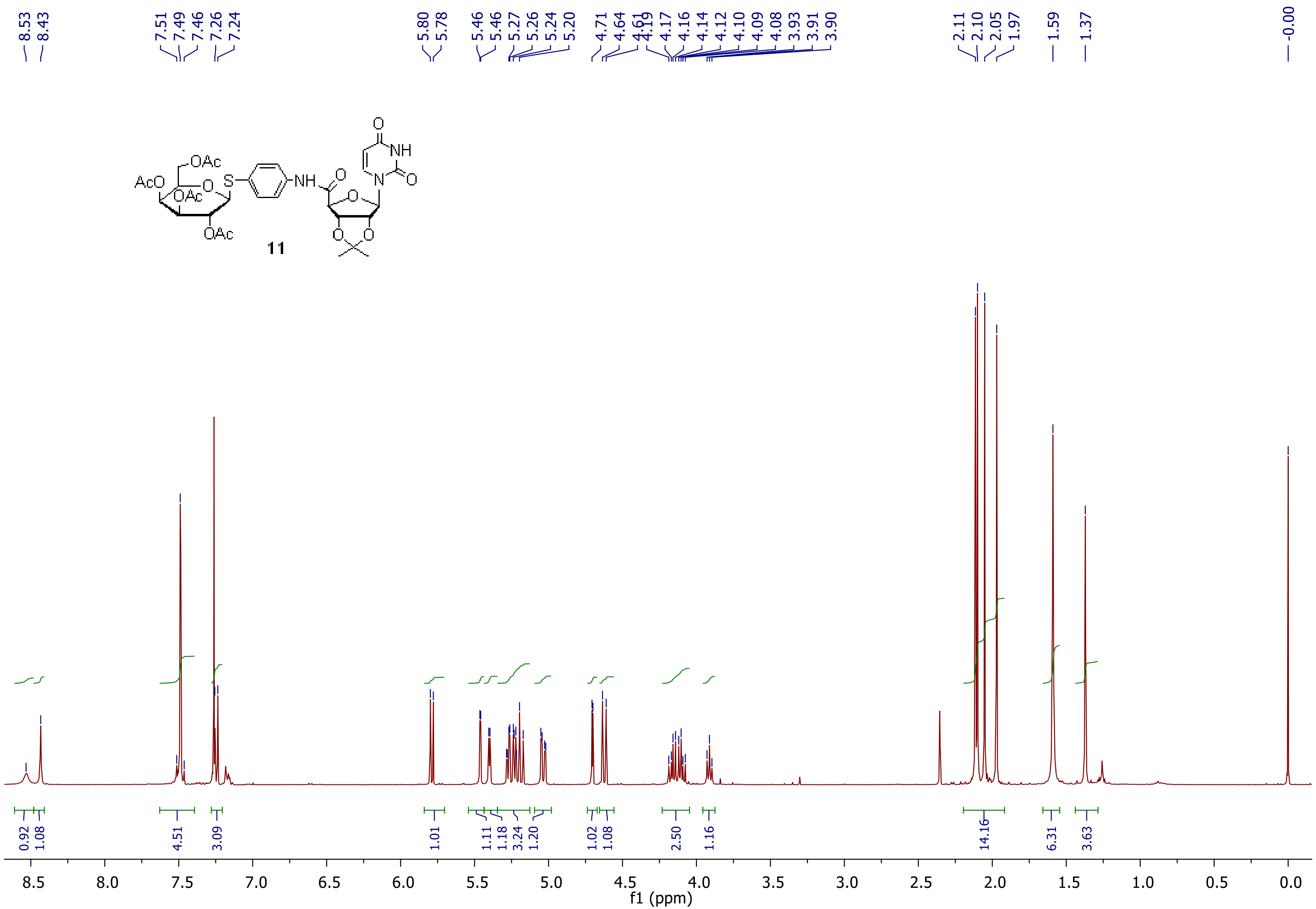

Fig. S21: ^{1}H NMR spectrum of glycoconjugate **11**.

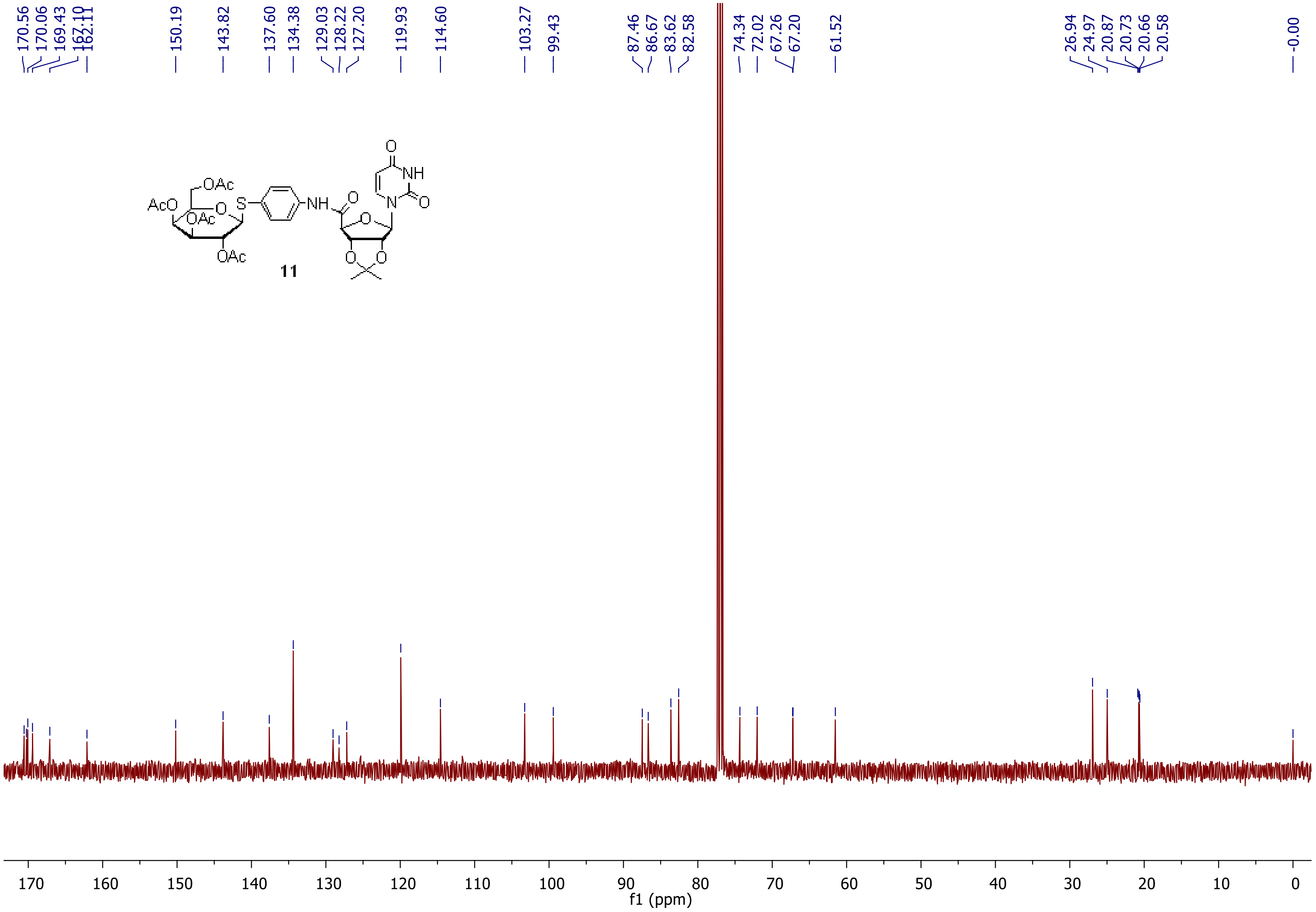

Fig. S22: ^{13}C NMR spectrum of glycoconjugate **11**.

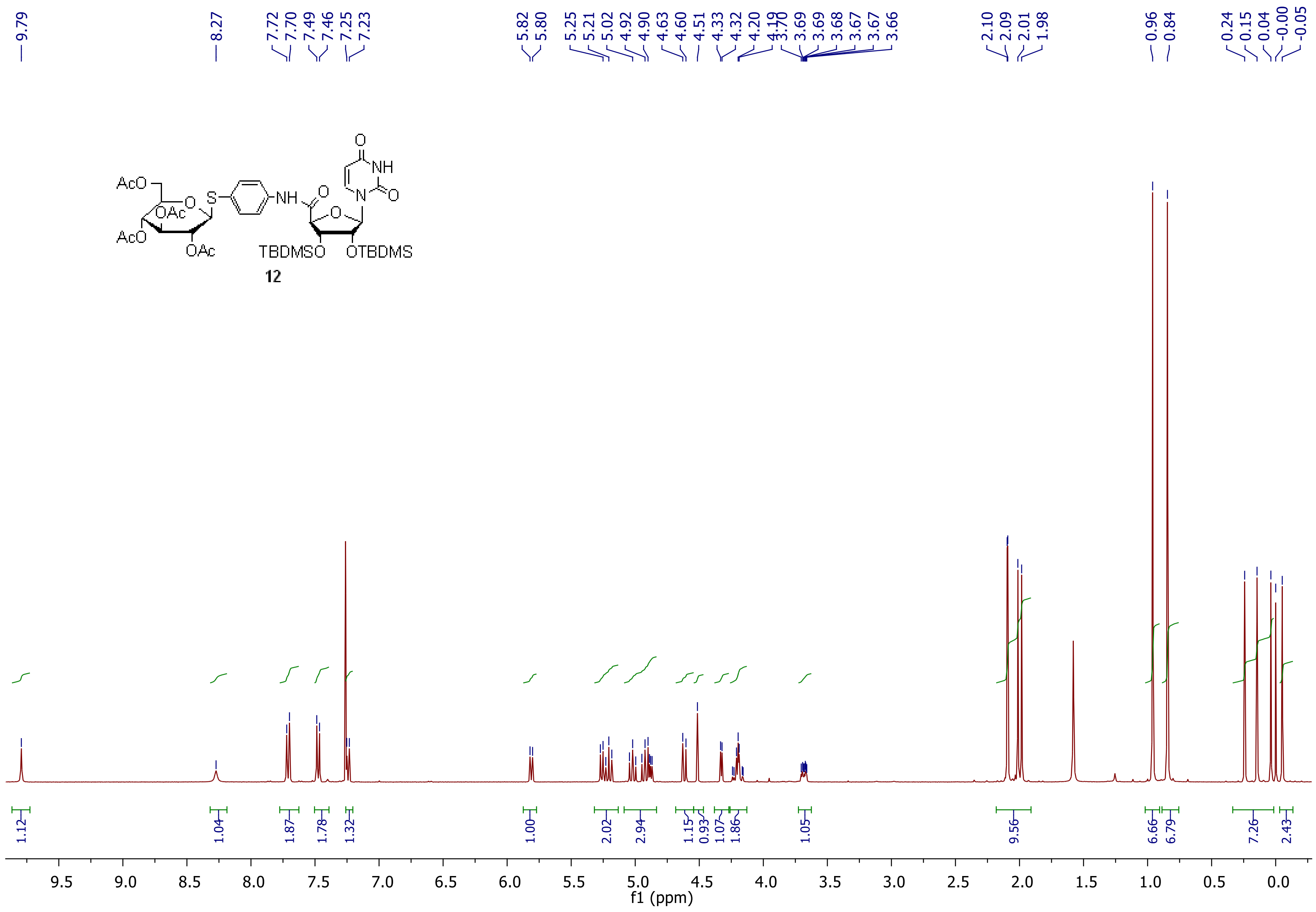

Fig. S23: ^{1}H NMR spectrum of glycoconjugate **12**.

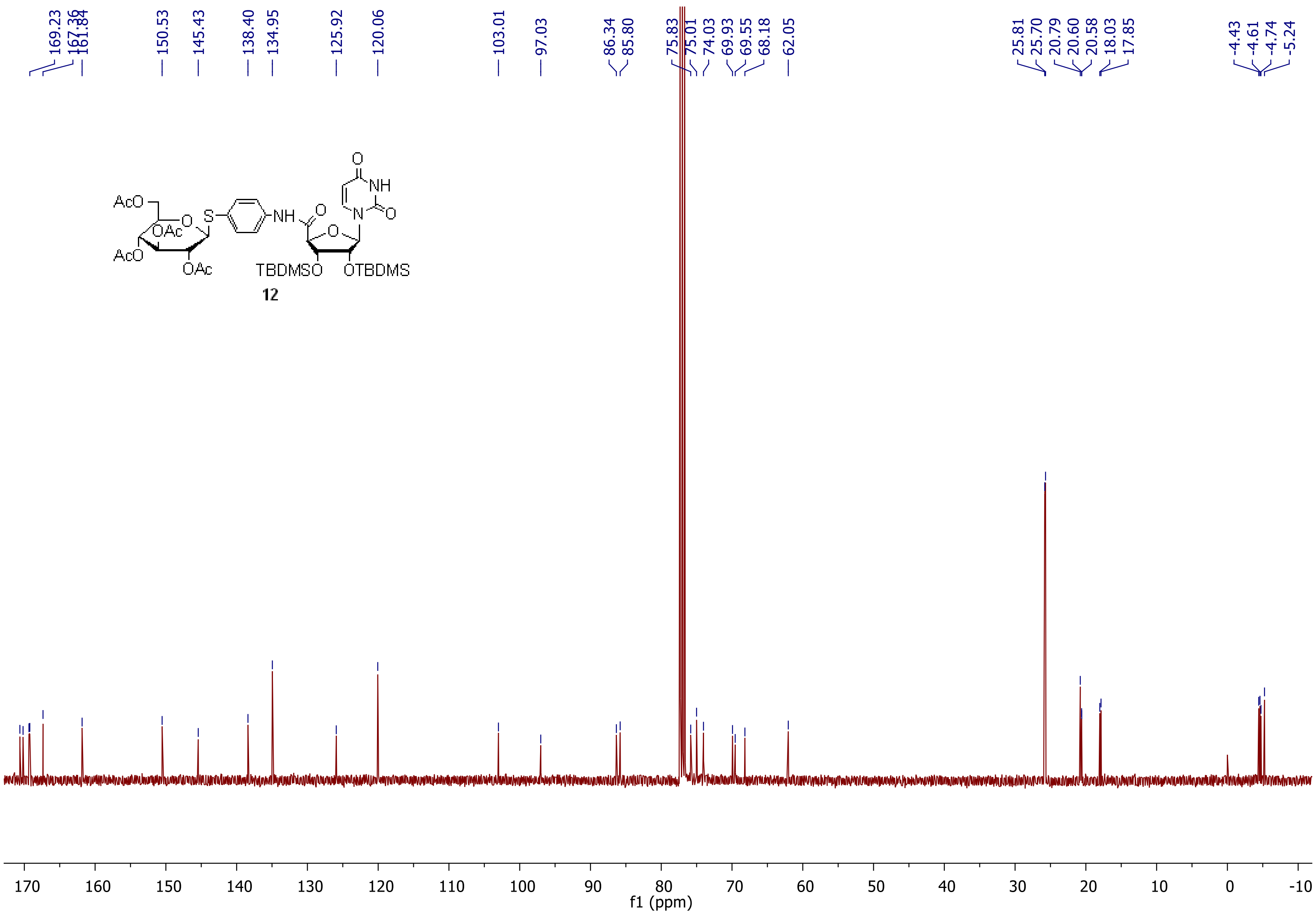

Fig. S24: ^{13}C NMR spectrum of glycoconjugate **12**.

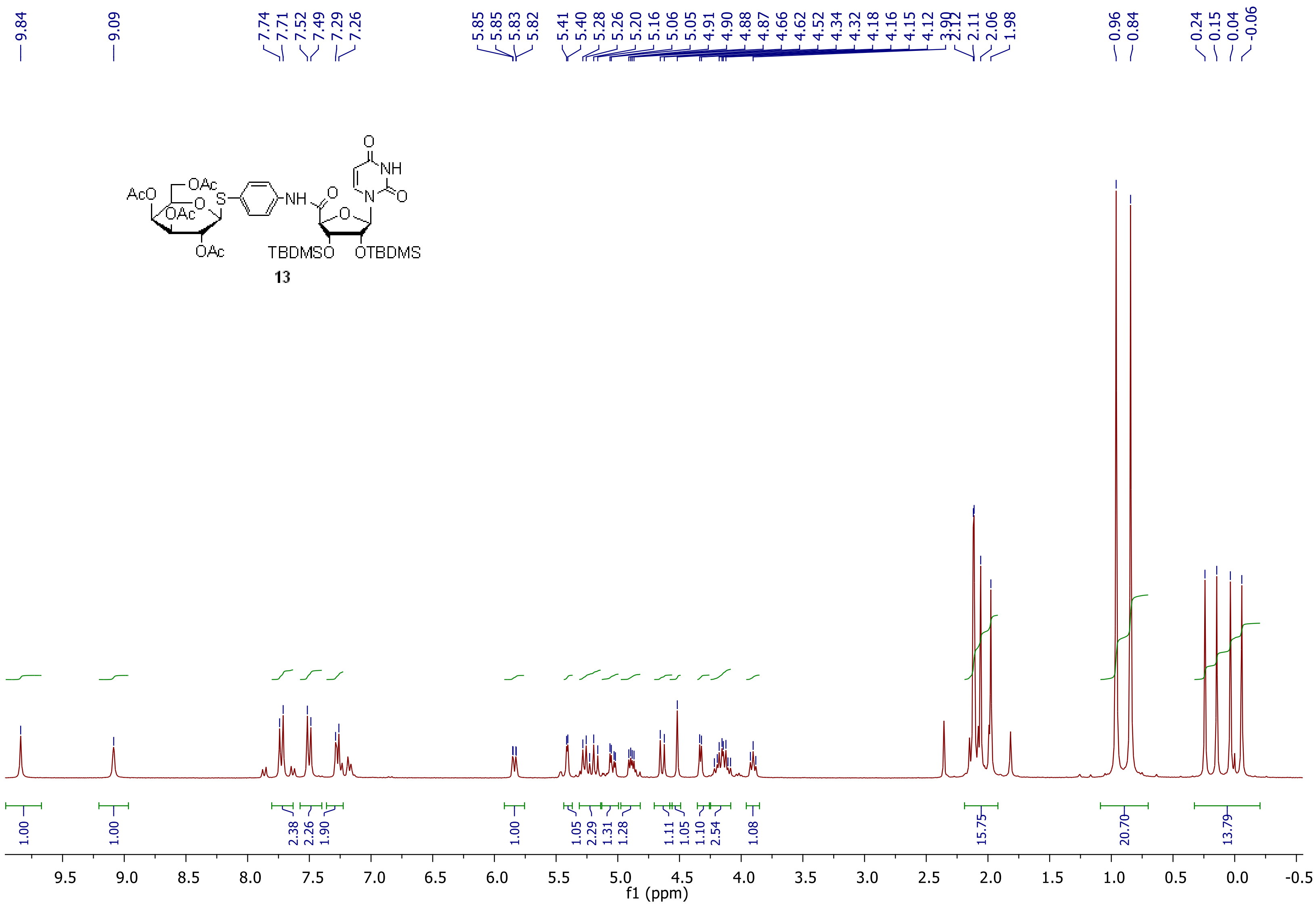

Fig. S25: ^{1}H NMR spectrum of glycoconjugate **13**.

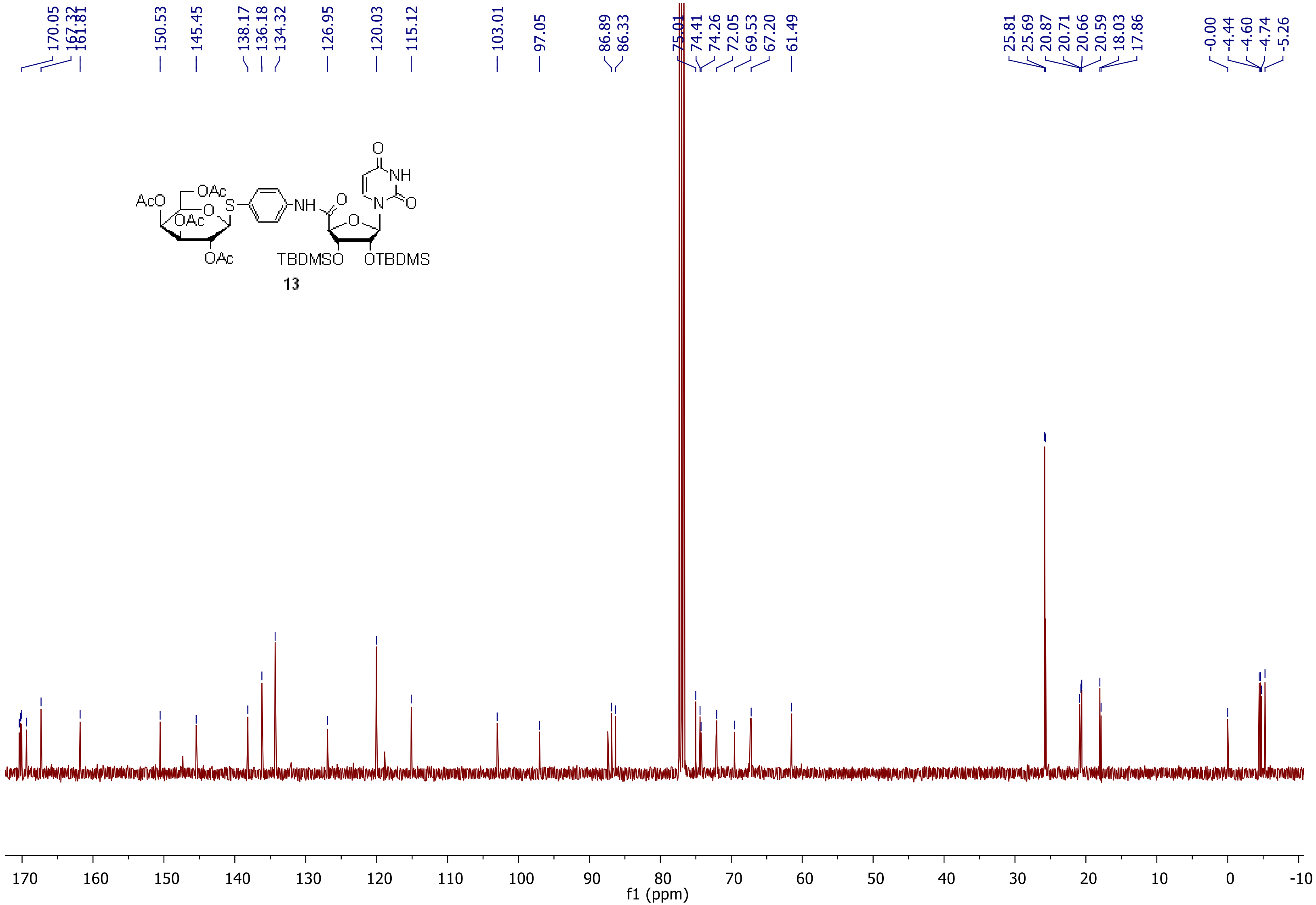

Fig. S26: ^{13}C NMR spectrum of glycoconjugate **13**.

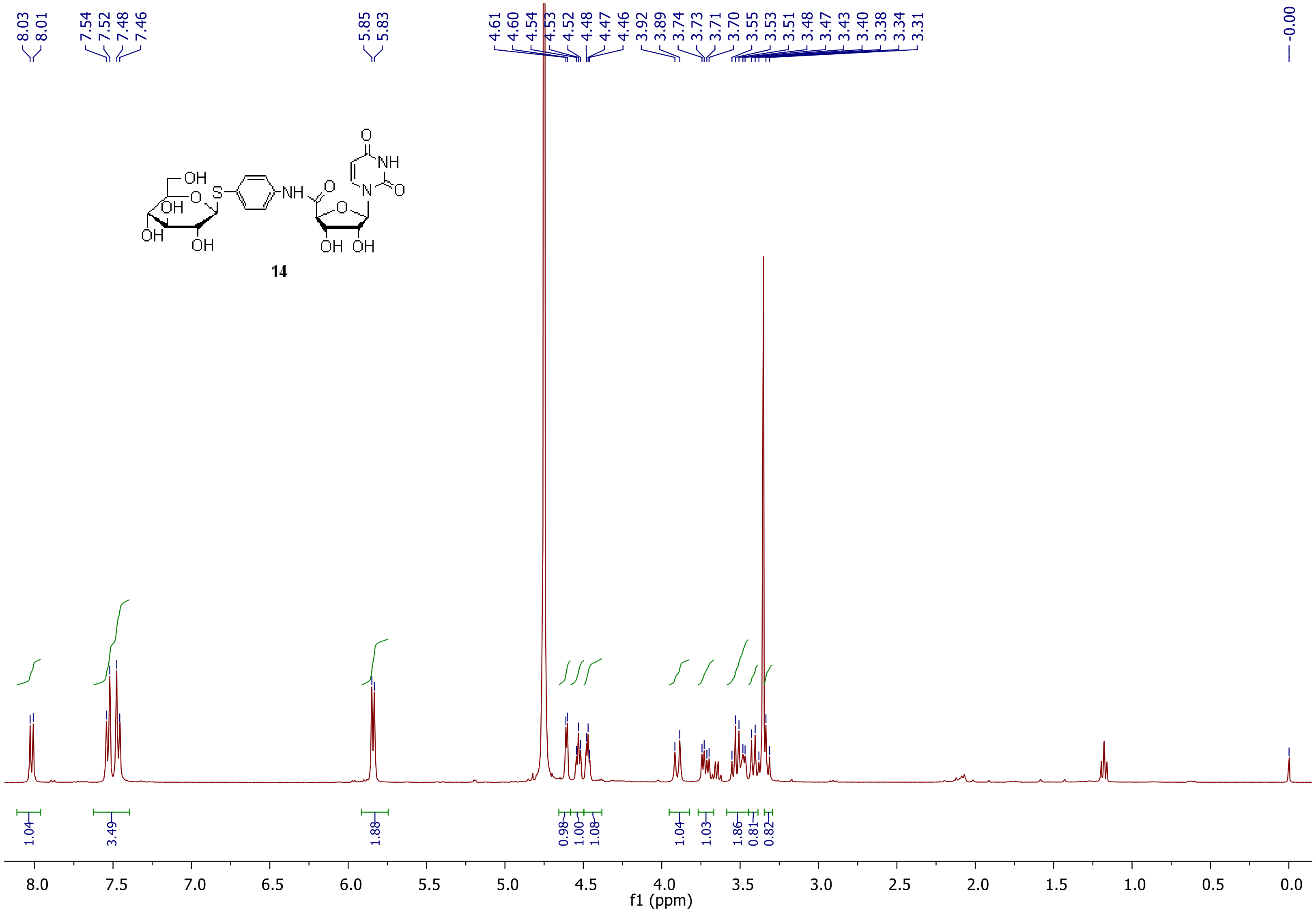

Fig. S27: ^{1}H NMR spectrum of glycoconjugate **14**.

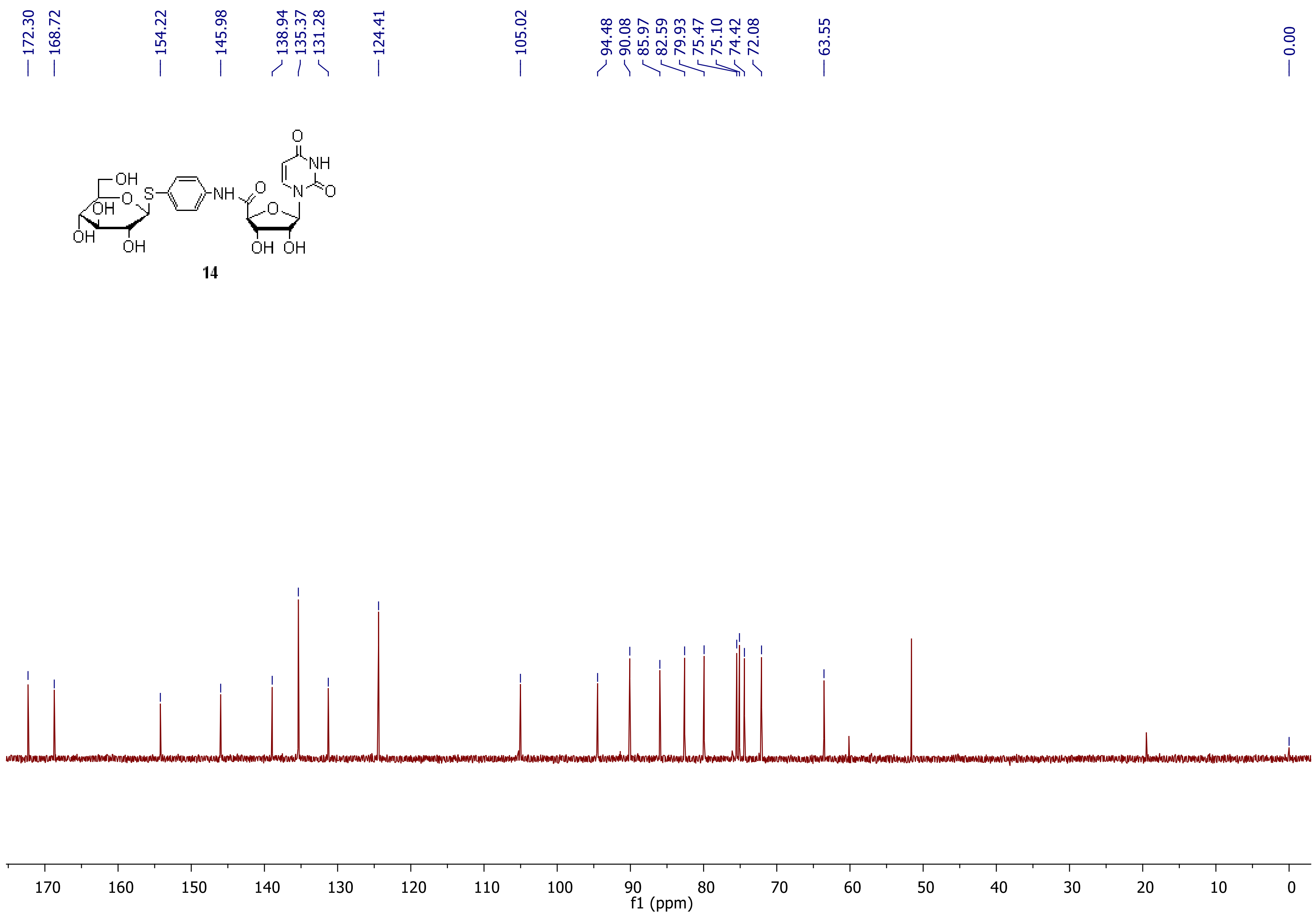

Fig. S28: ^{13}C NMR spectrum of glycoconjugate **14**.

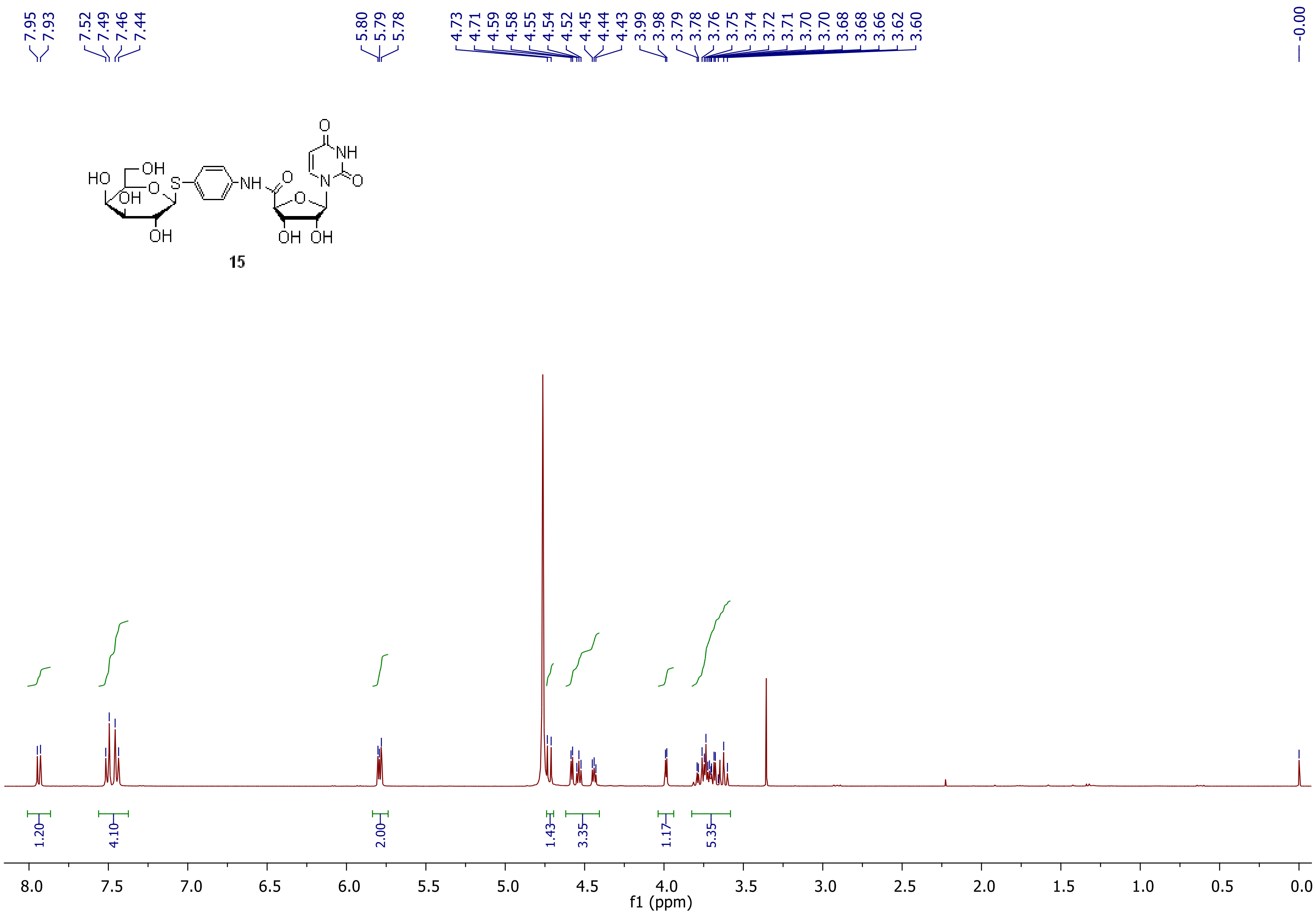

Fig. S29: ^{1}H NMR spectrum of glycoconjugate **15**.

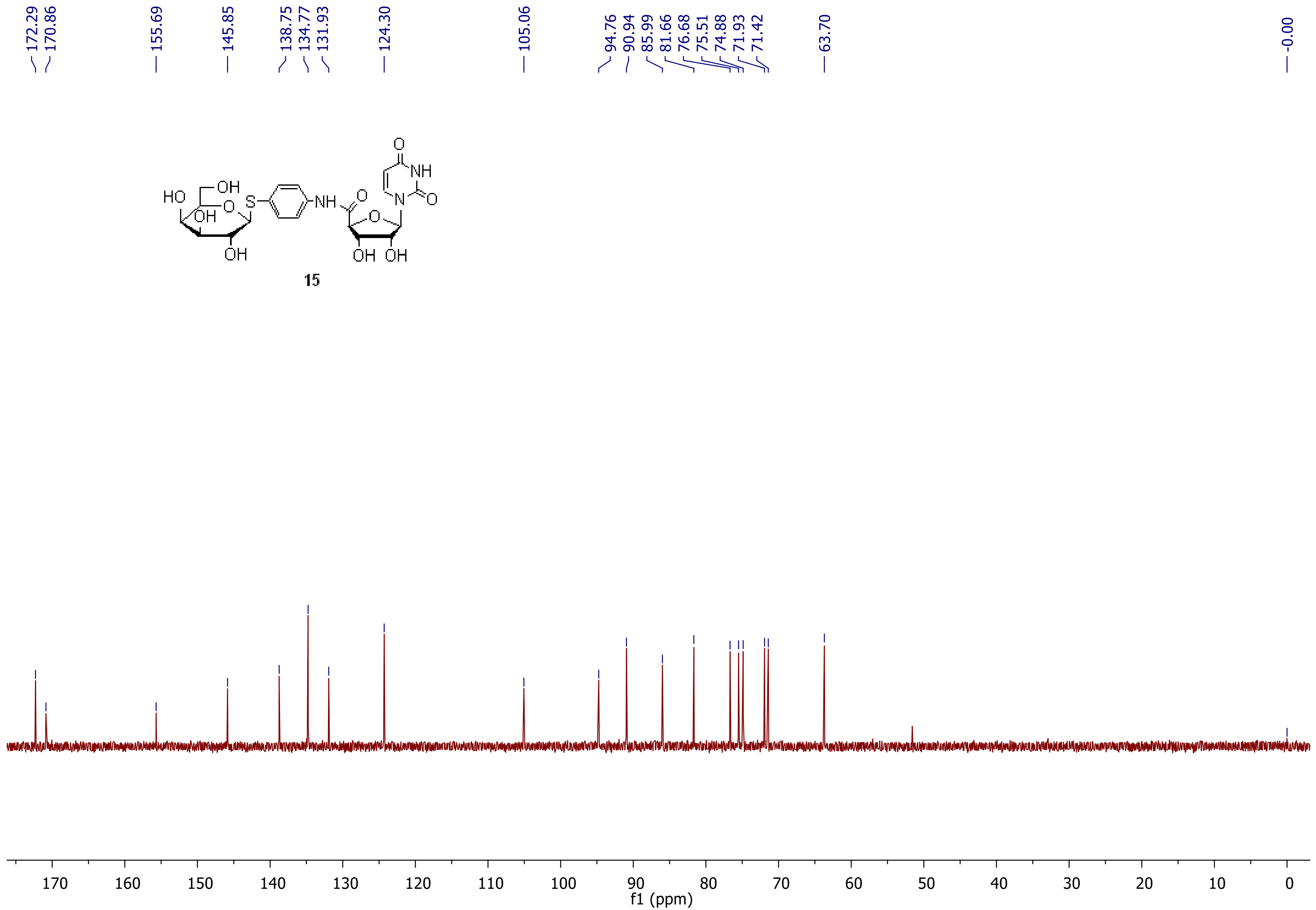

Fig. S30: ^{13}C NMR spectrum of glycoconjugate **15**.

2. Antiviral activity of Sofosbuvir on HCV infection.

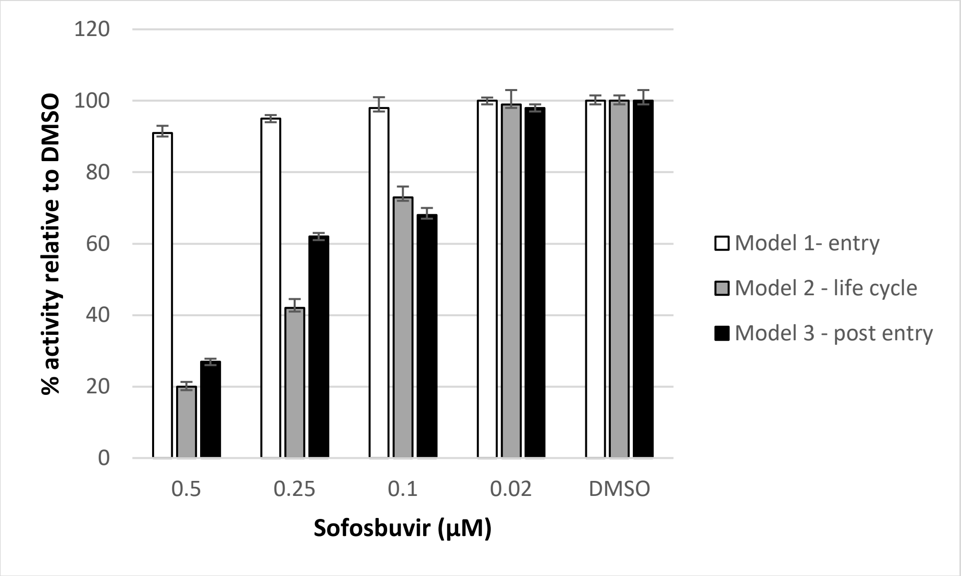

Fig. S31. Huh7-J20 cells were pre-treated for 1 h and infected with cell culture infectious HCV in the presence of different concentrations of Sofosbuvir or DMSO as a control for 3 h. Then, the inoculum was removed and fresh medium without compound was added for 72 h (Model 1, white bars). Huh7-J20 cells were pre-treated for 1 h, infected with JFH-1 for 3 h in the presence of various concentrations of Sofosbuvir or DMSO and then incubated for 72 h with fresh medium with inhibitor or DMSO (Model 2, grey bars). Huh7-J20 cells were infected for 3 h with JFH-1 and then treated with various concentrations of Sofosbuvir or DMSO for 72 h (Model 3, black bars). All inhibitory effects were determined by measuring SEAP assay performed on infected cell medium. Errors bars represent the SD of the means for 3 experiments.
